# graph-GPA 2.0: improving multi-disease genetic analysis with integration of functional annotation data

**DOI:** 10.3389/fgene.2023.1079198

**Published:** 2023-07-12

**Authors:** Qiaolan Deng, Arkobrato Gupta, Hyeongseon Jeon, Jin Hyun Nam, Ayse Selen Yilmaz, Won Chang, Maciej Pietrzak, Lang Li, Hang J. Kim, Dongjun Chung

**Affiliations:** ^1^ The Interdisciplinary PhD Program in Biostatistics, The Ohio State University, Columbus, OH, United States; ^2^ Department of Biomedical Informatics, The Ohio State University, Columbus, OH, United States; ^3^ Pelotonia Institute for Immuno-Oncology, The James Comprehensive Cancer Center, The Ohio State University, Columbus, OH, United States; ^4^ Division of Big Data Science, Korea University Sejong Campus, Sejong, Republic of Korea; ^5^ Division of Statistics and Data Science, University of Cincinnati, Cincinnati, OH, United States

**Keywords:** genome-wide association studies, GWAS summary statistics, complex traits, genetic correlation, functional annotation

## Abstract

Genome-wide association studies (GWAS) have successfully identified a large number of genetic variants associated with traits and diseases. However, it still remains challenging to fully understand the functional mechanisms underlying many associated variants. This is especially the case when we are interested in variants shared across multiple phenotypes. To address this challenge, we propose graph-GPA 2.0 (GGPA 2.0), a statistical framework to integrate GWAS datasets for multiple phenotypes and incorporate functional annotations within a unified framework. Our simulation studies showed that incorporating functional annotation data using GGPA 2.0 not only improves the detection of disease-associated variants, but also provides a more accurate estimation of relationships among diseases. Next, we analyzed five autoimmune diseases and five psychiatric disorders with the functional annotations derived from GenoSkyline and GenoSkyline-Plus, along with the prior disease graph generated by biomedical literature mining. For autoimmune diseases, GGPA 2.0 identified enrichment for blood-related epigenetic marks, especially B cells and regulatory T cells, across multiple diseases. Psychiatric disorders were enriched for brain-related epigenetic marks, especially the prefrontal cortex and the inferior temporal lobe for bipolar disorder and schizophrenia, respectively. In addition, the pleiotropy between bipolar disorder and schizophrenia was also detected. Finally, we found that GGPA 2.0 is robust to the use of irrelevant and/or incorrect functional annotations. These results demonstrate that GGPA 2.0 can be a powerful tool to identify genetic variants associated with each phenotype or those shared across multiple phenotypes, while also promoting an understanding of functional mechanisms underlying the associated variants.

## 1 Introduction

Genome-wide association studies (GWAS) have identified hundreds of thousands of genetic variants significantly associated with human traits and diseases (Buniello et al., 2019). Despite the great success of GWAS, multiple challenges still remain to be addressed. First, the single-trait analysis commonly used in GWAS can suffer from weak statistical power to detect risk variants. Pleiotropy, which refers to the phenomenon of a single genetic variant affecting multiple traits, has been reported to widely exist in human genome (Sivakumaran et al., 2011). For example, previous studies reported high genetic correlation between schizophrenia (SCZ) and bipolar disorders (BIP) (Cross-Disorder Group of the Psychiatric Genomics Consortium and others, 2013a; Cross-Disorder Group of the Psychiatric Genomics Consortium and others, 2013b). Integrative analysis combining GWAS data of multiple genetically related phenotypes has been proven to be a powerful approach to improve statistical power to detect risk variants by leveraging pleiotropy (Chung et al., 2014; Li et al., 2014; Chung et al., 2017). Second, our understanding of the functional mechanisms underlying many risk variants is still limited. It was reported that about 90% of the genome-wide significant hits in published GWAS are located in non-coding regions and we still have limited understanding of their functional impacts on human complex traits (Hindorff et al., 2009). By considering that functional roles relevant to genetic variants may affect the corresponding distribution in the GWAS summary statistics, incorporating functional annotations can help improve understanding of functional mechanisms by which risk variants may affect phenotypes. For example, it was reported that single nucleotide polymorphisms (SNPs) associated with psychiatric disorders such as BIP or SCZ are more likely to be associated with the central nervous system or brain function (Hoseth et al., 2018; Shahab et al., 2019).

Multiple statistical and computational approaches have been proposed to leverage pleiotropy and integrate functional annotations to improve association mapping. Here we focus on approaches based on GWAS summary statistics considering their wide availability, unlike the original phenotype and genotype data that are often burdensome and time-consuming to obtain. The first group of approaches focuses only on integrating multiple GWAS datasets. Multiple methods have been developed based on association testing, which usually generate their test statistics under the null hypothesis of significant association. An early example is TATES (Van der Sluis et al., 2013) which combines *p*-values of each single-trait analysis to generate one comprehensive *p*-value by applying eigen-decomposition to the correlation matrix of *p*-values. In recent years, MTAG has been a popular method for conducting meta-analysis of GWAS summary statistics for different traits, and it has been reported that it is robust to sample overlap (Turley et al., 2018). It constructs a generalized method of moments estimator using the estimated effect size of each trait.

The second group of approaches focuses only on integrating functional annotations. The first subgroup of methods in this direction is based on false discovery rate (FDR) approaches. An early example is the stratified FDR (sFDR) method (Schork et al., 2013), which evaluates enrichment with respect to functional annotations using stratified Q-Q plots and determines their statistical significance using Kolmogorov-Smirnov test. Similarly, the covariate-modulated local FDR (cmfdr) (Zablocki et al., 2014) incorporates functional annotations as prior distribution for non-null group. The second subgroup of methods is based on heritability estimation. The stratified linkage disequilibrium (LD) score regression (LDSC) (Finucane et al., 2015; Finucane et al., 2018) and GCTA (Yang et al., 2011) are popular approaches in this direction and these approaches are based on the idea of heritability partitioning based on functional annotations. Later, SumHer (Speed and Balding, 2019) further improved LDSC by relaxing its assumptions, e.g., those related to minor allele frequencies (MAF) and confounding bias. The third subgroup of methods in this direction is based on Bayesian approaches. In these approaches, GWAS data is often considered as emission distributions while functional annotations are used as prior knowledge to guide latent association status. fGWAS (Pickrell, 2014) models the latent association status on functional annotations, focusing on binary annotations. GenoWAP (Lu et al., 2016b) considers two different latent components, one for disease-specific functionality (specific to GWAS) and another for general functionality, and integrates functional annotation as prior knowledge affecting general functionality. LSMM Ming et al. (2018) integrates functional annotations with GWAS data by using a latent sparse mixed model. Specifically, a mixed model is considered to model the latent association status on functional annotations using both fixed and random effects, while a spike-slab prior is used for variable selection of functional annotations. There are also other approaches to integrate functional annotations, e.g., using regression models. For example, GARFIELD (Iotchkova et al., 2019) first identifies links between SNPs and functional annotations based on their overlap considering LD. Then, statistical significance of these links are determined using a logistic regression of GWAS signals on functional annotations. RolyPoly (Calderon et al., 2017) uses a regression approach to model relationships between SNP effect sizes and functional annotations. GoShifter (Trynka and Raychaudhuri, 2013) evaluates enrichment by shifting locations of functional annotations, which makes it less sensitive to biases arising from local genomic structure.

The third group of approaches aims to achieve the best of both worlds by integrating multiple GWAS datasets along with functional annotations. GPA (Chung et al., 2014) is a pioneer in this direction. GPA uses a hierarchical modeling approach to incorporate multiple GWAS datasets and functional annotations within a unified framework. EPS (Liu et al., 2016) later improved GPA by allowing more diverse types of functional annotations and addressing LD. However, it was still limited in the sense of the number of phenotypes, as in the case of GPA. LPM (Ming et al., 2020) improved these approaches by allowing to integrate a larger number of phenotypes using latent probit models. For more comprehensive review of the statistical methods for leveraging pleiotropy and incorporating functional annotations, please check Hackinger and Zeggini (Hackinger and Zeggini, 2017) and Cano-Gamez and Trynka (Cano-Gamez and Trynka, 2020), respectively.

For the purpose of multi-disease analysis, we previously proposed graph-GPA (GGPA), a Bayesian approach that models a pleiotropic architecture using a latent Markov random field (MRF) approach indicating phenotype-genotype associations (Chung et al., 2017). First, the pleiotropic architecture is represented as a phenotype graph, where each node corresponds to a phenotype and an edge between two phenotypes represents the genetic correlation between them. This phenotype graph representation is a unique feature of GGPA. It not only allows integration of a large number of phenotypes, but also provides more intuitive representation about genetic relationships among phenotypes, compared to other approaches. Second, GGPA can simultaneously detect significant SNPs and identify genetic relationships among phenotypes in a rigorous manner within a unified framework. This is another advantage of GGPA over multi-step approaches because it allows more effective information sharing and more accurate reflection of uncertainties between different inferences. Third, the Bayesian framework of GGPA provides flexibility and allows incorporating various types of biological or expert knowledge as prior distribution. For example, GGPA was later further extended by allowing to incorporate prior knowledge on the phenotype graph architecture generated from text mining of biomedical literature (Kim et al., 2018).

In spite of such strengths and flexibility, unfortunately, the previous version of GGPA did not allow incorporating functional annotations. This was an important limitation given the potential of functional annotations to further improve genetic analysis. Incorporating functional annotations can not only potentially improve understanding of functional mechanisms underlying identified genetic variants, but also lead to more reliable and meaningful findings of genetic variants themselves (Lu et al., 2016a; Lu et al., 2017). In order to address this critical limitation, in this paper, we propose GGPA 2.0, an extension of GGPA that allows to incorporate functional annotations and to integrate GWAS datasets for multiple phenotypes within a unified framework. Specifically, GGPA 2.0 improves statistical power to detect associated genetic variants (both those associated with each trait and multiple traits) and inference of genetic relationships among phenotypes, by incorporating functional annotations in addition to GWAS datasets for multiple phenotypes. In addition, it also provides information about relevance of each functional annotation for the phenotype of interest, which allows further investigation of relevant tissues and/or cell types related to genetic basis of diseases.

## 2 Methods

### 2.1 Overview of GGPA 1.0

GGPA takes GWAS summary statistics (genotype-phenotype association *p*-values) for SNP *t* and phenotype *i*, denoted as *p*_*it*_, as input, where *i* = 1, … , *n* and *t* = 1, … , *T*. For convenience, in modeling and visualization, we transform *p*_*it*_ as *y*_*it*_ = Φ^−1^ (1 − *p*_*it*_), where Φ is the cumulative distribution of the standard normal variable. We model the density of *y*_*it*_ with the latent association indicator *e*_*it*_ using a lognormal-normal mixture:

$$p\left(y_{it}|e_{it},\mu_{i},\sigma_{i}^{2}\right)=e_{it}\text{LN}\left(y_{it};\mu_{i},\sigma_{i}^{2}\right)+\left(1-e_{it}\right)\text{N}\left(y_{it};0,1\right),$$ (1)

where *e*_*it*_ = 1 if SNP *t* is associated with phenotype *i* and *e*_*it*_ = 0 otherwise, and LN and N denote the lognormal density and the normal density, respectively. For *y*_*it*_ corresponding to the associated SNPs (*e*_*it*_ = 1), we assume the lognormal distribution because the *p*-values of those SNPs are very likely to be less than 0.5 leading to *y*_*it*_ greater than zero (Chung et al., 2017).

To model genetic relationships among *n* phenotypes, we adopt a graphical model based on the MRF framework. Let ***G*** = (***V***, ***E***) denote an MRF graph with nodes ***V*** = (*v*_1_, … , *v*_*n*_) and edges ***E*** = {*E* (*i*, *j*): *i*, *j* = 1, … , *n*}. We can interpret *v*_*i*_ as phenotype *i* and *E* (*i*, *j*) = 1 means that phenotypes *i* and *j* are conditionally dependent (i.e., genetically correlated). Specifically, we model the latent association indicators of SNP *t*, ***e***_*t*_ = (*e*_1*t*_, … , *e*_*nt*_), and the graph structure with an auto-logistic scheme. The probability mass function for ***e***_*t*_ is given by

$$p\left(e_{t}|\alpha ,\beta ,G\right)=\exp\left(\overset{n}{\underset{i=1}{\sum }}\alpha_{i}e_{it}+\underset{i∼j}{\sum }\beta_{ij}e_{it}e_{jt}\right)/C\left(\alpha ,\beta ,G\right)$$ (2)

with the non-ignorable normalizing constant in the denominator given by

$$C\left(\alpha ,\beta ,G\right)=\underset{e^{*}\in E^{*}}{\sum }\exp\left(\overset{n}{\underset{i=1}{\sum }}\alpha_{i}e_{i}^{*}+\underset{i∼j}{\sum }\beta_{ij}e_{i}^{*}e_{j}^{*}\right),$$

where *α*_*i*_ is the MRF coefficient for the phenotype *i* such that larger values represent stronger SNP-phenotype associations, *β*_*ij*_ is the MRF coefficient for the pair of phenotypes *i* and *j* such that larger values represent stronger associations between the phenotypes, the symbol *i* ∼ *j* denotes that *v*_*i*_ is adjacent to *v*_*j*_, i.e., *E*(*i*, *j*) = 1, and $E^{*}$ is the set of all possible values of $e^{*}=\left(e_{1}^{*},\ldots ,e_{n}^{*}\right)$ .

The phenotype graph ***G*** is one of our key inferential targets in this framework. In our previous work, we found that MRF coefficient estimation can be biased when signals are weak in GWAS data and we showed that incorporating prior information for ***G*** can help address this issue and improve stability of the phenotype graph estimation (Kim et al., 2018). Specifically, we implemented text mining of biomedical literature to identify prior phenotype graph estimation, which we found to give biologically meaningful prior knowledge.

For the log-normal density in Eq. 1, we introduce the conjugate prior distribution:

$$\mu_{i}∼\text{N}\left(\theta_{\mu },\tau_{\mu }^{2}\right),\;\sigma_{i}^{2}∼\text{IG}\left(a_{\sigma },b_{\sigma }\right),$$

where IG(*a*, *b*) denotes the inverse gamma distribution with the shape parameter *a* and the rate parameter *b*. For the MRF coefficients in Eq. 2, we assume the following prior distributions:

$$\alpha_{i}∼\text{N}\left(\theta_{\alpha },\tau_{\alpha }^{2}\right),\;\beta_{ij}∼E\left(i,j\right)\;\Gamma \left(\beta_{ij};a_{\beta },b_{\beta }\right)+\left\{1-E\left(i,j\right)\right\}\;\delta_{0}\left(\beta_{ij}\right),$$

where Γ(*a*, *b*) denotes the gamma distribution with the shape parameter *a* and the rate parameter b, and *δ*_0_ denotes the Dirac delta function. Weakly informative priors are used for the top level of the Bayesian hierarchical model with the hyperparameters: *θ*_*μ*_ = 0, $\tau_{\mu }^{2}=10000$ , *θ*_*α*_ = 0, $\tau_{\alpha }^{2}=10000$ and *a*_*σ*_ = *b*_*σ*_ = 0.5. We use *a*_*β*_ = 4 and *b*_*β*_ = 2 so that most of *β*_*ij*_’s with *E*(*i*, *j*) = 1 are *a priori* distinct from zero.

The posterior inference is made using the Markov chain Monte Carlo (MCMC). First, we can make an inference about the genetic correlation among phenotypes by using both the estimated phenotype graph structure and the MRF coefficient estimates. Specifically, the phenotype graph ***G*** represents genetic relationship among phenotypes, where the posterior probability for each edge *p*(*E*(*i*, *j*)| ***Y***) indicates the probability that two phenotypes *i* and *j* are genetically correlated with each other, where ***Y*** indicates the GWAS dataset, i.e., the set of *y*_*it*_, *i* = 1, … , *n*, *t* = 1, … , *T*. In addition, the posterior samples of *β*_*ij*_ can be interpreted as a relative metric to gauge the degree of correlation between phenotypes *i* and *j*. Based on this rationale, we conclude that phenotype *i* and *j* are correlated if *p*(*E*(*i*, *j*)| ***Y***) > 0.5 and *p*(*β*_*ij*_ > 0| ***Y***) > 0.95. Second, association mapping of a single SNP with a specific phenotype is implemented based on *p*(*e*_*it*_ = 1| ***Y***), i.e., the posterior probability that SNP *t* is associated with phenotype *i*. Likewise, pleiotropic variants can be detected using *p*(*e*_*it*_ = 1, *e*_*jt*_ = 1| ***Y***) representing the posterior probability that SNP *t* is associated with both phenotypes *i* and *j*. Identification of pleiotropic variants for more than two phenotypes can be implemented in similar ways. Global FDR were controlled using the direct posterior probability approach (Newton et al., 2004).

### 2.2 Improvements in GGPA 2.0

In GGPA 2.0, in addition to the GWAS summary statistics, we also consider functional annotations ***a***_*t*_ = (*a*_1*t*_, … , *a*_*Mt*_), a vector of length *M*, for SNP *t*. Here we mainly focus on the binary annotations, i.e., *a*_*mt*_ = 1 if *t*th SNP is annotated in the *m*th (1 ≤ *m* ≤ *M*) functional annotation data. In GGPA 2.0, we incorporate the functional annotation as a modifier for the MRF intercept so that when the *t*th SNP is annotated in more functional annotation data, it can have a higher probability to be associated with phenotypes. Specifically, we modify Eq. 2 as follows:

$$p\left(e_{t}|\alpha ,\gamma ,\beta ,G,a_{t}\right)=\exp\left(\overset{n}{\underset{i=1}{\sum }}\left(\alpha_{i}+\overset{M}{\underset{m=1}{\sum }}\gamma_{im}a_{mt}\right)e_{it}+\underset{i∼j}{\sum }\beta_{ij}e_{it}e_{jt}\right)/C\left(\alpha ,\gamma ,\beta ,G,a_{t}\right)$$ (3)

with the non-ignorable normalizing constant in the denominator given by

$$C\left(\alpha ,\gamma ,\beta ,G,a_{t}\right)=\underset{e^{*}\in E^{*}}{\sum }\exp\left(\overset{n}{\underset{i=1}{\sum }}\left(\alpha_{i}+\overset{M}{\underset{m=1}{\sum }}\gamma_{im}a_{mt}\right)e_{i}^{*}+\underset{i∼j}{\sum }\beta_{ij}e_{i}^{*}e_{j}^{*}\right),$$

where *γ*_*im*_ (>0) is the MRF coefficient for importance of annotation *m* for phenotype *i* such that larger values represent richer enrichment of tissues or cells in phenotypes. Note that here we assume *γ*_*im*_ > 0 so that associations of genetic variants with phenotypes are supported, rather than penalized, by being annotated.

The functional annotation coefficient *γ*_*im*_ has the following hyperpriors:

$$\begin{array}{cc} \gamma_{im} & ∼u_{im}\Gamma \left(\gamma_{im};a_{\gamma },b_{\gamma }\right)+\left(1-u_{im}\right)\delta_{0}\left(\gamma_{im}\right),\\ u_{im} & ∼\text{Ber}\left(p_{u}\right),p_{u}∼\text{Unif}\left(0,1\right)=\text{Beta}\left(1,1\right), \end{array}$$

where Ber(*p*) denotes the Bernoulli distribution with success probability *p*, Unif(*l*, *u*) denotes the uniform distribution with lower and upper limits *l* and *u*, and Beta(*a*, *b*) denotes the beta distribution with two shape parameters, *a* and *b*. We use *a*_*γ*_ = 4 and *b*_*γ*_ = 2. Given this model, the posterior inference is made using MCMC. Specifically, we implement a Metropolis-within-Gibbs algorithm whose full details are provided in Supplementary Section 1. The genetic correlation among phenotypes can be inferred and the association mapping can be implemented as described in the previous section. We note that although we use the same set of parameters for these purposes, their inference results will be different from GGPA 1.0 because incorporation of functional annotation data affects estimation of these parameters. Moreover, relevance of functional annotations with disease-risk-associated variants can be inferred using *γ*_*im*_ representing the importance of functional annotation *m* for phenotype *i*. Specifically, we declare that annotation *m* is associated with phenotype *i* if *γ*_*im*_ is significantly different from zero, e.g., *p*(*γ*_*im*_ > 0| ***Y***) > 0.95. Based on significantly nonzero *γ*s, we can identify cells or tissues that are enriched in the corresponding phenotypes. Again the direct posterior probability approach (Newton et al., 2004) is used to control global FDR.

### 2.3 Simulation setting

**FIGURE 1:**
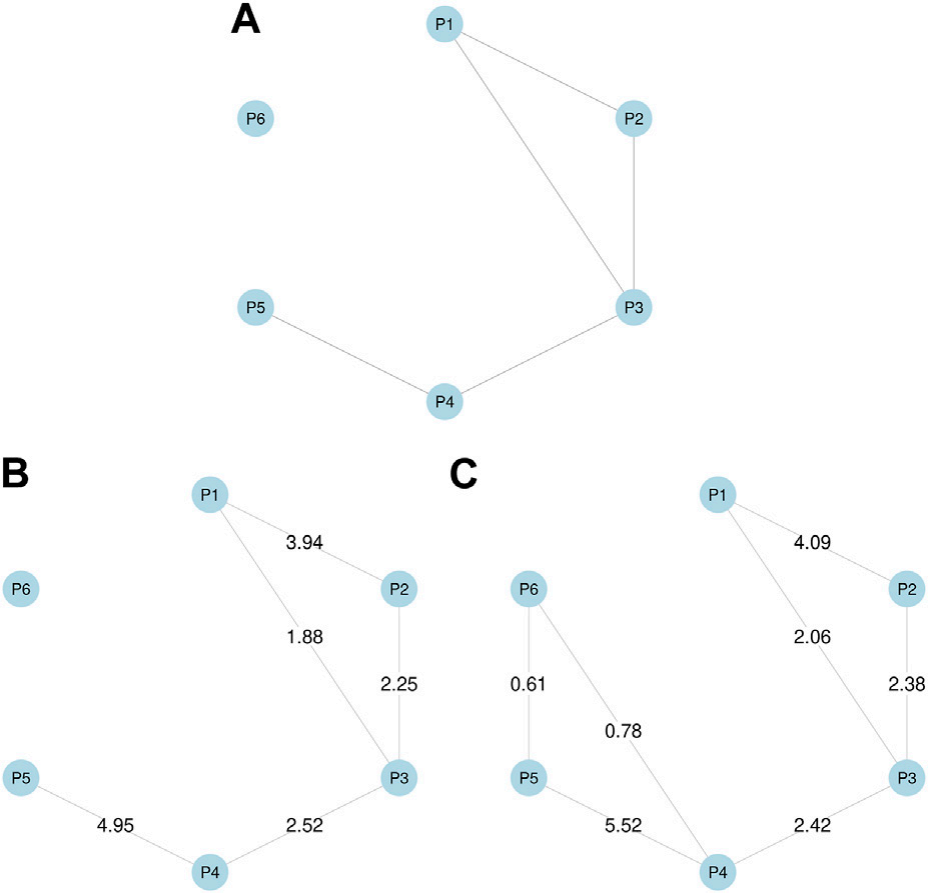
Simulation studies. **(A)** True phenotype graph used to generate simulated data. **(B)** Phenotype graph estimated using annotations, which is identical to the true graph. **(C)** Phenotype graph estimated without using annotations, which added two additional edges between P4 and P6, and between P5 and P6. Values on edges show *β* coefficient estimates.

For the simulation study, we generated the simulated data using the following steps. First, we assumed the true phenotype graph depicted in Figure 1A for phenotype *P*1, … , *P*6, with the MRF coefficients (*α*_1_, *α*_2_, *α*_3_, *α*_4_, *α*_5_, *α*_6_) = (−4.7, −3.0, −5.5, −4.8, −3.6, −2.5) and (*β*_12_, *β*_13_, *β*_23_, *β*_34_, *β*_45_) = (4.0, 1.8, 2.3, 2.5, 5.0), while all the remaining *β*_*ij*_ were set to zeros. Second, assuming *T* = 200, 000 SNPs and *M* = 5 annotations, we generated each binary vector ***a***_*m*_, of which elements are set to one for 10% SNPs. We assumed *γ*_11_ = *γ*_21_ = *γ*_31_ = 1 and *γ*_42_ = *γ*_52_ = *γ*_62_ = 2, while all the remaining *γ*_*im*_ were set to zeros. We also considered two other settings for *γ*s whose results are provided in Supplementary Section 2. Third, we generated ***e***_*t*_ by running the Gibbs sampler for 1,000 iterations based on Eq. 2. Finally, we generated *y*_*it*_ using Eq. 1, where ***μ*** = (1.05, 0.9, 1.0, 1.0, 1.05, 0.95) and ***σ*** = (0.4, 0.3, 0.35, 0.3, 0.45, 0.4).

### 2.4 GWAS datasets and functional annotations used in the real data analysis

Here we analyzed GWAS data for two sets of diseases to demonstrate the usefulness of GGPA 2.0. The first set consists of five autoimmune diseases, including systemic lupus erythematosus (SLE), ulcerative colitis (UC), Crohn’s disease (CD), rheumatoid arthritis (RA), and type I diabetes (T1D). The second set consists of five psychiatric disorders, including attention deficit-hyperactivity disorder (ADHD), autism spectrum disorder (ASD), major depressive disorder (MDD), bipolar disorder (BIP), and schizophrenia (SCZ). Summary statistics for ten different disease types were downloaded from the GWAS Catalog: SLE (Langefeld et al., 2017), RA (Okada et al., 2014), UC (De Lange et al., 2017), CD (De Lange et al., 2017), T1D (Bradfield et al., 2011), ADHD (Lee et al., 2019), ASD (Lee et al., 2019), BIP (Lee et al., 2019), SC) (Lee et al., 2019), and MDD (Lee et al., 2019). We considered two sets of functional annotations based on GenoSkyline (Lu et al., 2016a) or GenoSkyline-Plus (Lu et al., 2017) respectively. GenoSkyline is a tissue-specific functional prediction generated with integrated analysis of epigenomic annotation data. It calculates the posterior probability of being functional which is referred to as GenoSkyline score. We used Genoskyline scores for 7 tissue types: brain, gastrointestinal tract (GI), lung, heart, blood, muscle, and epithelium. Specifically, to generate the binary annotations, we set *a*_*mt*_ = 1 if the corresponding GenoSkyline score is above 0.5. GenoSkyline-Plus is a comprehensive update of GenoSkyline by incorporating RNA-seq and DNA methylation data into the framework and extending to 127 integrated annotation tracks, covering a spectrum of human tissue and cell types. Similarly, we generated the binary annotations using the same cutoff at 0.5. We considered 1,919,526 SNPs that are shared among these GWAS datasets. We further removed SNPs with missing values and kept one SNP in every 10 SNPs to reduce dependent SNPs, leading to 187,335 SNPs. As a reference, after subsampling every tenth SNP, the average *r* ^2^ of the nearest pair drops notably from 0.48 to 0.36, as calculated using the R package “LDlinkR” and 10,000 randomly chosen pairs. Our approach involves conducting statistical inference by incorporating functional annotations, accounting for the correlation among *p*-values, as covariates. In general, identifying the source of variability can lessen conditional correlations between responses. In other words, including the variables responsible for the correlation in the model can lead to a lower correlation. For example, if *X* contains all factors that explain the correlation of the *Y* vector, the elements of *Y* are (conditionally) independent given *X*, which is the foundation of the regression model. Additionally, recognizing the source of dependence can improve statistical inference. This viewpoint suggests that our approach can be effective without negatively impacting FDR control. Consequently, both the marginal correlation reduction between SNPs resulting from our sampling strategy and the conditional correlation reduction achieved by incorporating functional annotation as covariates are simultaneously implemented, expected to significantly reduce the violation of model assumptions and substantially decrease the infringement on FDR control.

### 2.5 Adjusting for sample overlap

Integrating GWAS summary statistics across multiple phenotypes can be affected by the potential overlap of subjects among those studies, making data sets dependent. As a consequence, the effects of pleiotropy can be confounded with the spurious effects caused by sample overlap. To address the potential sample overlap issue, we decorrelated the GWAS summary statistics (LeBlanc et al., 2018) before applying the proposed methods. Specifically, after we obtained *y*_*it*_ as described in Section 2.1, we decorrelated them by ***Y***_*decorr*_ = ***C*** ^−1/2^ ***Y***, where ***C*** is the sample correlation matrix of ***Y***, and ***Y*** is the observed matrix of which element is *y*_*it*_. It has been reported that the resultant ***Y***_*decorr*_ is less biased by the sample overlap for the genetic correlation inference, compared to the case of using the original ***Y*** (LeBlanc et al., 2018). For autoimmune diseases, we decorrelated UC and CD. In the case of five psychiatric disorders, we decorrelated all of them together, by considering the overlap pattern of subjects between cohorts.

## 3 Results

### 3.1 Simulation studies

Here we especially focused on comparing the GGPA models with incorporating functional annotations to one without the functional annotations. Across the simulation settings (Supplementary Section S2), we did not recognize any notable issues regarding the convergence of the proposed MCMC sampler (Supplementary Figures S1, S8, S16) and global FDR is well controlled at the nominal level for a wide range of FDR values (Supplementary Figures S5, S12, S20). Interestingly, we observe that parameter estimation accuracy was improved by incorporating annotations (Supplementary Figures S3, S4, S10, S11, S18, S19). Specifically, when functional annotations were incorporated, the point estimates were closer to true values for all parameters, and the corresponding 95% credible intervals always covered the true values. In contrast, when functional annotations were not incorporated, the parameter estimates were less accurate and the true values were often outside the 95% credible intervals. The result shows that incorporating information from functional annotations leads to better parameter estimation. Next, we evaluated the impact of functional annotations on the estimation of genetic relationships among phenotypes. Figures 1B, C show the phenotype graphs estimated with and without annotations respectively. We can observe that the true phenotype graph can be more accurately estimated by incorporating annotations. Specifically, if we ignore functional annotations, P6 is falsely connected to P4 and P5 although P6 is designed not to be correlated with any other phenotypes. This result shows that if SNPs are truly associated with functional annotations, the analysis ignoring the functional annotations can lead to inaccurate estimation of genetic relationships among phenotypes. Finally, we evaluated the association mapping results. We found that incorporating annotations generally leads to larger numbers of associated SNPs (Supplementary Tables S3, S4) and identifying more truly associated SNPs compared to the case that we ignored functional annotations (Supplementary Figures S14). These results suggest that incorporating functional annotations can improve association mapping as well. In summary, the simulation studies show that i) incorporating functional annotations improves the accuracy of parameter estimation and the power of detecting associated SNPs; and ii) ignoring functional annotations can result in misleading conclusions about relationships among phenotypes when functional annotations are truly related to the associated SNPs.

### 3.2 Real data analysis

#### 3.2.1 Applications to autoimmune diseases

**FIGURE 2:**
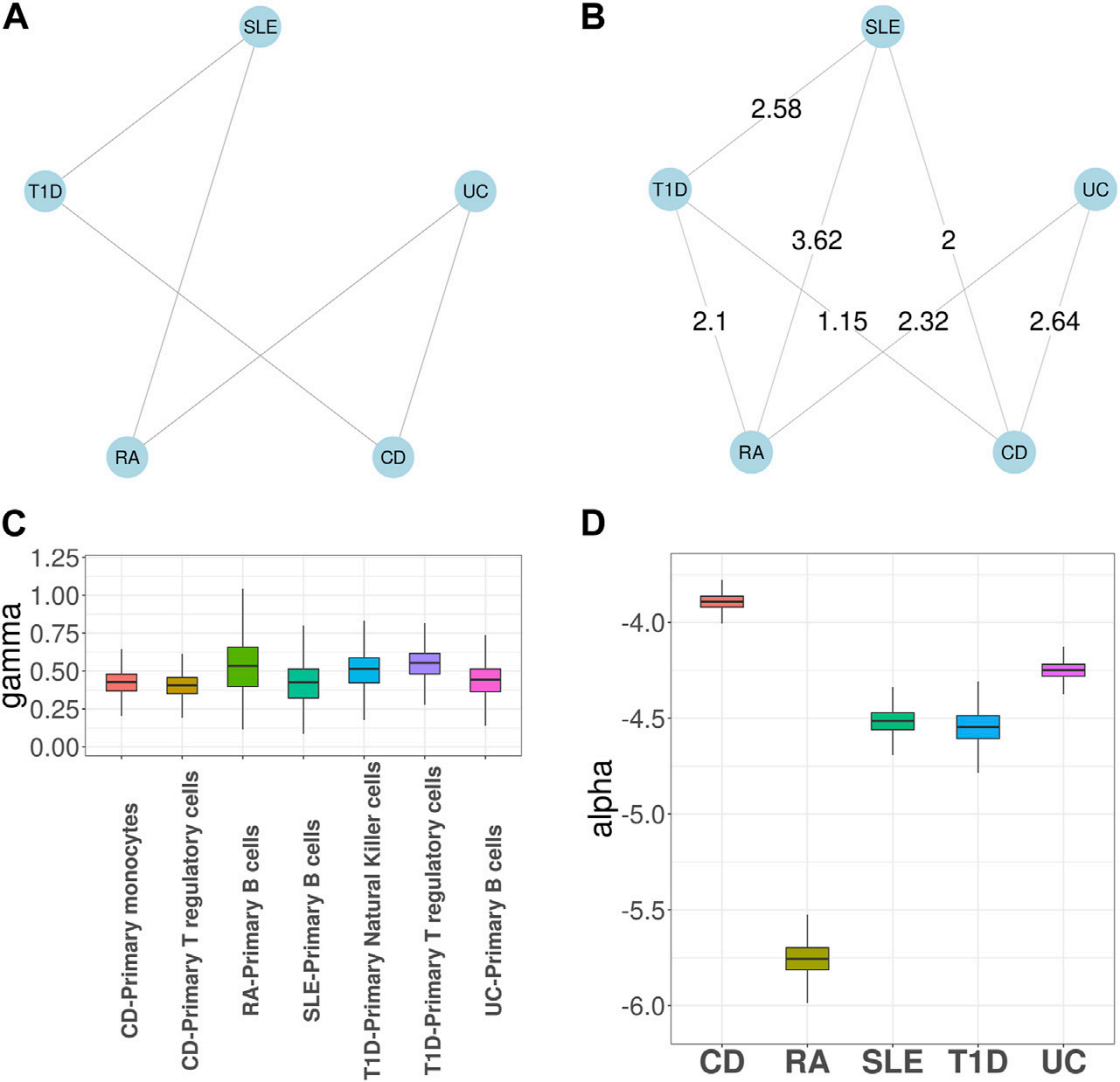
GGPA 2.0 analysis of autoimmune diseases, including systemic lupus erythematosus (SLE), rheumatoid arthritis (RA), ulcerative colitis (UC), Crohn’s disease (CD), and type 1 diabetes (T1D), using annotations of Genoskyline-Plus. **(A)** Prior phenotype graph obtained by biomedical literature mining. **(B)** Estimated phenotype graph, where values on the edges show *β* coefficient estimates. **(C)** Coefficient estimates of *γ* show that B cells and regulatory T cells are associated with these autoimmune diseases. **(D)** Coefficient estimates of *α* suggest a stronger genetic basis of CD compared with other autoimmune diseases.

We first applied GGPA 2.0 to analyze the five autoimmune diseases, along with seven tissue-specific GenoSkyline annotations, including blood, brain, epithelium, Gastrointestinal tract (GI), heart, lung, and muscle. Figure 2A shows the prior graph for these five diseases, which was derived from biomedical literature mining (Kim et al., 2018). It illustrates links between SLE and T1D, SLE and RA, UC and CD, UC and RA, and CD and T1D, respectively. Supplementary Figure S29 shows the estimated phenotype graph (Supplementary Figure S26 shows MRF coefficients *β*s) and it indicates that 7 pairs out of 10 have edges, suggesting extensive pleiotropy among these diseases. Compared with the prior phenotype graph, GGPA 2.0 additionally detected the pleiotropies between RA and T1D, and between SLE and CD. These two pleiotropies have been reported in previous studies (Sanchez-Burson et al., 2004; Kim et al., 2018; Westra et al., 2018). We further applied LDSC (Finucane et al., 2015; Finucane et al., 2018) and LPM (Ming et al., 2020) to the same dataset to evaluate the phenotype graph estimated using GGPA 2.0 (Supplementary Tables S18–S20). We could observe that many edges in the disease graph obtained using GGPA 2.0 can also be found by LPM. In addition, some well-known pairs also ranked high in LDSC (e.g., CD-UC) although it was not trivial to prioritize genetically correlated pairs using LDSC because its correlation coefficients were overall comparable across all the pairs.

Supplementary Figure S28 shows *γ* coefficient estimates indicating importance of functional annotations for each disease. Blood was determined to be the key tissue for most of the autoimmune diseases, which is well supported by existing literature indicating the established relationships between blood and autoimmune diseases (Tyndall and Gratwohl, 1997; Olsen et al., 2004). In addition, epithelium and GI were also significantly associated with UC and CD, which is consistent with the fact that UC and CD are chronic inflammatory bowel diseases (Gohil and Carramusa, 2014). Finally, the estimates of *α* show that CD has the largest coefficient estimate, suggesting its strongest genetic basis (Supplementary Figure S27). As expected, in the association mapping (Supplementary Table S7), CD has the largest number of SNPs associated with it. We further applied MTAG (Turley et al., 2018) and LPM (Ming et al., 2020) to the same dataset to evaluate the association mapping provided by GGPA 2.0. In general, GGPA 2.0 usually identifies more risk SNPs compared to LPM and MTAG (Supplementary Table S24). We further checked overlap among the risk SNPs identified using GGPA 2.0, LPM, and MTAG (Supplementary Figures S75–S79). We found that GGPA 2.0 and LPM give comparable results in general and most of the risk SNPs identified using LPM could also be identified using GGPA 2.0. Interestingly, the risk SNPs identified using MTAG do not overlap much with GGPA2 and LPM.

Given the common importance of blood across the autoimmune diseases, we further investigated these diseases using the functional annotations based on 12 GenoSkyline-Plus tracks related to blood. Figure 2B shows the estimated phenotype graph, which shares the same set of edges as in the case that we used GenoSkyline annotations. Figure 2C shows the *γ* coefficient estimates for GenoSkyline-Plus tracks and only three tracks have nonzero coefficient estimates. Specifically, i) B cells were enriched for CD, RA, SLE, and UC; ii) regulatory T cells were enriched for CD and T1D; and iii) natural killer cells were enriched for T1D. These results are consistent with previous literature indicating connections between autoimmune disease and these immune cell types (Roep, 2003; Tsai et al., 2008; Nashi et al., 2010; Fraker and Bayer, 2016; Gardner and Fraker, 2021). Finally, in Figure 2D, we observed that CD still has the largest *α* coefficient estimate among the autoimmune diseases, leading to more SNPs significantly associated with it.

**FIGURE 3:**
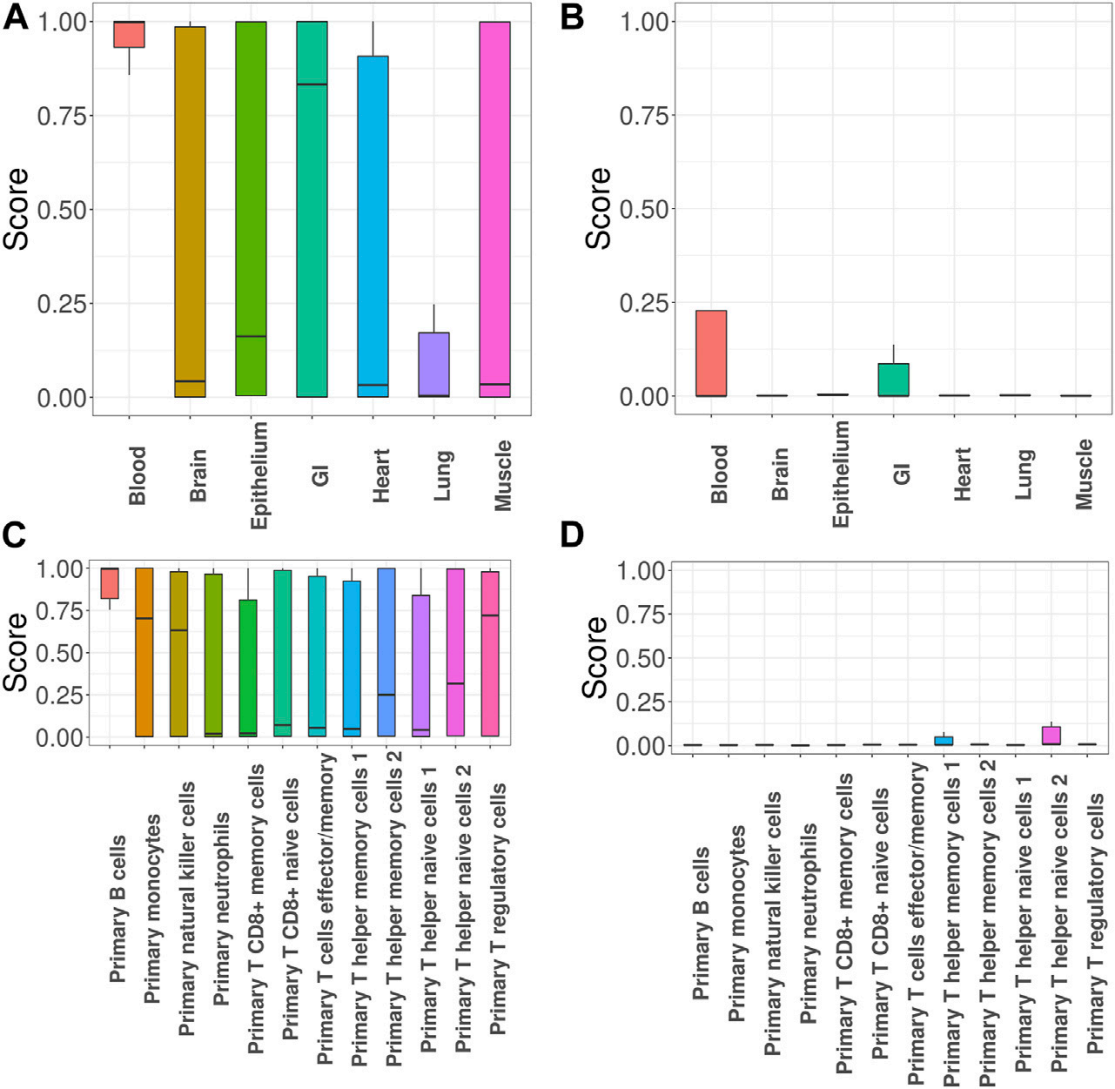
GGPA 2.0 analysis of systemic lupus erythematosus (SLE). **(A)** GenoSkyline scores of various tissues for the associated SNPs that were uniquely identified using functional annotations. **(B)** GenoSkyline scores from the analysis without using functional annotations. **(C)** GenoSkyline-Plus scores of various immune cell types for the associated SNPs that were uniquely identified using functional annotations. **(D)** GenoSkyline-Plus scores from the analysis without using functional annotations.

Next, we focused on investigation of SLE, the most common type of lupus and an autoimmune disease that causes inflammation and tissue damage in the affected organs. Here we specifically focused on evaluating the impact of incorporating functional annotations on the association mapping. For this purpose, we compared the functional importance of the SNPs that were uniquely identified with functional annotations (denoted as + SNPs) vs. those without (denoted as -SNPs). Figures 3A, B show the GenoSkyline scores of +SNPs and -SNPs, where a larger score suggests a larger likelihood to be functional in the corresponding tissue. The results indicate that + SNPs have overall significantly higher GenoSkyline scores compared to -SNPs. In addition, +SNPs were enriched for blood, which is consistent with our analyses above. They were followed by enrichment for GI and it has been reported that SLE may affect GI (Fawzy et al., 2016). Then, we implemented deeper investigation with functional annotations of GenoSkyline-Plus corresponding to blood, and compared the functional importance of the SNPs that were uniquely identified with functional annotations (denoted as + SNPs) to those without functional annotations (denoted as -SNPs). We observed the significant enrichment of +SNPs for B cells (Figure 3C), and the role of B cells in lupus pathogenesis was previously well described (Nashi et al., 2010). In contrast, -SNPs have extremely low GenoSkyline-Plus scores, and most of them were close to zeros (Figure 3D). These results indicate that ignoring functional annotations may lead to the identification of misleading SNPs that have no biological functions, while incorporating functional annotations can help identify functional SNPs and facilitate understanding of underlying biological mechanisms. To confirm this, we checked the results without using functional annotation (Supplementary Section S3.1.3 in Supplementary Materials) and the results indicate that incorporation of functional annotations leads to identification of more risk SNPs.

#### 3.2.2 Applications to psychiatric disorders

**FIGURE 4:**
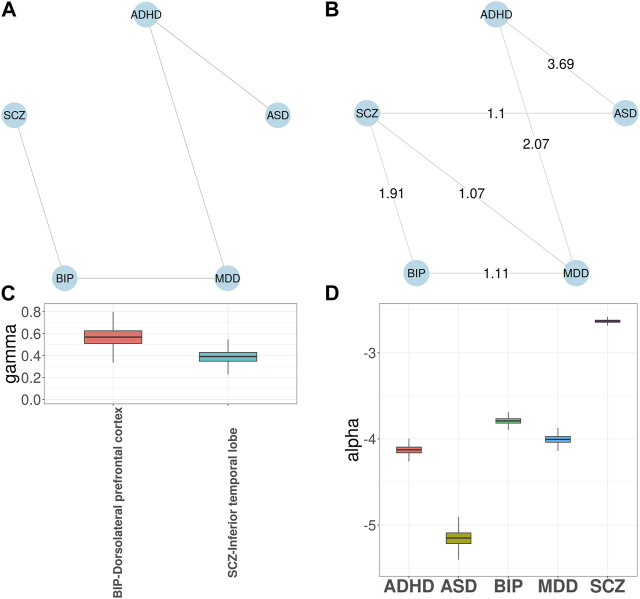
GGPA 2.0 analysis of five psychiatric disorders, including attention deficit-hyperactivity disorder (ADHD), autism spectrum disorder (ASD), major depressive disorder (MDD), bipolar disorder (BIP), and schizophrenia (SCZ), using annotations of GenoSkyline-Plus. **(A)** Prior phenotype graph obtained by biomedical literature mining. **(B)** Estimated phenotype graph, where values on the edges show *β* coefficient estimates. **(C)** Coefficient estimates of *γ* show that dorsolateral prefrontal cortex is associated with BIP and inferior temporal lobe is associated with SCZ. **(D)** Coefficient estimates of *α* suggest a stronger genetic basis of SCZ compared with other psychiatric disorders.

Next, we applied GGPA 2.0 to the five psychiatric disorders. The prior disease graph is shown in Figure 4A and indicates links between ASD and ADHD, ADHD and MDD, MDD and BIP, and BIP and SCZ, respectively. First, we implemented investigation using the functional annotations of GenoSkyline. Supplementary Figure S49 shows the estimated phenotype graph and three additional disorder pairs were identified, including ADHD-SCZ, ASD-SCZ, and MDD-SCZ. The connections between SCZ and the other three disorders have been previously reported (Canitano and Pallagrosi, 2017; Chen et al., 2017; Arican et al., 2019). Supplementary Figure S48 shows *γ* coefficient estimates and indicates that blood and brain tissues are significantly enriched for BIP and SCZ, respectively. Along with the natural connection between psychiatric disorders and brain (Notaras et al., 2015), aberrant blood levels of the cytokine network components has been reported for psychiatric disorders (Goldsmith et al., 2016), supporting the connection between BIP and blood. Again, given the natural connection between psychiatric disorders and brain, we implemented investigation using the eight brain-related GenoSkyline-Plus annotations to understand specificity of brain regions related to these psychiatric disorders. When this set of functional annotations were considered, the edge between ADHD and SCZ disappeared in the estimated phenotype graph (Figure 4B). Figure 4C shows that dorsolateral prefrontal cortex is significantly enriched for BIP while inferior temporal lobe is significantly enriched for SCZ. These enrichment are well supported by previous literature (Rajkowska et al., 2001; Liu et al., 2020). SCZ had the largest *α* coefficient and the largest number of SNPs were associated with SCZ in both cases (Figure 4D; Supplementary Figure S47; Supplementary Table S11).

**FIGURE 5:**
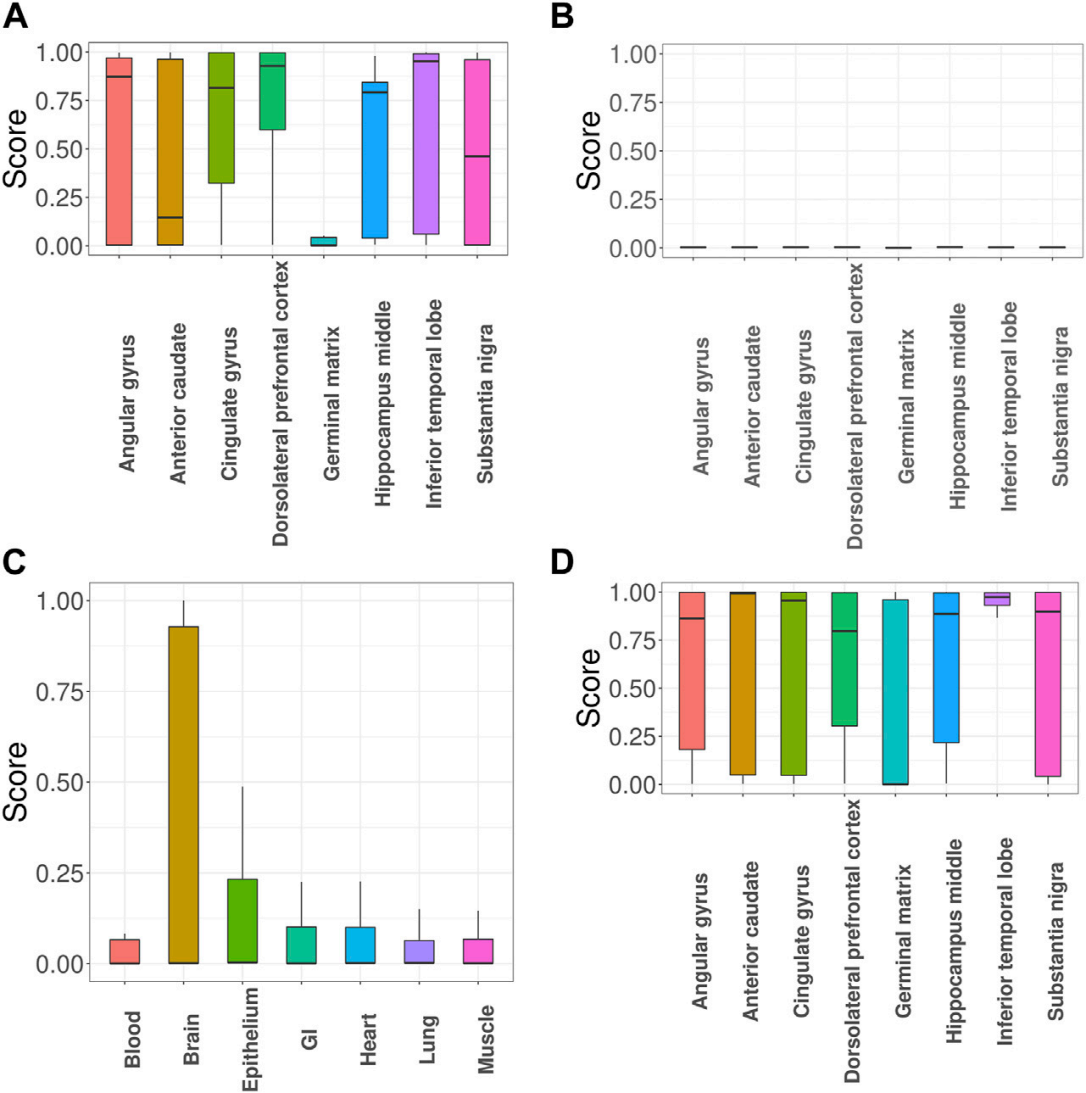
GGPA 2.0 analysis of major depressive disorder (MDD) and schizophrenia (SCZ). **(A)** GenoSkyline-Plus scores of various brain regions for the MDD-associated SNPs that were uniquely identified using functional annotations. **(B)** GenoSkyline-Plus scores from the analysis without using functional annotations. **(C)** GenoSkyline scores of various tissues and **(D)** GenoSkyline-Plus scores of various brain regions for the SCZ-associated SNPs that were uniquely identified using functional annotations.

Next, we evaluated impacts of incorporating functional annotations on the association mapping, focusing on MDD and SCZ. In Figure 5A, the SNPs identified using functional annotations have higher GenoSkyline scores for cingulate gyrus and dorsolateral prefrontal cortex. This observation is consistent with previous studies indicating that cell density, neuronal size, and signaling in these two brain regions do have an impact on MDD (Cotter et al., 2002; Tripp et al., 2012). In contrast, the scores of SNPs identified without using functional annotations are close to zeros (Figure 5B). Figure 5C shows the GenoSkyline scores for the SNPs identified using functional annotations, and we can observe higher scores for brain. In addition, Figure 5D shows enrichment of inferior temporal lobe for these SNPs, which is well supported by the relevance of this brain region with SCZ (Liu et al., 2020). In summary, GGPA might not only be powerful in detecting potentially functional SNPs, but also can potentially eliminate SNPs with irrelevant functions.

Finally, we applied GGPA 2.0 to investigate the pleiotropy between BIP and SCZ. We incorporated eight brain-related Genoskyline-Plus annotations and identified 242 SNPs significantly associated with both BIP and SCZ (Supplementary Table S12), which corresponds to 104 genes. According to the GWAS Catalog (Buniello et al., 2019), many of these genes have previously been reported to be associated with both BIP and SCZ, e.g., *PBRM1*, *MSRA*, and *BCL11B*. Compared to the analysis without using functional annotations, incorporating Genoskyline-Plus annotations uniquely identified 10 more genes, including *PMVK*, *TAOK2*, and *MAD1L1*, which have been reported to be associated with BIP and SCZ (Buniello et al., 2019). These results indicate that incorporating functional annotations can potentially improve statistical power to identify risk-associated genetic variants. We again checked the results without using functional annotation (Supplementary Section S3.2.3 in Supplementary Materials) and the results indicate that incorporation of functional annotations leads to identification of more risk SNPs.

#### 3.2.3 Investigation of the impacts of the use of irrelevant/incorrect functional annotations and the variations in minor allele frequencies on the performance of GGPA 2.0

In the previous sections, we showed the power of GGPA 2.0 in identify relevant functional annotations, which in turn leads to the improved association mapping performance. However, in practice, it is often not trivial to know *a priori* which functional annotations are relevant to the phenotype of interest. Hence, it is important to confirm that a statistical model is robust to the use of irrelevant and/or incorrect functional annotations. To investigate the robustness of GGPA 2.0, we generated irrelevant/incorrect functional annotations and evaluated their impacts on GGPA 2.0. Specifically, we shuffled functional annotations of GenoSkyline and GenoSkyline-Plus, and then repeated the analyses of the five autoimmune diseases in Section 3.2.1 using these shuffled annotations. The results show that using these irrelevant/incorrect functional annotations have essentially no influence on the estimates of *β* (Supplementary Figures S63, S66) and *α* (Supplementary Figures S64, S67). Moreover, all estimates of *γ* were close to zero for these annotations (Supplementary Figures S65, S68), suggesting that GGPA 2.0 could recognize irrelevance of these annotations and prevent them affect the model fitting. Finally, in the sense of association mapping, we found that the numbers of significant SNPs essentially remain the same as those identified without using any functional annotations (Supplementary Tables S16, S17). In conclusion, we confirmed that GGPA 2.0 is robust to the use of irrelevant/incorrect functional annotations in the sense of parameter estimation, selection of functional annotations, and association mapping.

MAF of SNPs can be another potential factor that can affect the performance of GGPA 2.0. For example, Speed and colleagues investigated this issue and showed relevance of MAFs with heritability and functional enrichment (Speed et al., 2017; Gazal et al., 2018). Therefore, considering MAF of SNPs might help improve the performance of GGPA 2.0. Following a similar strategy used by Gazal and colleagues (Gazal et al., 2018), we incorporated MAF as one of the functional annotations by assigning ones to the SNPs with MAF less than 0.05, and zeros to the remaining SNPs. Then, we repeated the analyses implemented in Supplementary Sections S3.2.1, S3.2.2 using both GenoSkyline and this MAF vector as functional annotations. First, we analyzed the five autoimmune diseases with Genoskyline annotations as described in Supplementary Section S3.2.1, but with MAF as additional annotation. We found that incorporating MAF had a minimal impact on the estimates of *α* and *β* (Supplementary Figures S69, S70). However, we observed some changes in the estimates of *γ* although overall patterns remained similar. For example, CD-Epithelium and UC-Blood, which previously showed weak enrichment, were shrunken to zeros while the enrichment for UC-Epithelium became rather stronger (Supplementary Figure S71). Next, we analyzed the five psychiatric disorders in a similar way. However, in this case, we did not observe any significant changes (Supplementary Figures S72–S74). In summary, considering MAF seems to have some potential to improve the performance of GGPA 2.0 but more careful and thorough studies will be needed to have more concrete conclusions.

## 4 Discussion

In this paper, we proposed GGPA 2.0, which allows to integrate functional annotations with GWAS datasets for multiple phenotypes within a unified framework. Our simulation studies show that GGPA 2.0 can improve both the phenotype graph estimation and the association mapping by incorporating functional annotations. In real data applications, we applied GGPA 2.0 to five autoimmune diseases and five psychiatric disorders. The results indicate that the incorporation of functional annotation data not only leads to identification of novel risk SNPs, but also eliminates the SNPs with potentially less biological relevance. Finally, we found that GGPA 2.0 is robust to the use of irrelevant and/or incorrect functional annotations that we can often have in practice.

In spite of such exciting improvements, there are still some limitations to be addressed. First, the computational efficiency needs to be further improved. Specifically, the computation time increases as the number of phenotypes and functional annotations increases (Supplementary Section S3.3 in Supplementary Materials). Thus, it will be of great interest to investigate approaches that can improve computational efficiency, e.g., approximation approaches and parallel computing techniques. Second, because GGPA 2.0 uses *p*-values as input, directionalities of effects (protective vs. risk) are not considered in the current framework. However, it is important to consider the directionalities of effects to further elucidate biological mechanisms of phenotype-genotype association. Hence, extension of GGPA 2.0 by considering directionalities of effects will be an important and interesting future research direction. Third, in the current framework, functional annotations are considered at the SNP level. Using the gene- or pathway-level information will be an interesting direction and left as a future work. Fourth, GGPA 2.0 still relies on the assumption that SNPs are independent. While GWAS data preprocessing (e.g., SNP clumping) can help better satisfy this assumption, relaxation of this assumption will be an interesting work. Finally, as we discussed in Supplementary Section S3.2, other SNP processing approaches (e.g., SNP clumping) and potential impact and benefit of considering MAF of SNPs will be interesting and important issues to investigate.

With the aforementioned strengths and the planned improvement, we believe that GGPA 2.0 will be a powerful tool for the integrative analysis of GWAS and functional annotation data.

## Data Availability

Publicly available datasets were analyzed in this study. This data can be found here: The proposed statistical framework was implemented as an R package “GGPA2” and it is publicly available at https://dongjunchung.github.io/GGPA2/. GWAS summary statistics for ten diseases used in this paper are available from the GWAS Catalog (https://www.ebi.ac.uk/gwas/): systemic lupus erythematosus (SLE), rheumatoid arthritis (RA), ulcerative colitis (UC), Crohn’s disease (CD), type I diabetes (T1D), attention deficit-hyperactivity disorder (ADHD), autism spectrum disorder (ASD), bipolar disorder (BIP), schizophrenia (SCZ), and major depressive disorder (MDD). The two sets of functional annotations we used in this paper, including GenoSkyline and GenoSkyline-Plus, are available from http://zhaocenter.org/GenoSkyline.
