# Supplementary material for: graph-GPA 2.0: improving multi-disease genetic analysis with integration of functional annotation data
Source: Front Genet. 2023 Jul 12;14:1079198. doi: 10.3389/fgene.2023.1079198 (PMC10370274; doi:10.3389/fgene.2023.1079198)
Supplement: Supplementary file 1 [file DataSheet1.PDF]

Supplementary Material

1 MCMC SAMPLING

This section describes full details of Metropolis-within-Gibbs steps for the Bayesian inferences.

The joint posterior distribution of all parameters is written by

$$f(\{\boldsymbol{e}_t\},\{\mu_i\},\{\sigma_i^2\},\boldsymbol{\alpha},\boldsymbol{\gamma},\boldsymbol{\beta},\boldsymbol{G},\{E(i,j)\}|\{y_{it}\},\{\boldsymbol{a}_t\})$$
$$\propto \prod_{t=1}^{T}\prod_{i=1}^{n} f(y_{it}|e_{it},\mu_i,\sigma_i^2)\ \prod_{i=1}^{n} f(\mu_i,\sigma_i^2)\ \prod_{t=1}^{T} f(\boldsymbol{e}_t|\boldsymbol{\alpha},\boldsymbol{\gamma},\boldsymbol{\beta},\boldsymbol{G})$$
$$\prod_{i=1}^{n} f(\alpha_i)\ \prod_{i=1}^{n}\prod_{m=1}^{M} f(\gamma_{im}|u_{im})\ f(u_{im}|p_u)\ \prod_{i=1}^{n-1}\prod_{j>i} f(\beta_{ij})f(E(i,j))\ f(p_u)$$

S1. For each phenotype i and SNP t, update $e_{it} \sim \text{Bernoulli}(p_1^*)$ where

$$p_1^* = \left\{1 + \frac{\text{N}(y_{it};0,1)}{\exp\left(\alpha_i + \sum_{m=1}^{M}\gamma_{im}a_{mt} + \sum_{j\sim i}\beta_{ij}\ e_{jt}\right)\cdot \text{LN}(y_{it};\mu_i,\sigma_i^2)}\right\}^{-1}.$$

S2. For each i, update

$$\mu_i \sim N\left(\frac{\sigma_i^2\theta_\mu + \tau_\mu^2\sum_{\{t:e_{it}=1\}}\log y_{it}}{\sigma_i^2+\tau_\mu^2 n_i}, \frac{\sigma_i^2\tau_\mu^2}{\sigma_i^2+\tau_\mu^2 n_i}\right)$$

where $n_i = \sum_{t=1}^{T} e_{it}$.

S3. For each i, update

$$\sigma_i^2 \sim IG\left(a_\sigma + \frac{n_i}{2}, b_\sigma + \frac{\sum_{\{t:e_{it}=1\}}(\log y_{it}-\mu_i)^2}{2}\right)$$

where $n_i = \sum_{t=1}^{T} e_{it}$.

S4. For each i, update α_i with the Metropolis-Hastings step:

1. Draw α_i^q from $N(\alpha_i, s_\alpha^2)$. We set $s_\alpha = 0.1$.
2. Update $\alpha_i = \alpha_i^q$ with the acceptance probability

$$\min\left[1, \left\{\prod_{t=1}^{T}\frac{\text{C}(\boldsymbol{\alpha},\boldsymbol{\gamma},\boldsymbol{\beta},\boldsymbol{G},\boldsymbol{a}_t)}{\text{C}(\boldsymbol{\alpha}^q,\boldsymbol{\gamma},\boldsymbol{\beta},\boldsymbol{G},\boldsymbol{a}_t)}\frac{\exp(\alpha_i^q e_{it})}{\exp(\alpha_i e_{it})}\right\}\frac{N(\alpha_i^q;\theta_\alpha,\tau_\alpha^2)}{N(\alpha_i;\theta_\alpha,\tau_\alpha^2)}\right]$$

where $\boldsymbol{\alpha}^q = (\alpha_1,\ldots,\alpha_{i-1},\alpha_i^q,\alpha_{i+1},\ldots,\alpha_n)$.

S5a. For each (i,m) such that $u_{im} = 1$, update γ_{im} with the Metropolis-Hastings step:

1. Draw γ_{im}^q from $N^+(\gamma_{im}, s_\gamma^2)$. We set $s_\gamma = 0.05$.

2. Update $\gamma_{im} = \gamma_{im}^q$ with the acceptance probability

$$\min\left[1, \left\{\prod_{t=1}^{T} \frac{\mathrm{C}(\boldsymbol{\alpha}, \boldsymbol{\gamma}, \boldsymbol{\beta}, \boldsymbol{G}, \boldsymbol{a}_t)}{\mathrm{C}(\boldsymbol{\alpha}, \boldsymbol{\gamma}^q, \boldsymbol{\beta}, \boldsymbol{G}, \boldsymbol{a}_t)} \frac{\exp(\gamma_{im}^q a_{mt} e_{it})}{\exp(\gamma_{im} a_{mt} e_{it})}\right\} \frac{\Gamma(\gamma_{im}^q; a_\gamma, b_\gamma)}{\Gamma(\gamma_{im}; a_\gamma, b_\gamma)} \frac{N^+(\gamma_{im}; \gamma_{im}^q, s_\gamma^2)}{N^+(\gamma_{im}^q; \gamma_{im}, s_\gamma^2)}\right]$$

where $\boldsymbol{\gamma}^q = \{\gamma_{11}, \ldots, \gamma_{im}^q, \ldots, \gamma_{nM}\}$.

S5b. For each (i, m), update u_{im} with the reversible jump process:

1. If $u_{im} = 0$, let $u_{im}^q = 1$ and propose γ_{im}^q from $q(\gamma_{im}^q | u_{im}^q) = \Gamma(\gamma_{im}^q; a_\gamma, b_\gamma)$.
   If $u_{im} = 1$, let $u_{im}^q = 0$ and propose γ_{im}^q from $q(\gamma_{im}^q | u_{im}^q) = \delta_0(\gamma_{im}^q)$.
2. Update $(u_{im}, \gamma_{im}) = (u_{im}^q, \gamma_{im}^q)$ with the acceptance probability

$$\min\left[1, \left\{\prod_{t=1}^{T} \frac{\mathrm{C}(\boldsymbol{\alpha}, \boldsymbol{\gamma}, \boldsymbol{\beta}, \boldsymbol{G}, \boldsymbol{a}_t)}{\mathrm{C}(\boldsymbol{\alpha}, \boldsymbol{\gamma}^q, \boldsymbol{\beta}, \boldsymbol{G}, \boldsymbol{a}_t)} \frac{\exp(\gamma_{im}^q a_{mt} e_{it})}{\exp(\gamma_{im} a_{mt} e_{it})}\right\} \frac{f(\gamma_{im}^q | u_{im}^q) f(u_{im}^q | p_u)}{f(\gamma_{im} | u_{im}) f(u_{im} | p_u)} \frac{q(\gamma_{im} | u_{im}) q(u_{im} | u_{im}^q)}{q(\gamma_{im}^q | u_{im}^q) q(u_{im}^q | u_{im})}\right]$$

where $\boldsymbol{\gamma}^q = \{\gamma_{11}, \ldots, \gamma_{im}^q, \ldots, \gamma_{nM}\}$.
Note that several probabilities are cancelled:

- $q(u_{im}^q | u_{im}) = q(u_{im} | u_{im}^q) = 1$, because $\Pr(u_{im}^q = 1 - u_{im} | u_{im}^q) = 1$).
- $f(\gamma_{im}^q | u_{im}^q) = q(\gamma_{im}^q | u_{im}^q)$ and $f(\gamma_{im} | u_{im}) = q(\gamma_{im} | u_{im})$.

Then, the acc. prob. is shortened as

$$\min\left[1, \left\{\prod_{t=1}^{T} \frac{\mathrm{C}(\boldsymbol{\alpha}, \boldsymbol{\gamma}, \boldsymbol{\beta}, \boldsymbol{G}, \boldsymbol{a}_t)}{\mathrm{C}(\boldsymbol{\alpha}, \boldsymbol{\gamma}^q, \boldsymbol{\beta}, \boldsymbol{G}, \boldsymbol{a}_t)} \frac{\exp(\gamma_{im}^q a_{mt} e_{it})}{\exp(\gamma_{im} a_{mt} e_{it})}\right\} \frac{f(u_{im}^q | p_u)}{f(u_{im} | p_u)}\right].$$

S5c. Update p_u from Beta $\left(1 + \sum_{i=1}^{n} \sum_{m=1}^{M} u_{im}, 1 + \sum_{i=1}^{n} \sum_{m=1}^{M} (1 - u_{im})\right)$.

S6. For each (i, j) such that $E(i, j) = 1$, update β_{ij} with the Metropolis-Hastings:

1. Draw β_{ij}^q from $N_+(\beta_{ij}, s_\beta^2)$ where N_+ denotes the truncated normal distribution bounded above zero. We set $s_\beta = 0.1$.
2. Update $\beta_{ij} = \beta_{ij}^q$ with the acceptance probability

$$\min\left[1, \left\{\prod_{t=1}^{T} \frac{\mathrm{C}(\boldsymbol{\alpha}, \boldsymbol{\gamma}, \boldsymbol{\beta}, \boldsymbol{G}, \boldsymbol{a}_t)}{\mathrm{C}(\boldsymbol{\alpha}, \boldsymbol{\gamma}, \boldsymbol{\beta}^q, \boldsymbol{G}, \boldsymbol{a}_t)} \frac{\exp(\beta_{ij}^q e_{it} e_{jt})}{\exp(\beta_{ij} e_{it} e_{jt})}\right\} \frac{\Gamma(\beta_{ij}^q; a_\beta, b_\beta)}{\Gamma(\beta_{ij}; a_\beta, b_\beta)} \frac{N_+(\beta_{ij}; \beta_{ij}^q, s_\beta^2)}{N_+(\beta_{ij}^q; \beta_{ij}, s_\beta^2)}\right]$$

where $\boldsymbol{\beta}^q = (\beta_{12}, \beta_{13}, \ldots, \beta_{i,j-1}, \beta_{ij}^q, \beta_{i,j+1}, \ldots, \beta_{n-1,n-2}, \beta_{n-1,n})$.

S7. For a randomly chosen (i, j) among non-forced-in edges, update $(\beta_{ij}, \boldsymbol{G})$ by the reversible jump process (Note that we do not update the forced-in edges, i.e., we fix $E(i, j) = 1$ for the forced-in edges over the MCMC iterations):

1. Let z denote the number of edges in the current graph $\boldsymbol{G}$, i.e., $z = \sum_{\{(i,j):i \neq j\}} E(i, j)$ and z_{force} denote the number of forced-in edges. Propose the number of edges E^q from the proposal distribution,

$$q(z^q \mid z) = 0.5\, I\left[z^q = z - 1\right] + 0.5\, I\left[z^q = z + 1\right].$$

If $z = z_{\text{force}}$, set $z^q = z + 1$ with probability 1. If $z = z_{\max}$, set $z^q = z_{\max} - 1$ with probability 1 where $z_{\max}$ denotes the maximum number of possible edges, i.e., $z_{\max} = \binom{n}{2}$.

2. Propose $\boldsymbol{G}^q$ from the proposal distribution $q(\boldsymbol{G}^q|\boldsymbol{G}, z^q)$ and then β_{ij}^q from the proposal distribution $q(\beta_{ij}^q|\boldsymbol{G}^q, z^q)$.
   a. For the case where $z^q > z$, randomly select a pair of (i,j) such that $E(i,j) = 0$ and let $E(i,j)^q = 1$ with the proposal distribution
   $$q(\boldsymbol{G}^q|\boldsymbol{G}, z^q) = \frac{1}{\#\{(i^*,j^*) : G_{i^*j^*} = 0\}} = \frac{1}{z_{\text{max}} - z}$$
   while $G_{i^*j^*}^q = G_{i^*j^*}$ for all other (i^*,j^*). Propose β_{ij}^q from $q(\beta_{ij}^q|E(i,j)^q, z^q) = \Gamma(\beta_{ij}^q; a_{\beta_G}, b_{\beta_G})$. We set $a_{\beta_G} = b_{\beta_G} = 1$.
   b. For the case where $z^q < z$, randomly select a non-forced-in edge (i,j) such that $E(i,j) = 1$, and let $E(i,j)^q = 0$ with the proposal distribution
   $$q(\boldsymbol{G}^q|\boldsymbol{G}, z^q) = \frac{1}{\#\{(i^*,j^*) : G_{i^*j^*} = 1\}} = \frac{1}{z - z_{\text{force}}}$$
   while $G_{i^*j^*}^q = G_{i^*j^*}$ for all other (i^*,j^*). Propose β_{ij}^q from $q(\beta_{ij}^q|E(i,j)^q, z^q) = \delta_0(\beta_{ij}^q)$.
3. Update $(\beta_{ij}, \boldsymbol{G}) = (\beta_{ij}^q, \boldsymbol{G}^q)$ with the acceptance probability
$$\min\left[1, \left\{\prod_{t=1}^{T} \frac{\text{C}(\boldsymbol{\alpha},\boldsymbol{\gamma},\boldsymbol{\beta},\boldsymbol{G},\boldsymbol{a}_t)}{\text{C}(\boldsymbol{\alpha},\boldsymbol{\gamma},\boldsymbol{\beta}^q,\boldsymbol{G}^q,\boldsymbol{a}_t)} \frac{\exp(\beta_{ij}^q e_{it} e_{jt})}{\exp(\beta_{ij} e_{it} e_{jt})}\right\} \frac{f(\beta_{ij}^q|E(i,j)^q)}{f(\beta_{ij}|E(i,j))} \frac{q(\beta_{ij}|\boldsymbol{G}, z)q(\boldsymbol{G}|\boldsymbol{G}^q, z)q(z|z^q)}{q(\beta_{ij}^q|\boldsymbol{G}^q, z^q)q(\boldsymbol{G}^q|\boldsymbol{G}, z^q)q(z^q|z)}\right]$$
   where $\boldsymbol{b}eta^q = (\beta_{12}, \beta_{13}, \ldots, \beta_{i,j-1}, \beta_{ij}^q, \beta_{i,j+1}, \ldots, \beta_{n-1,n-2}, \beta_{n-1,n})$ and $\boldsymbol{G}^q = (G_{12}, G_{13}, \ldots, G_{i,j-1}, E(i,j)^q, G_{i,j+1}, \ldots, G_{n-1,n-2}, G_{n-1,n})$.

Note that $f(\beta_{ij}|E(i,j)) = q(\beta_{ij}|\boldsymbol{G}, z)$ when $z^q > z$ and $f(\beta_{ij}^q|E(i,j)^q) = q(\beta_{ij}^q|\boldsymbol{G}^q, z^q)$ when $z^q < z$ and, so they are canceled out from the acceptance probability.

2 SIMULATIONS RESULTS

2.1 Simulation Setting #1

$$\text{The simulation coefficient } \Gamma_1 = \begin{bmatrix} 2 & 0 & 0 & 0 & 0 \\ 2 & 0 & 0 & 0 & 0 \\ 2 & 0 & 0 & 0 & 0 \\ 0 & 2 & 0 & 0 & 0 \\ 0 & 0 & 2 & 0 & 0 \\ 0 & 0 & 0 & 2 & 0 \end{bmatrix}$$

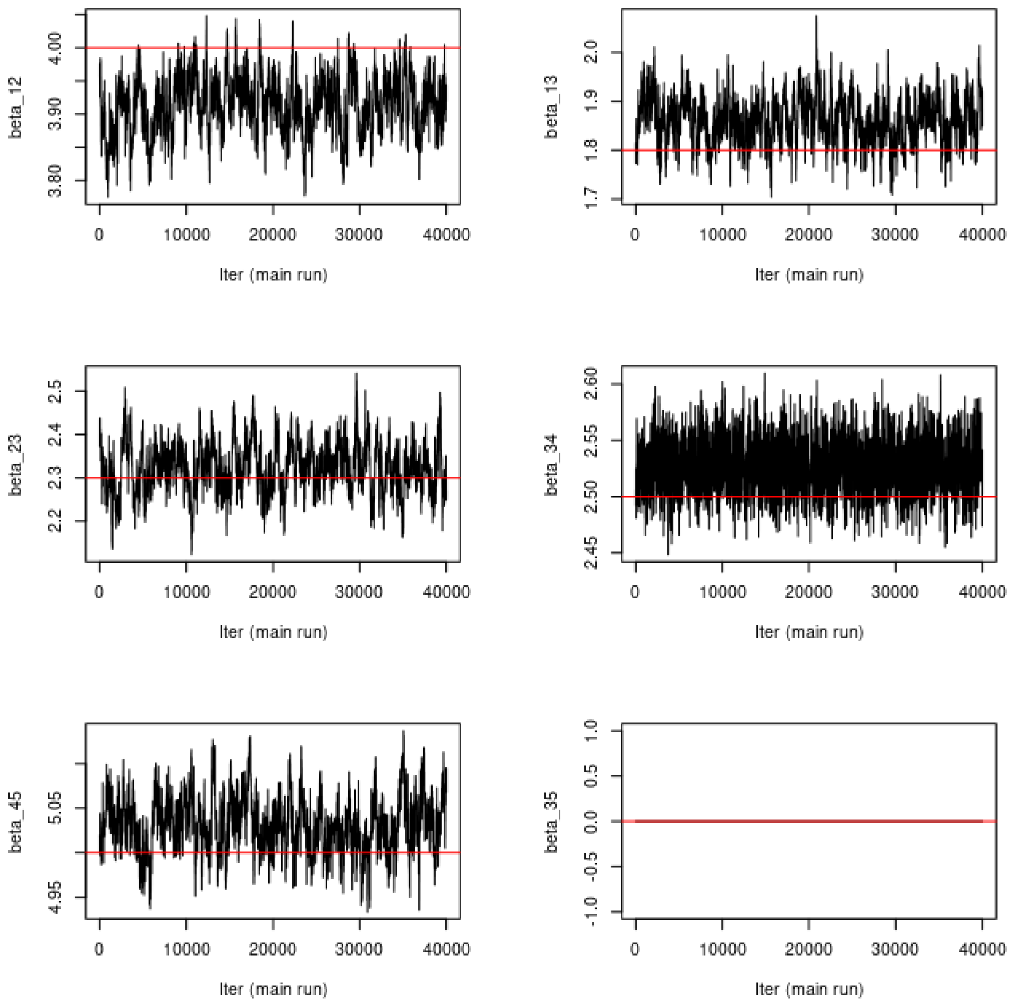

Figure S1: Simulation study with Γ_1 using annotation data: Trace plot of β. Red lines are true values.

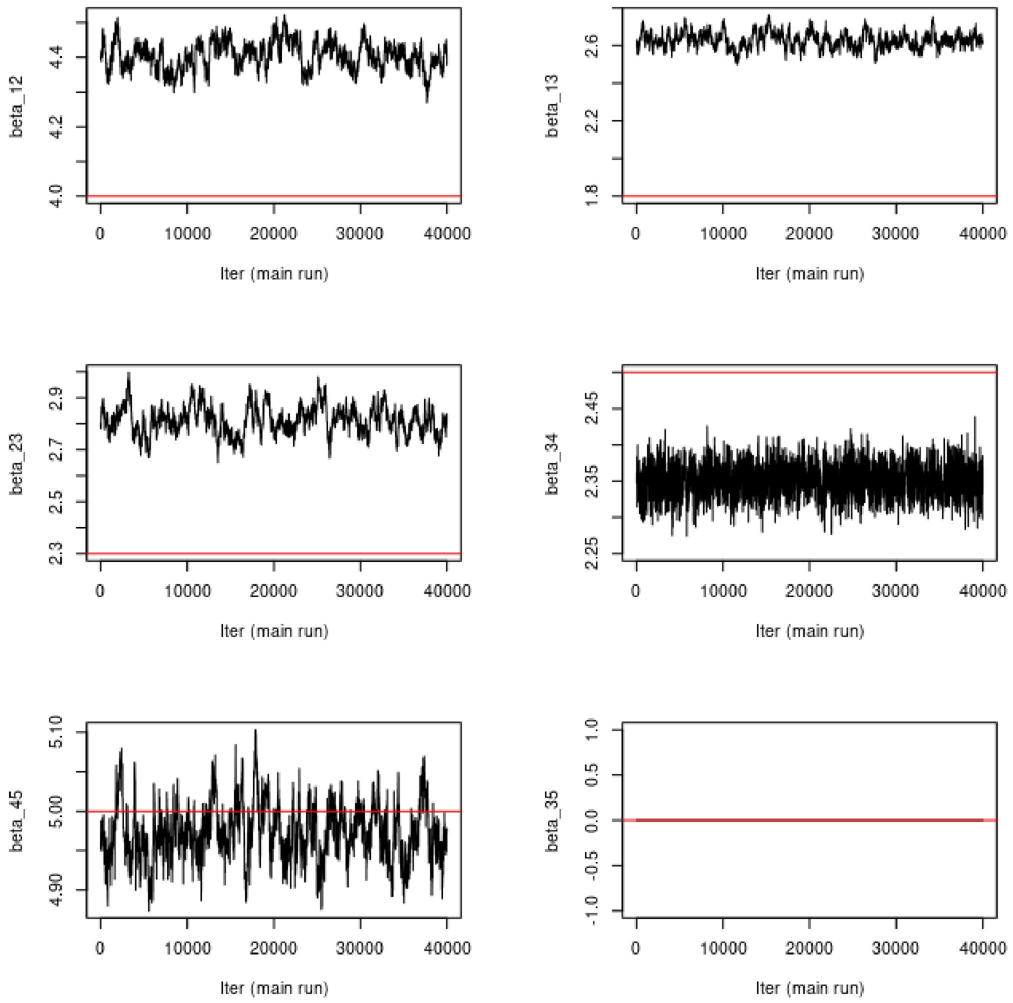

Figure S2: Simulation study with Γ_1 without using annotation data: Trace plot of β. Red lines are true values.

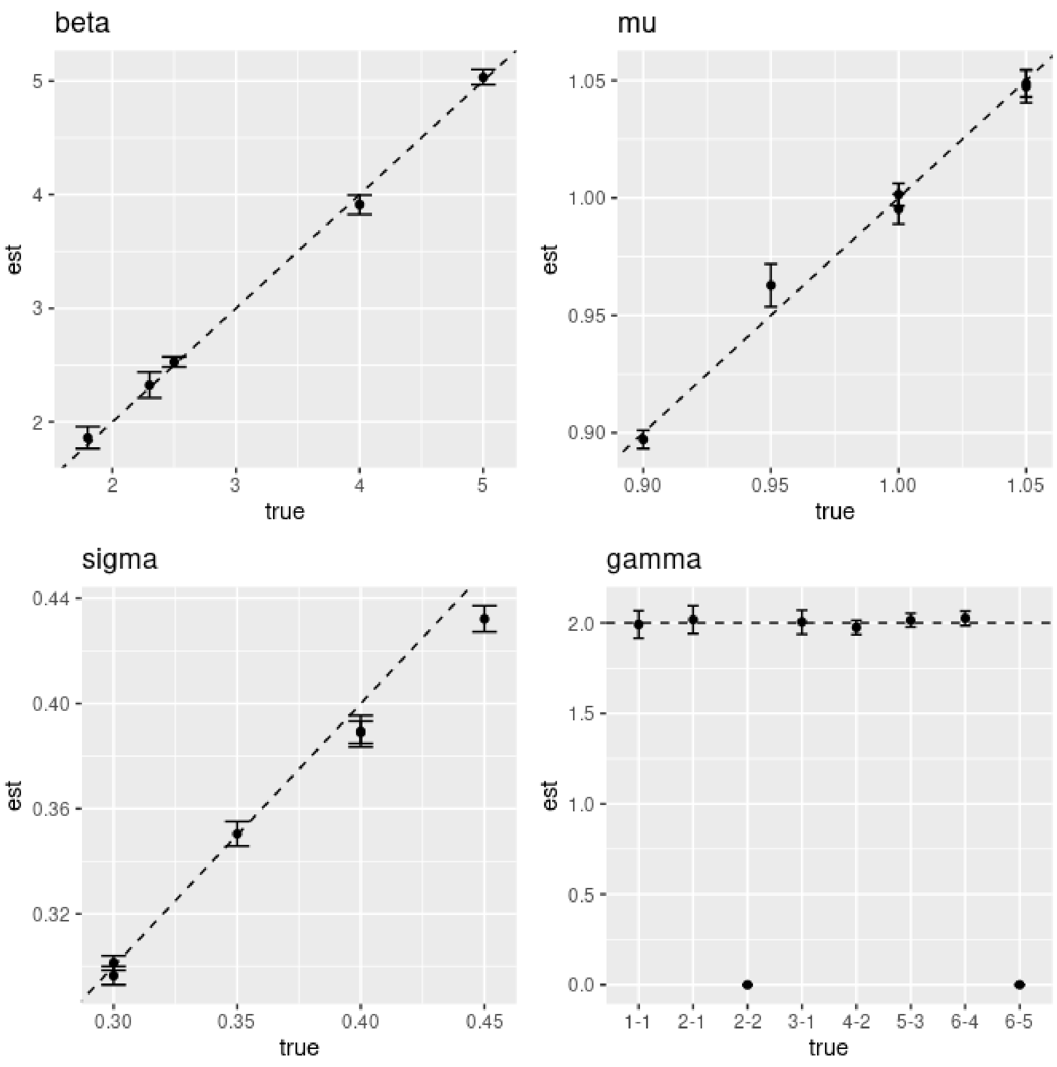

Figure S3: Simulation study with Γ_1 using annotation data: Parameter estimation.

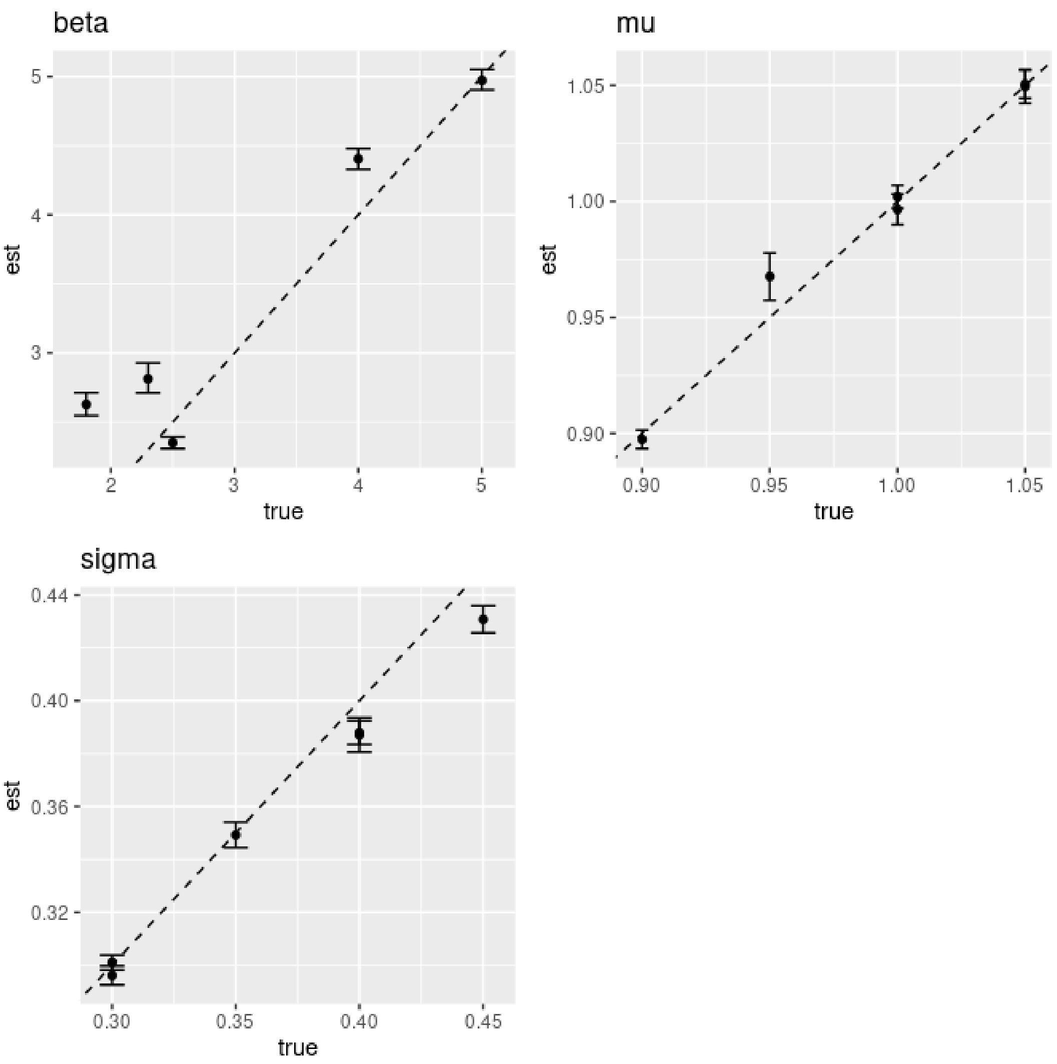

Figure S4: Simulation study with Γ_1 without using annotation data: Parameter estimation.

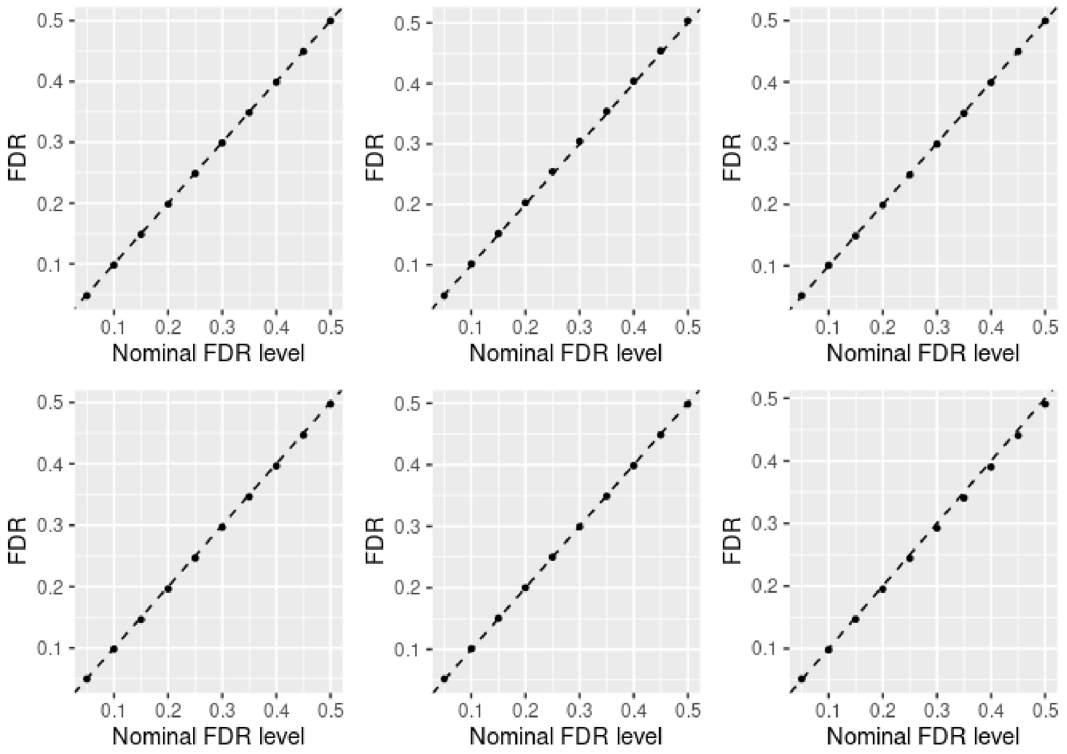

Figure S5: Simulation study with Γ_1 using annotation data: False discovery rate control.

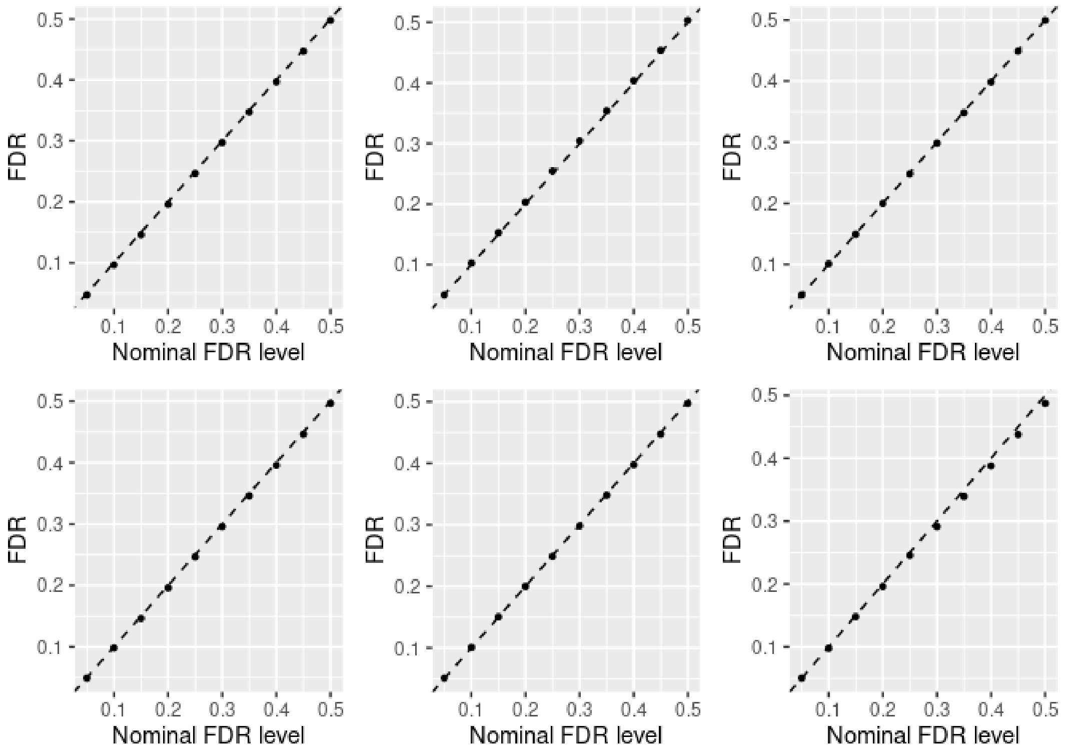

Figure S6: Simulation study with Γ_1 without using annotation data: False discovery rate control.

	P1	P2	P3	P4	P5	P6
P1	22216	21202	13581	6388	5722	1005
P2	21202	27667	14522	7139	6442	1173
P3	13581	14522	15186	6767	5862	632
P4	6388	7139	6767	18960	16600	782
P5	5722	6442	5862	16600	21278	857
P6	1005	1173	632	782	857	11270

Table S1. Simulation study with Γ_1 using annotation data: Numbers of SNPs identified to be associated with each pair of phenotypes with the global FDR at nominal level of 5%. Diagonal elements show the number of SNPs inferred to be associated with each phenotype when the global FDR is controlled at the same level.

	P1	P2	P3	P4	P5	P6
P1	21569	20561	13017	6246	5594	780
P2	20561	26709	13973	6989	6314	918
P3	13017	13973	14634	6577	5715	494
P4	6246	6989	6577	18432	16172	628
P5	5594	6314	5715	16172	20601	683
P6	780	918	494	628	683	9512

Table S2. Simulation study with Γ_1 without using annotation data: Numbers of SNPs identified to be associated with each pair of phenotypes with the global FDR at nominal level of 5%. Diagonal elements show the number of SNPs inferred to be associated with each phenotype when the global FDR is controlled at the same level.

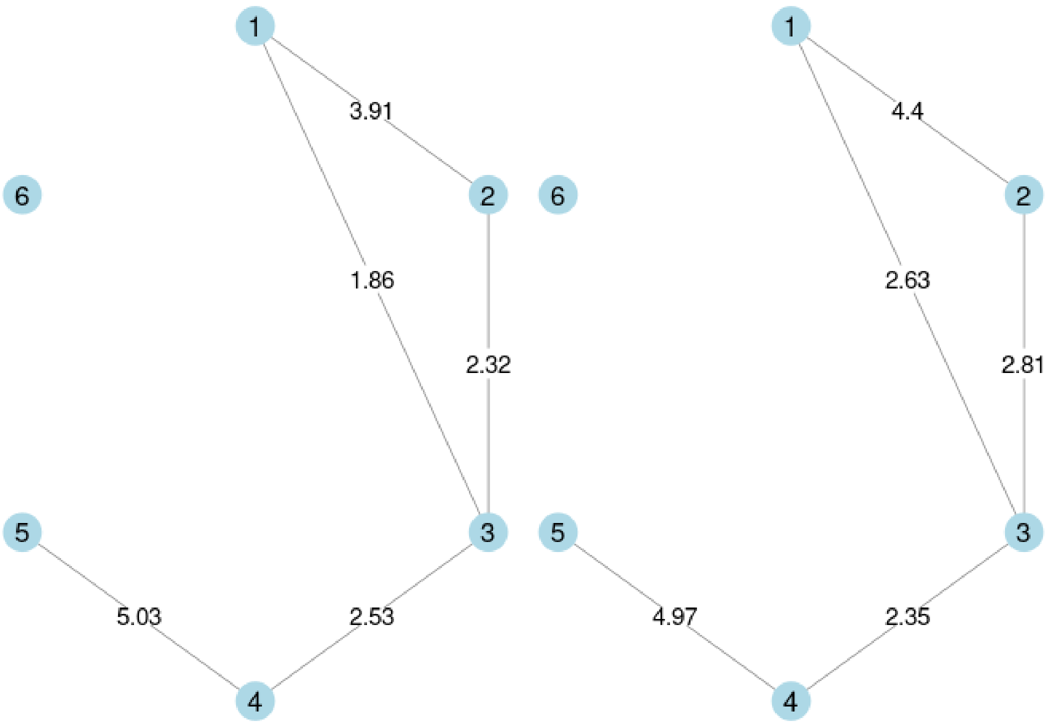

Figure S7: Simulation study with Γ_1: Phenotype graphs estimated using annotation data (left) and without using annotation data (right).

2.2 Simulation Setting #2

$$\text{The simulation coefficient } \Gamma_2 = \begin{bmatrix} 1 & 0 & 0 & 0 & 0 \\ 1 & 0 & 0 & 0 & 0 \\ 1 & 0 & 0 & 0 & 0 \\ 0 & 2 & 0 & 0 & 0 \\ 0 & 2 & 0 & 0 & 0 \\ 0 & 2 & 0 & 0 & 0 \end{bmatrix}$$

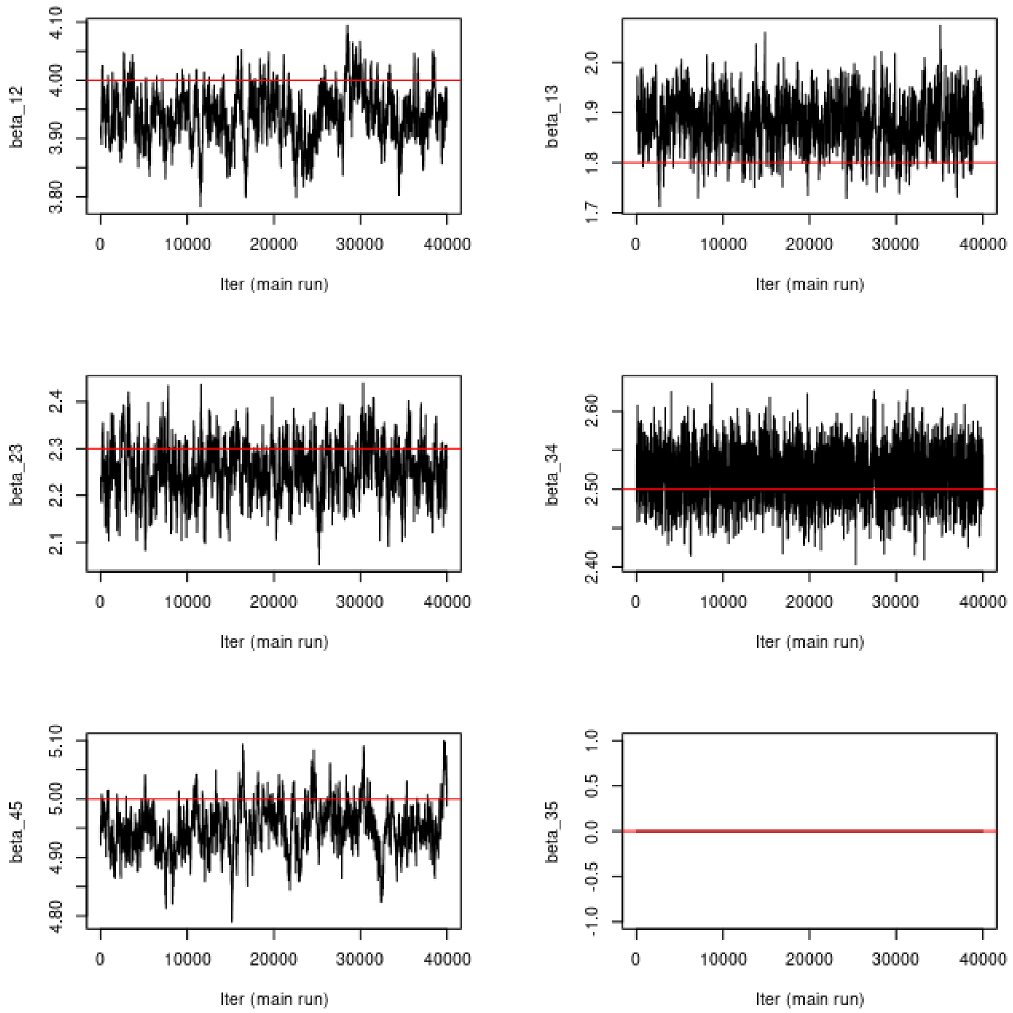

Figure S8: Simulation study with Γ_2 using annotation data: Trace plot of β. Red lines are true values.

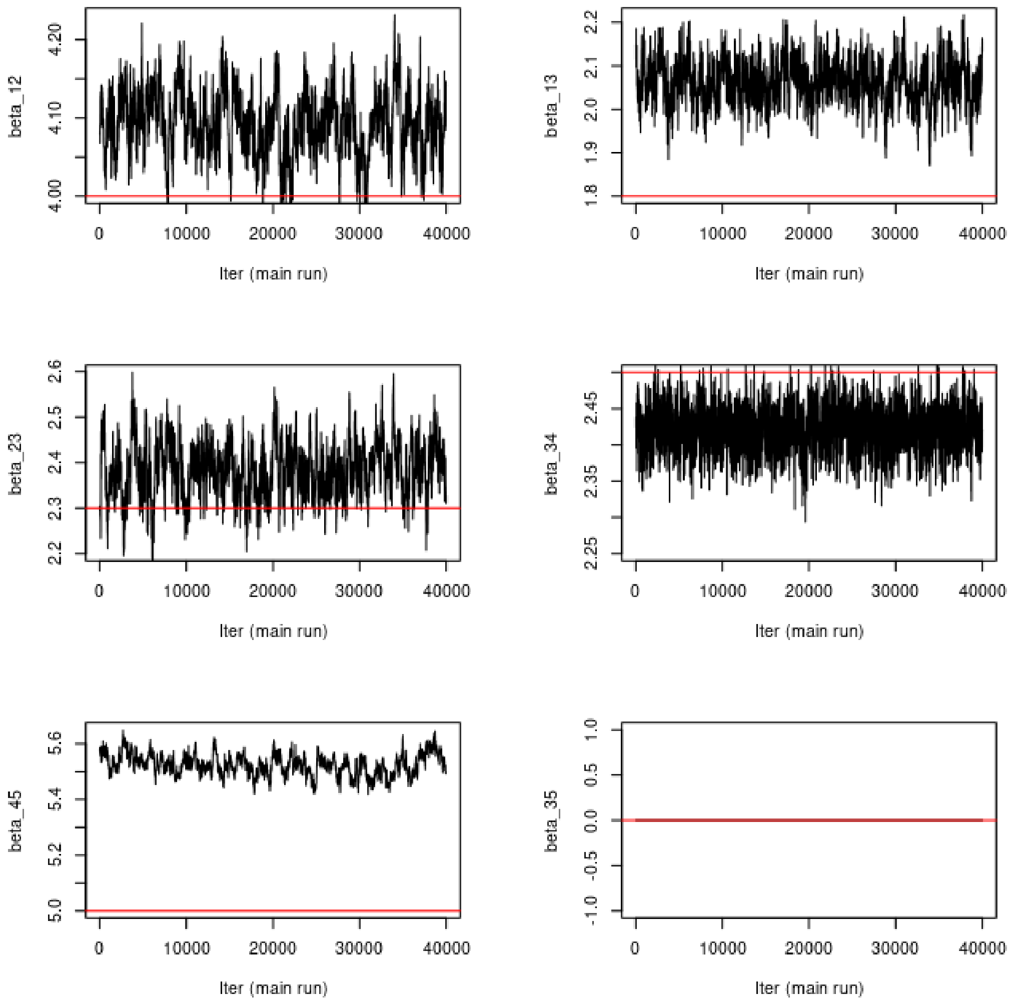

Figure S9: Simulation study with Γ_2 without using annotation data: Trace plot of β. Red lines are true values.

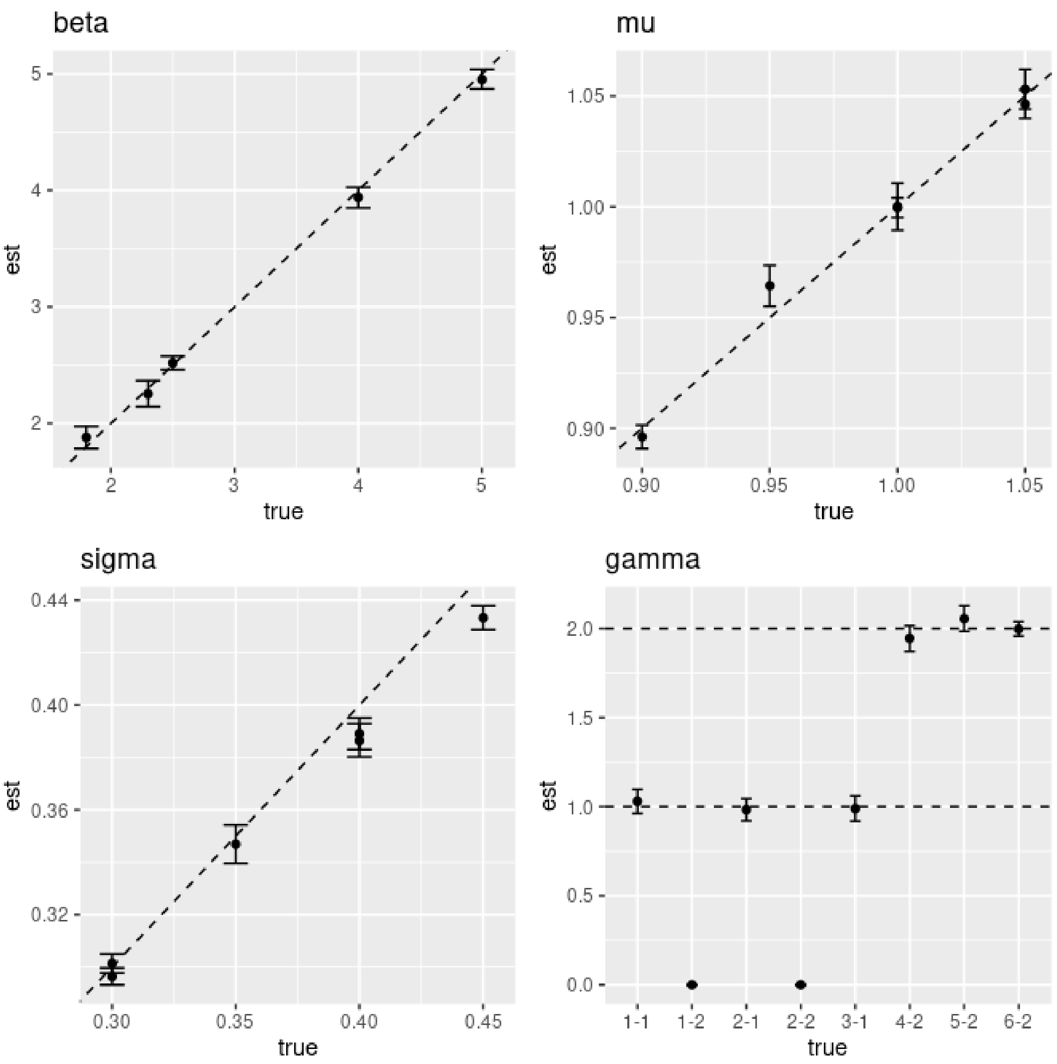

Figure S10: Simulation study with Γ_2 using annotation data: Parameter estimation.

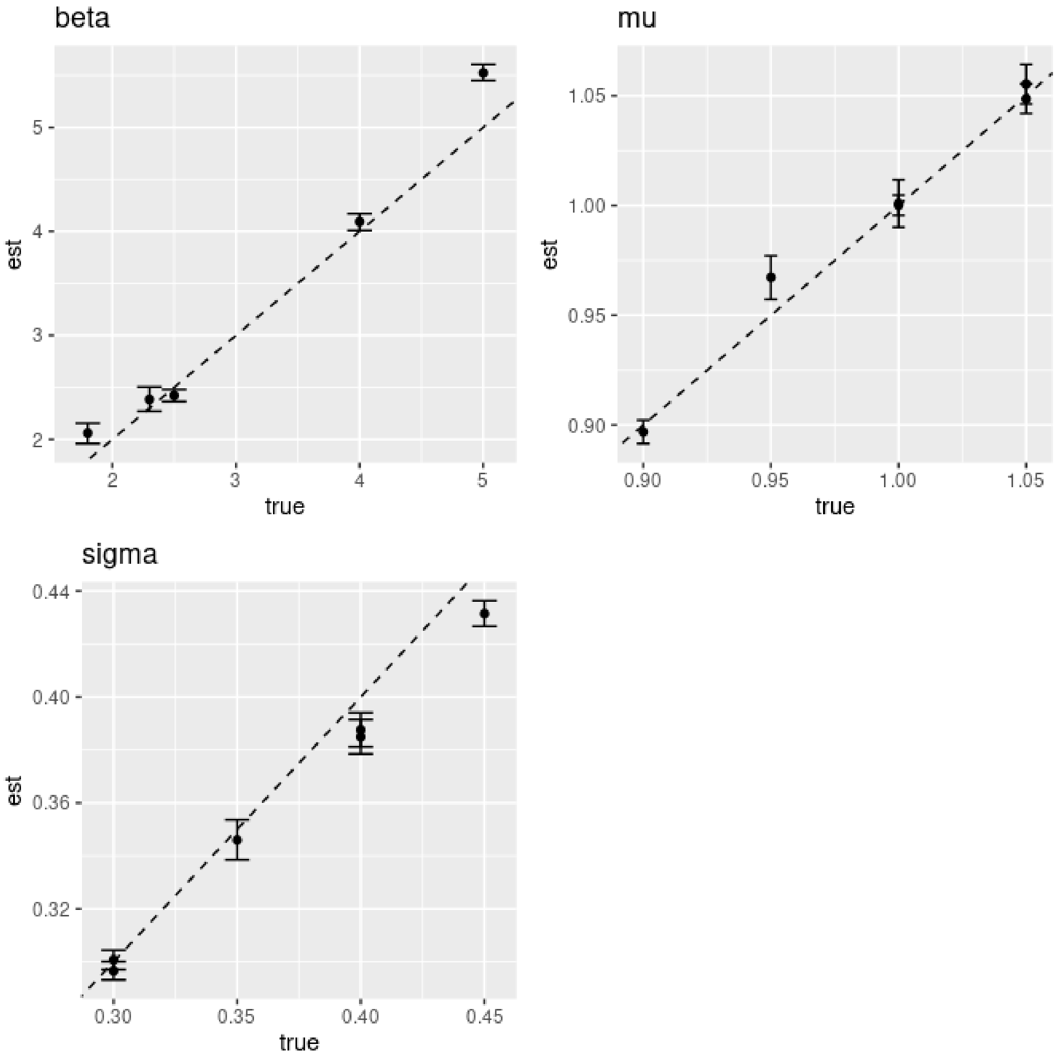

Figure S11: Simulation study with Γ_2 without using annotation data: Parameter estimation.

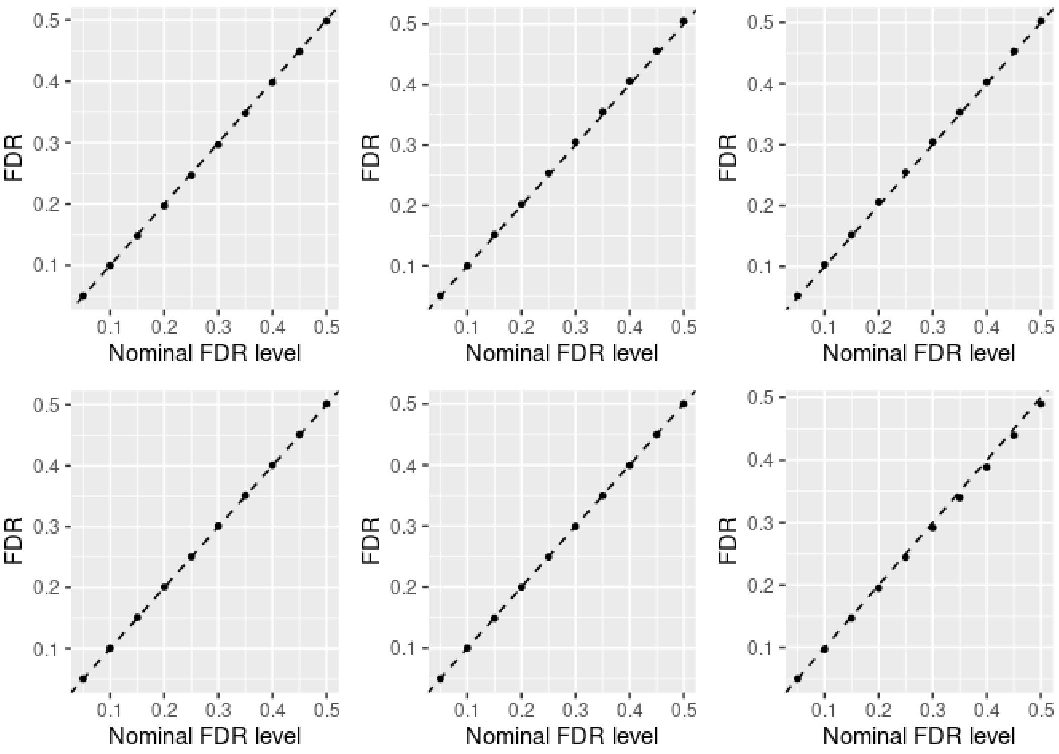

Figure S12: Simulation study with Γ_2 using annotation data: False discovery rate control.

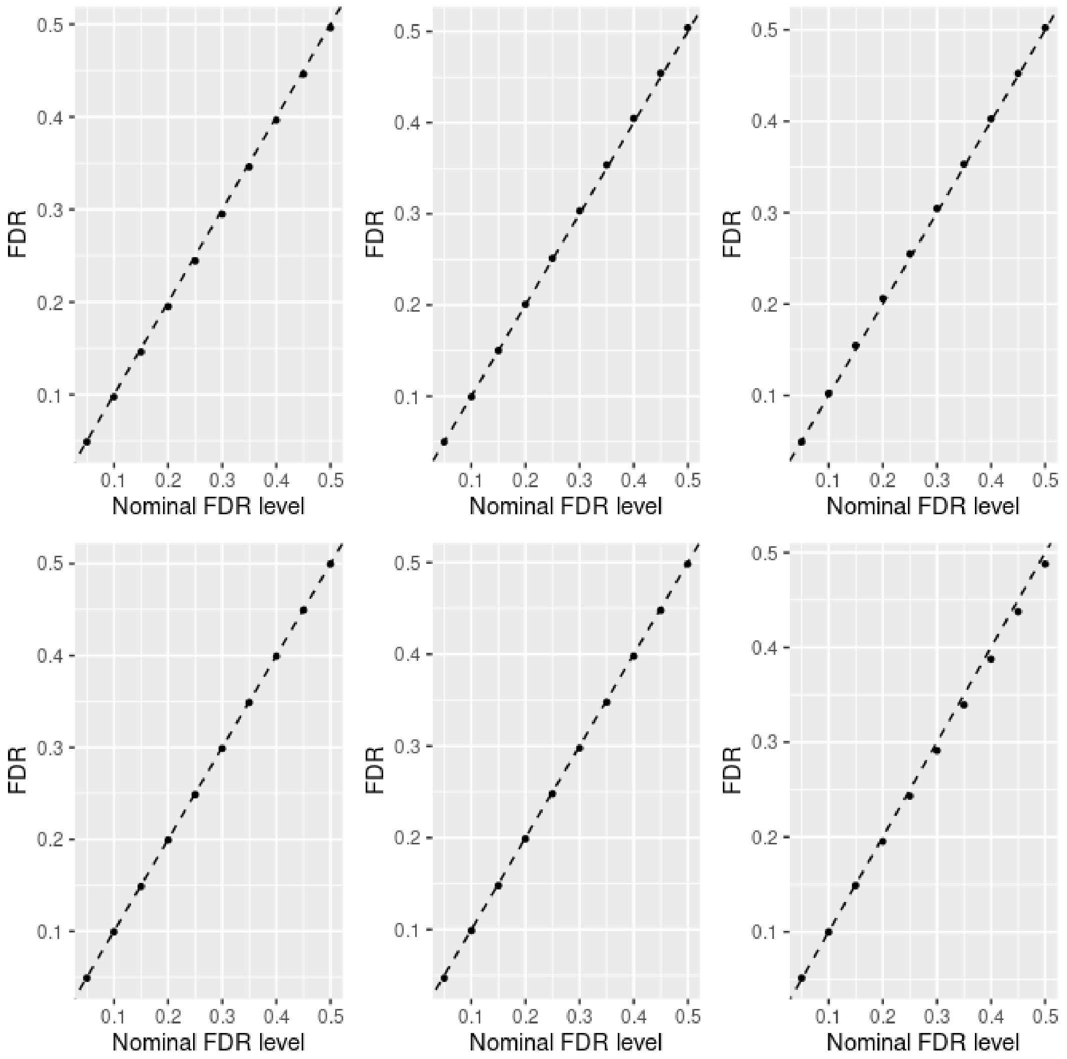

Figure S13: Simulation study with Γ_2 without using annotation data: False discovery rate control.

	P1	P2	P3	P4	P5	P6
P1	10758	9638	4235	2915	2768	631
P2	9638	14995	4934	3683	3529	793
P3	4235	4934	5731	3360	3069	494
P4	2915	3683	3360	20956	19560	3535
P5	2768	3529	3069	19560	23337	3757
P6	631	793	494	3535	3757	11147

Table S3. Simulation study with Γ_2 using annotation data: Numbers of SNPs identified to be associated with each pair of phenotypes with the global FDR at nominal level of 5%. Diagonal elements show the number of SNPs inferred to be associated with each phenotype when the global FDR is controlled at the same level.

	P1	P2	P3	P4	P5	P6
P1	10470	9335	4068	2833	2689	559
P2	9335	14486	4748	3558	3404	694
P3	4068	4748	5545	3248	2959	425
P4	2833	3558	3248	20208	18769	2879
P5	2689	3404	2959	18769	22476	3046
P6	559	694	425	2879	3046	10078

Table S4. Simulation study with Γ_2 without using annotation data: Numbers of SNPs identified to be associated with each pair of phenotypes with the global FDR at nominal level of 5%. Diagonal elements show the number of SNPs inferred to be associated with each phenotype when the global FDR is controlled at the same level.

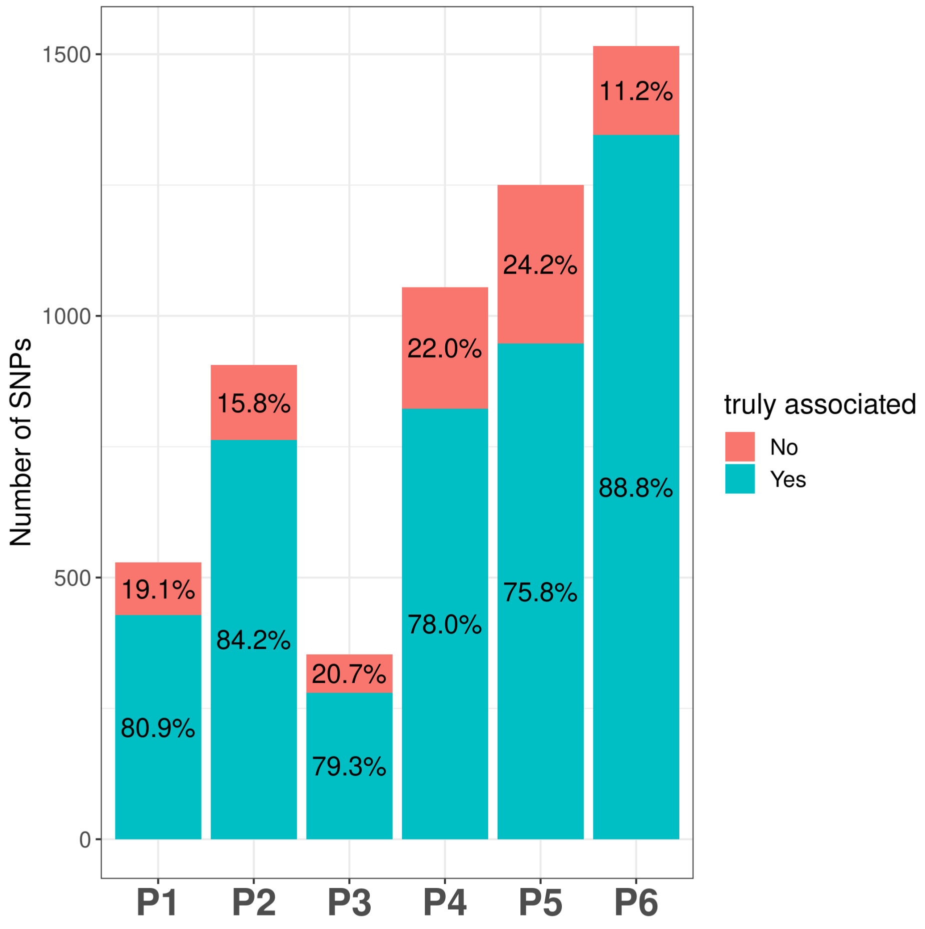

Figure S14: Simulation study with Γ_2: Extra SNPs identified by using annotation data. Most of the extra SNPs are truly associated with phenotypes.

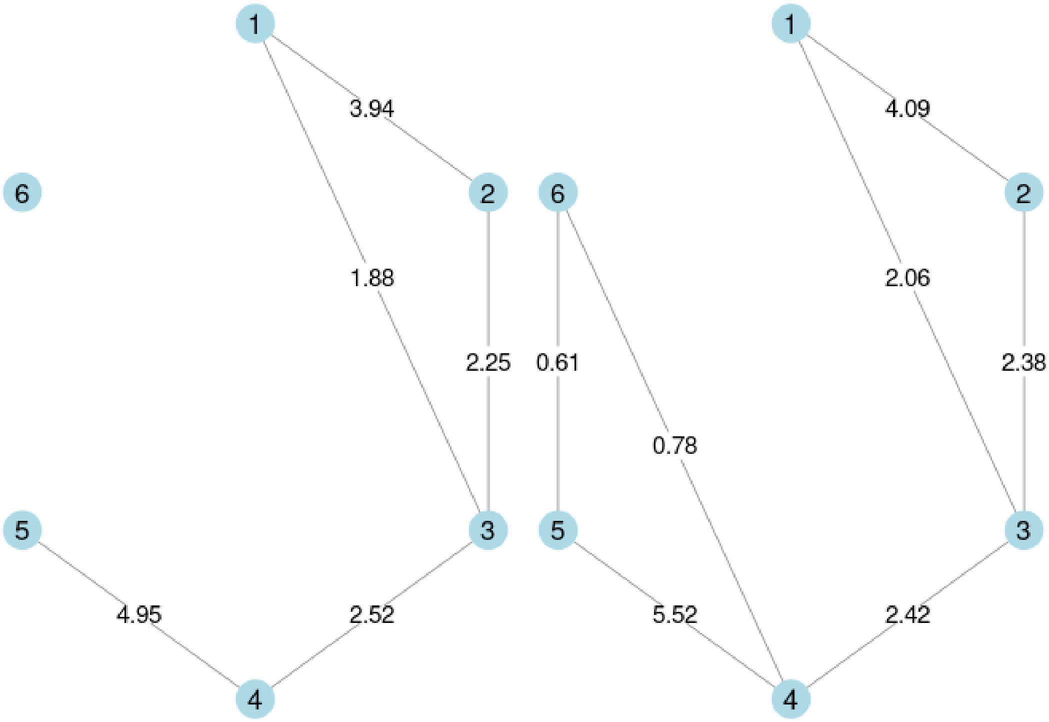

Figure S15: Simulation study with Γ_2: Phenotype graphs estimated using annotation data (left) and without using annotation data (right).

2.3 Simulation Setting #3

The simulation coefficient $\Gamma_3 = \begin{bmatrix} 0 & 0 & 0 & 0 & 0 \\ 0 & 0 & 0 & 0 & 0 \\ 0 & 0 & 0 & 0 & 0 \\ 0 & 2 & 2 & 0 & 0 \\ 2 & 2 & 0 & 2 & 0 \\ 2 & 0 & 2 & 2 & 2 \end{bmatrix}$

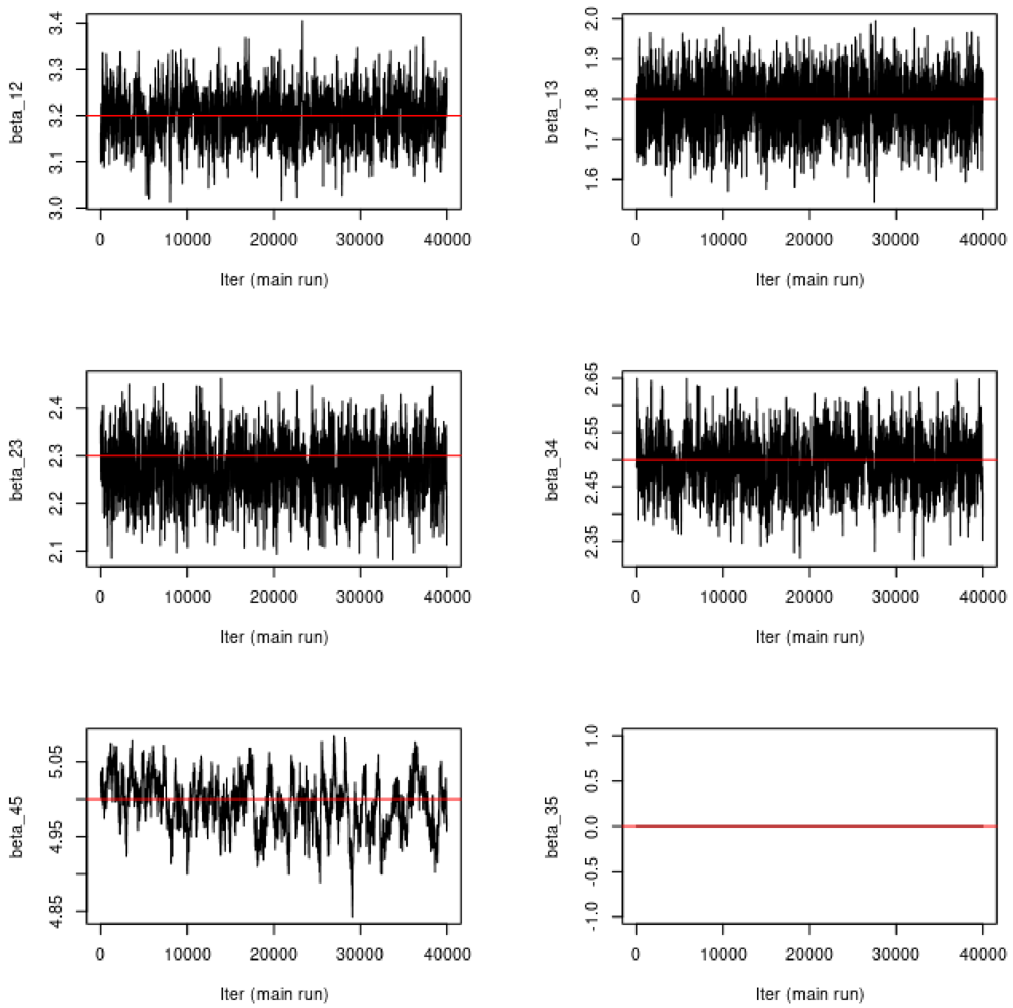

Figure S16: Simulation study with Γ_3 using annotation data: Trace plot of β. Red lines are true values.

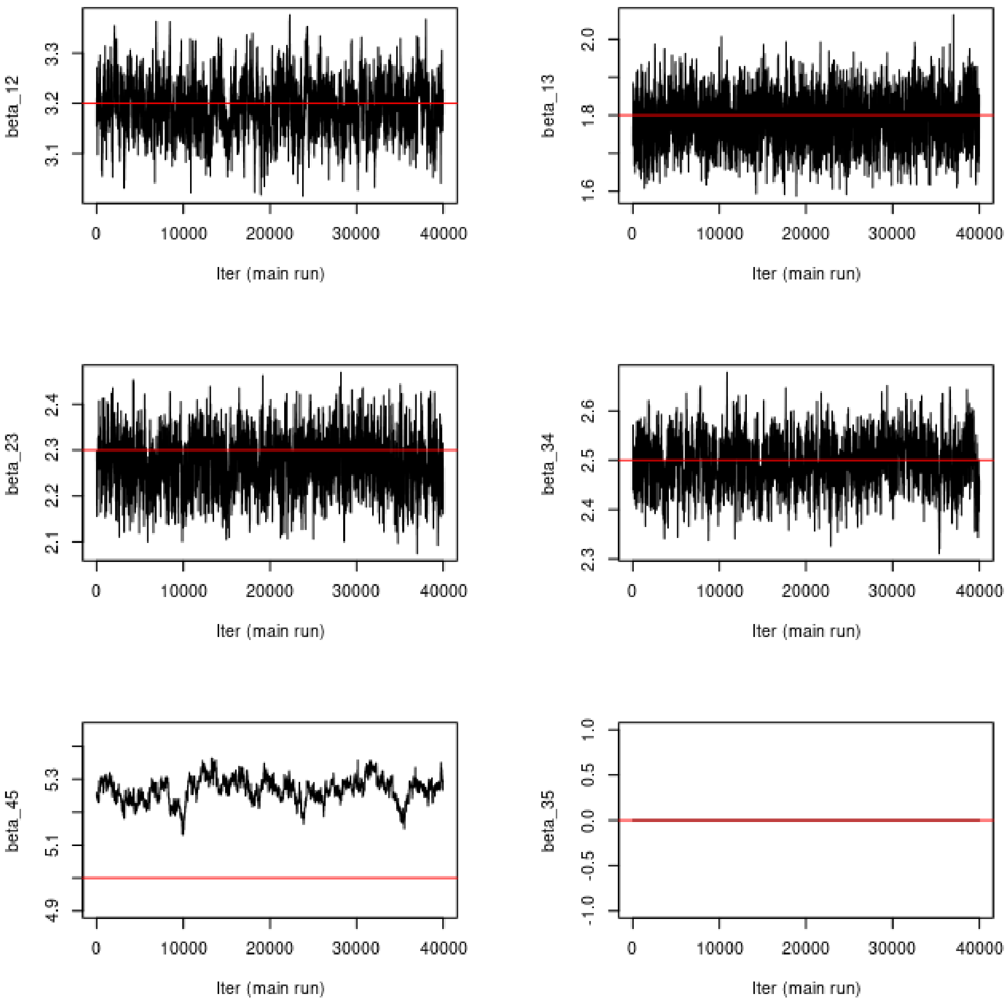

Figure S17: Simulation study with Γ_3 without using annotation data: Trace plot of β. Red lines are true values.

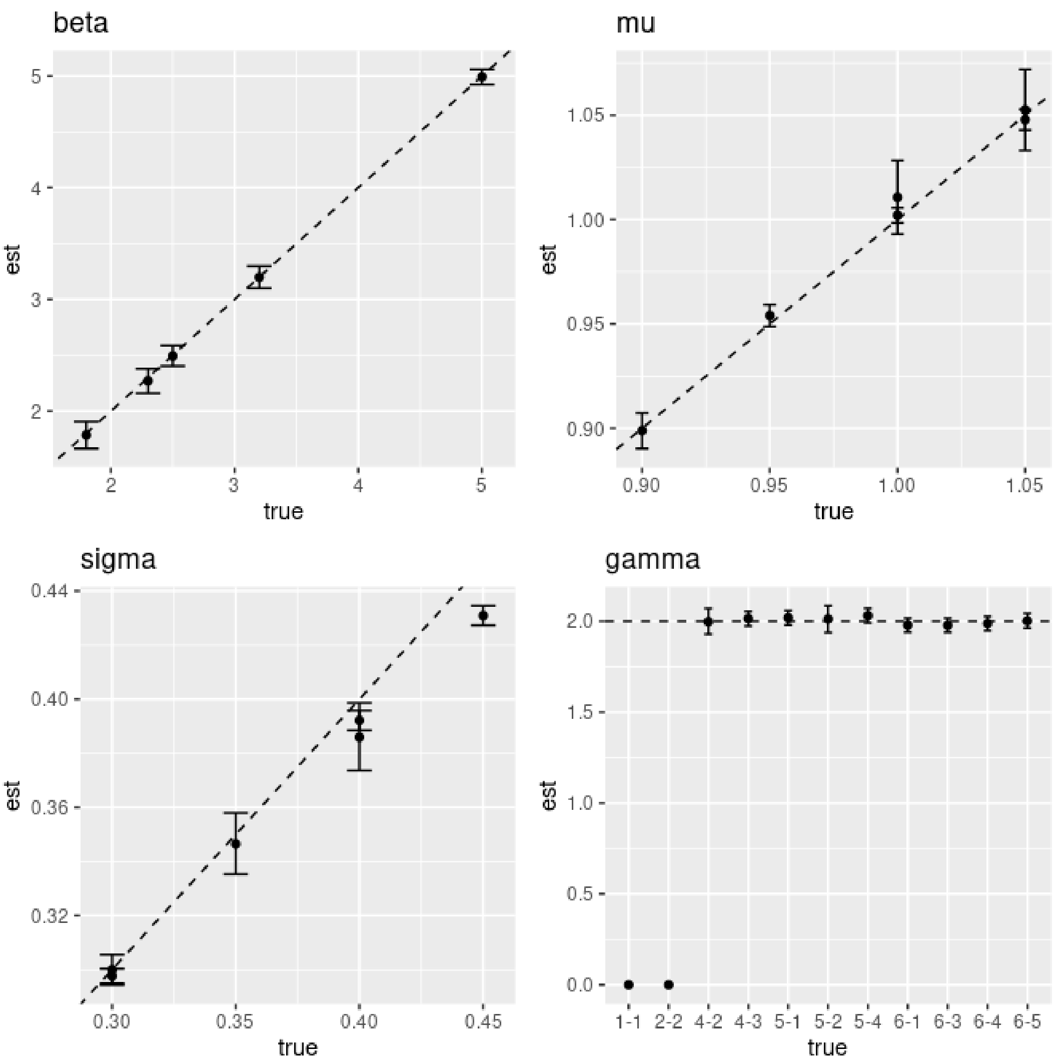

Figure S18: Simulation study with Γ_3 using annotation data: Parameter estimation.

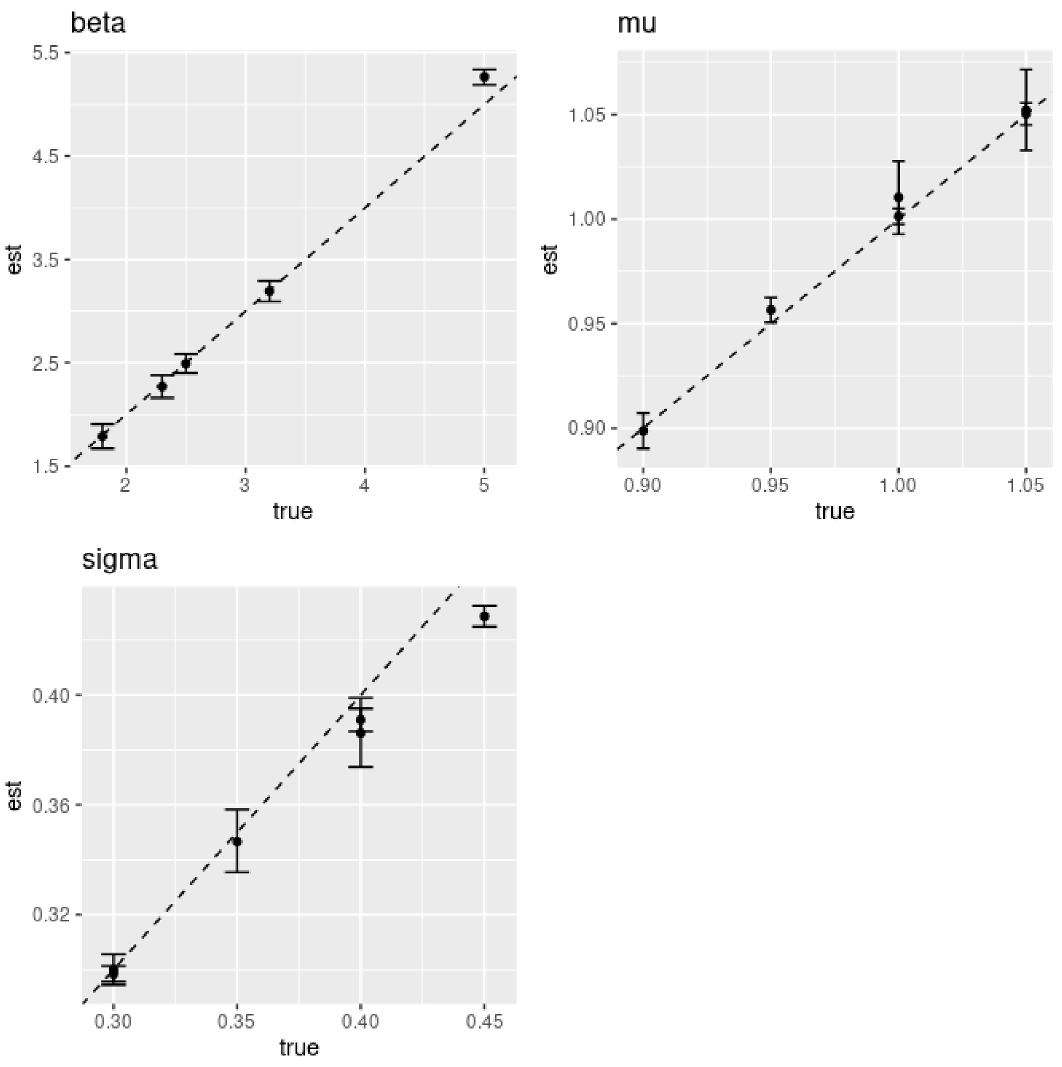

Figure S19: Simulation study with Γ_3 without using annotation data: Parameter estimation.

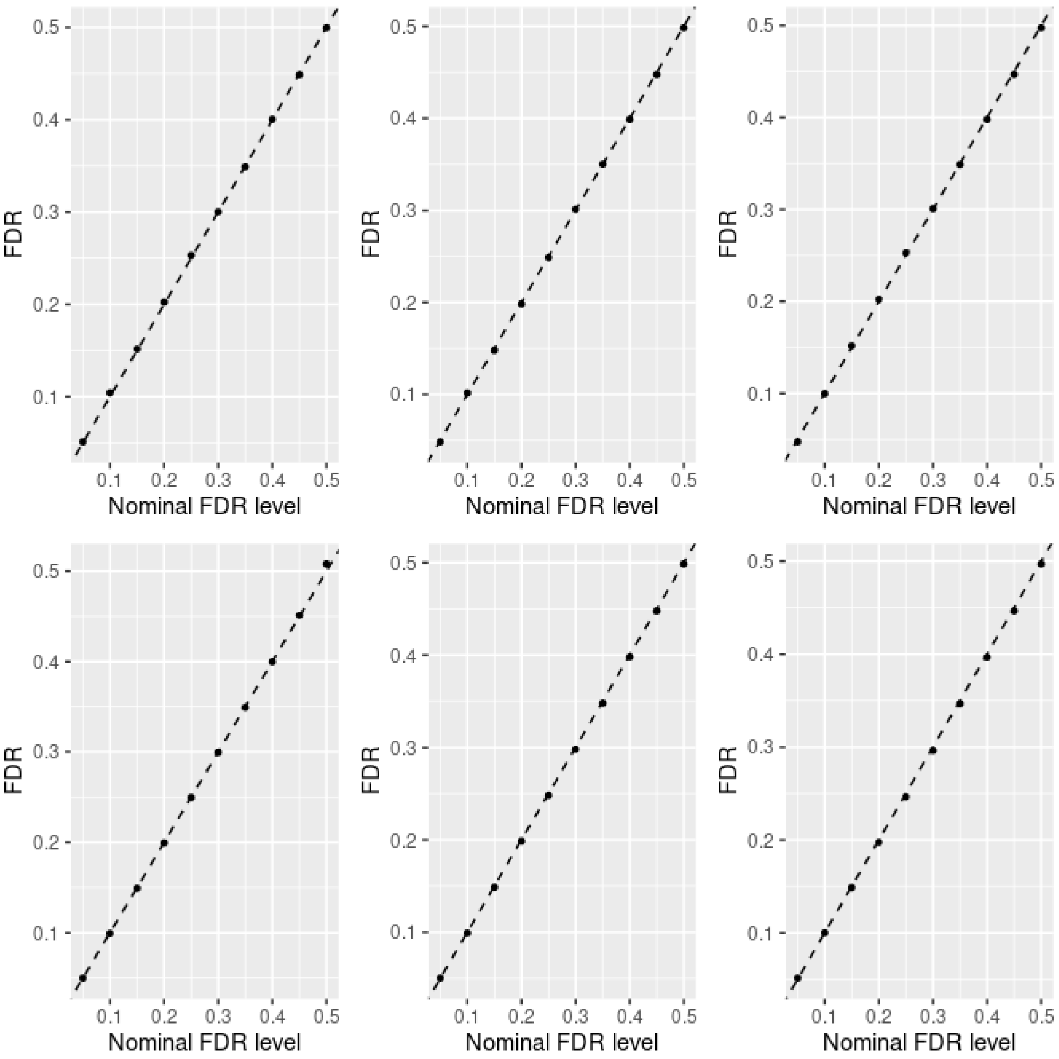

Figure S20: Simulation study with Γ_3 using annotation data: False discovery rate control.

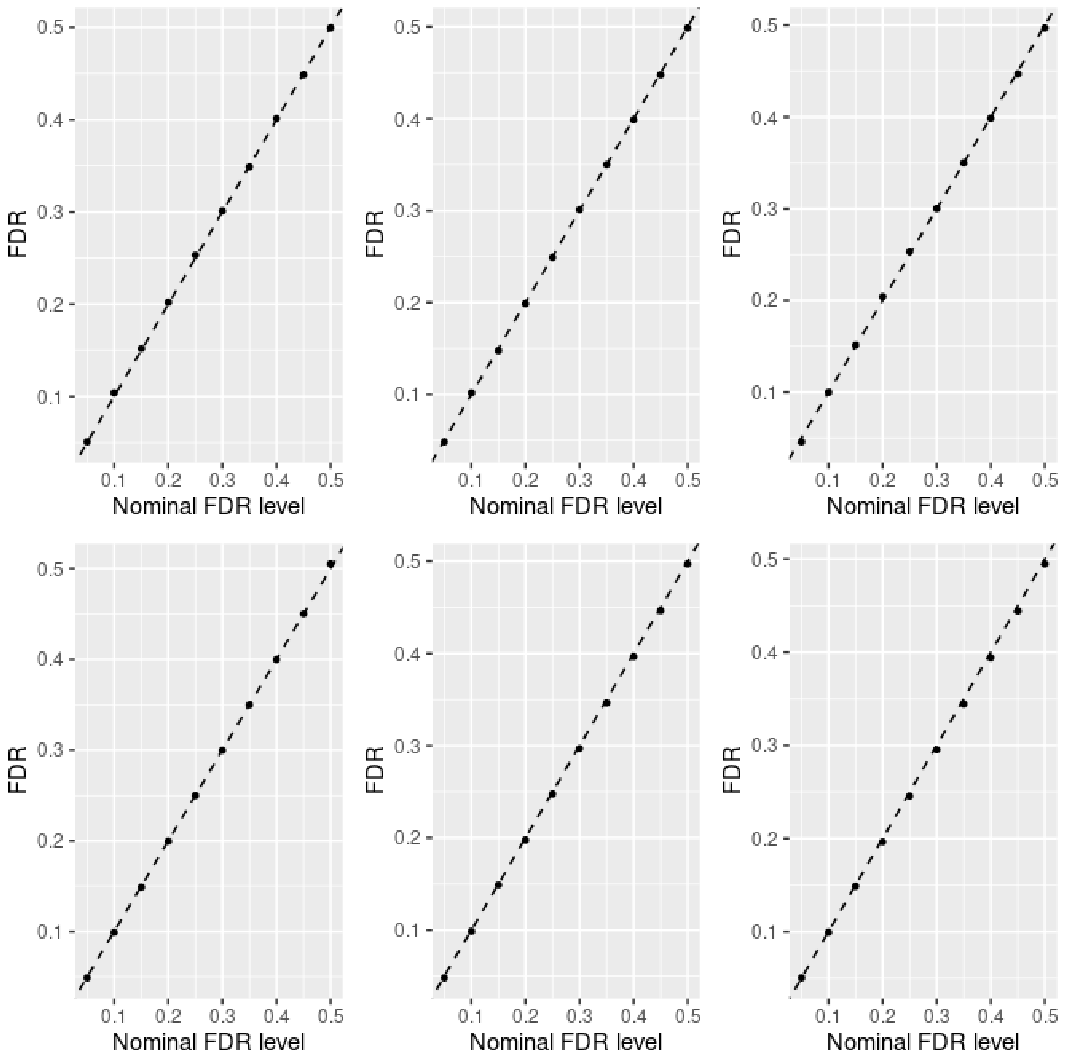

Figure S21: Simulation study with Γ_3 without using annotation data: False discovery rate control.

	P1	P2	P3	P4	P5	P6
P1	2936	2013	890	1000	993	373
P2	2013	5212	1387	1701	1666	653
P3	890	1387	2451	1837	1688	464
P4	1000	1701	1837	31593	29867	7648
P5	993	1666	1688	29867	37502	9329
P6	373	653	464	7648	9329	31473

Table S5. Simulation study with Γ_3 using annotation data: Numbers of SNPs identified to be associated with each pair of phenotypes with the global FDR at nominal level of 5%. Diagonal elements show the number of SNPs inferred to be associated with each phenotype when the global FDR is controlled at the same level.

	P1	P2	P3	P4	P5	P6
P1	2936	2012	890	974	953	274
P2	2012	5211	1385	1641	1597	470
P3	890	1385	2443	1802	1633	340
P4	974	1641	1802	30671	28977	5426
P5	953	1597	1633	28977	35985	6839
P6	274	470	340	5426	6839	24805

Table S6. Simulation study with Γ_3 without using annotation data: Numbers of SNPs identified to be associated with each pair of phenotypes with the global FDR at nominal level of 5%. Diagonal elements show the number of SNPs inferred to be associated with each phenotype when the global FDR is controlled at the same level.

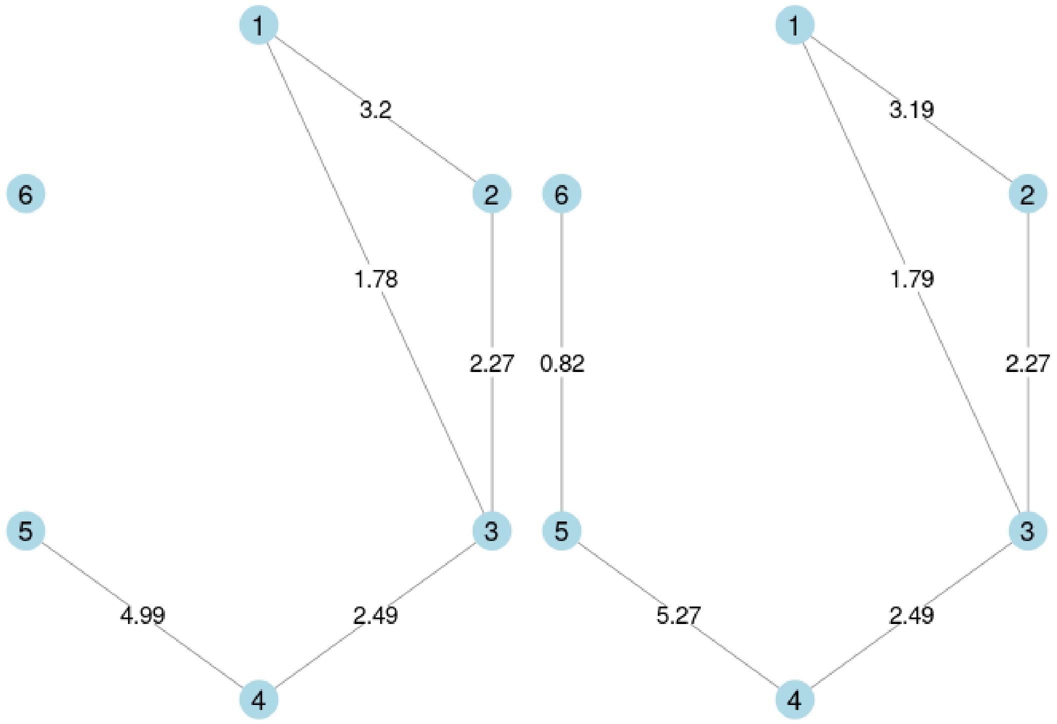

Figure S22: Simulation study with Γ_3: Phenotype graphs estimated using annotation data (left) and without using annotation data (right).

3 REAL DATA ANALYSIS

3.1 Autoimmune Disease GWAS Data Analysis

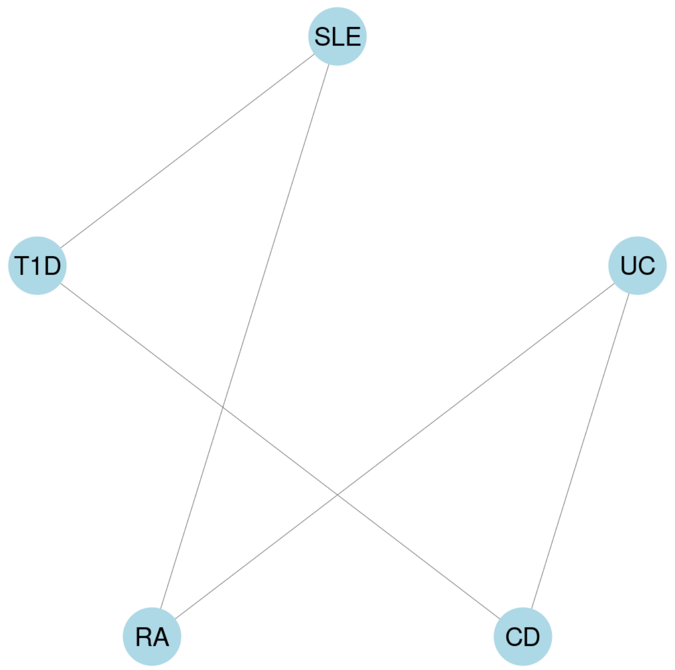

Figure S23: Prior disease graph obtained by biomedical literature mining for autoimmune diseases (Kim et al., 2018).

3.1.1 Integration with GenoSkyline

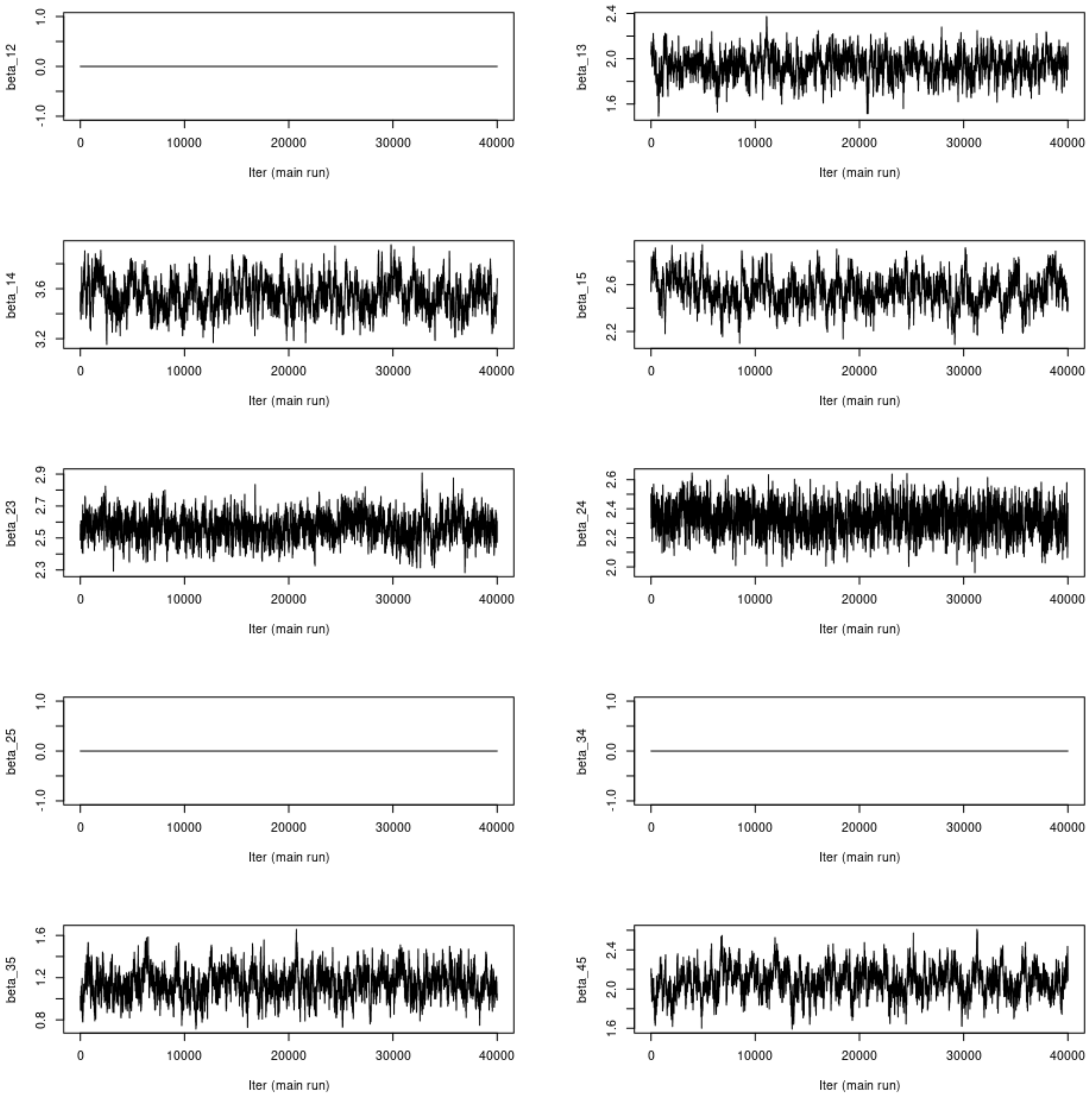

Figure S24: GGPA 2.0 analysis of autoimmune diseases using annotations of GenoSkyline. Trace plot of β.

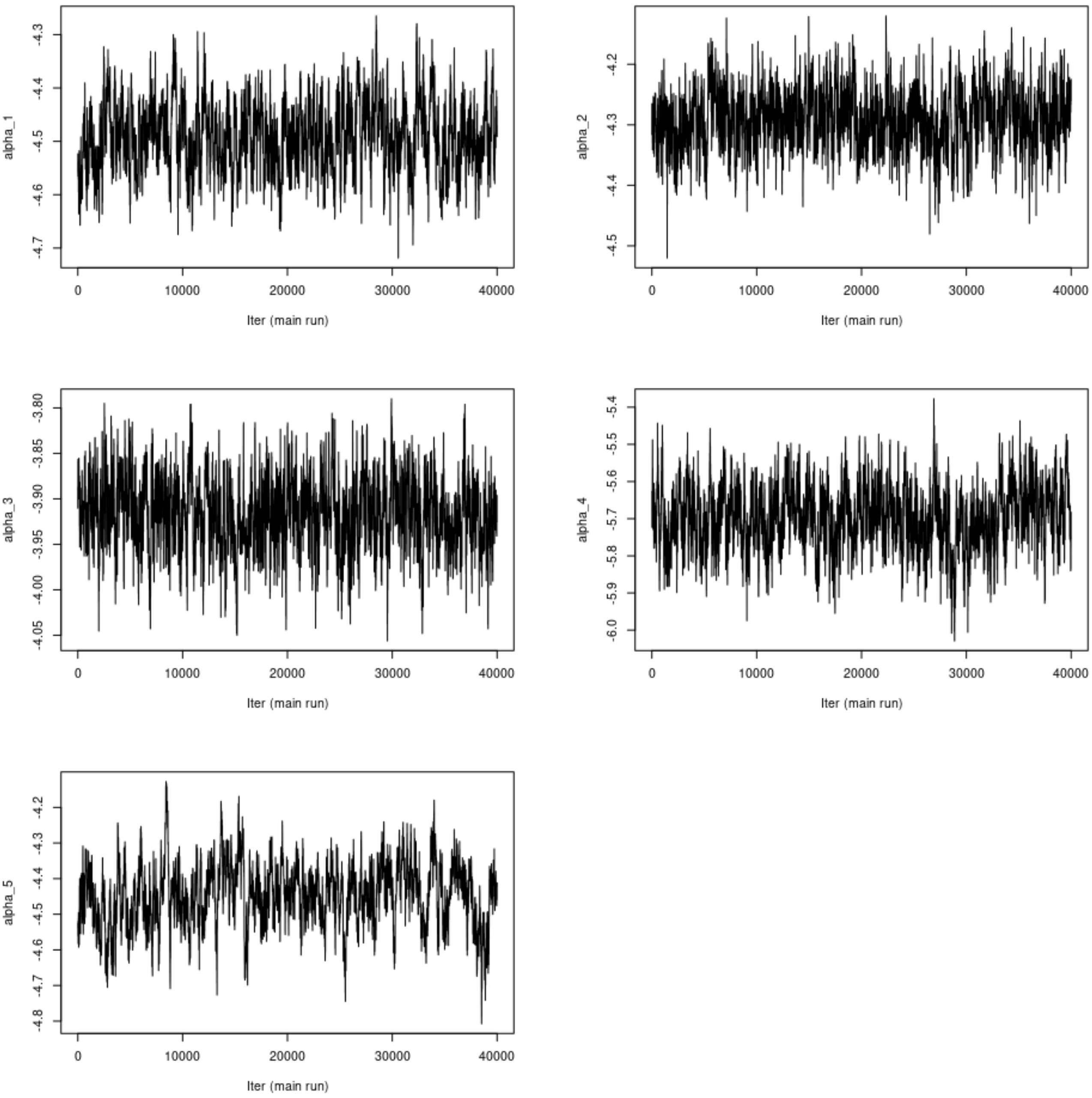

Figure S25: GGPA 2.0 analysis of autoimmune diseases using annotations of GenoSkyline. Trace plot of α.

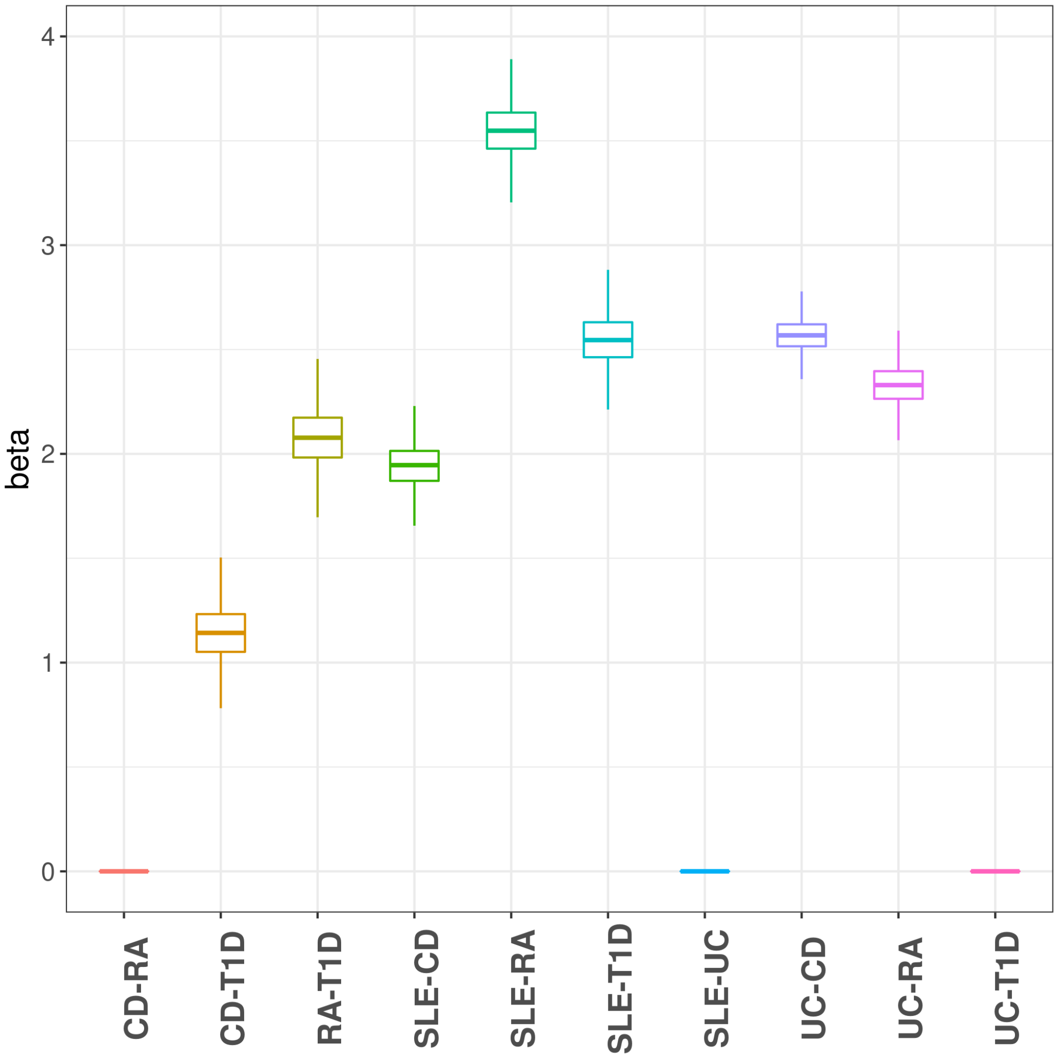

Figure S26: GGPA 2.0 analysis of autoimmune diseases using annotations of GenoSkyline. Coefficient estimates of β suggest a strong pleiotropy between SLE and RA.

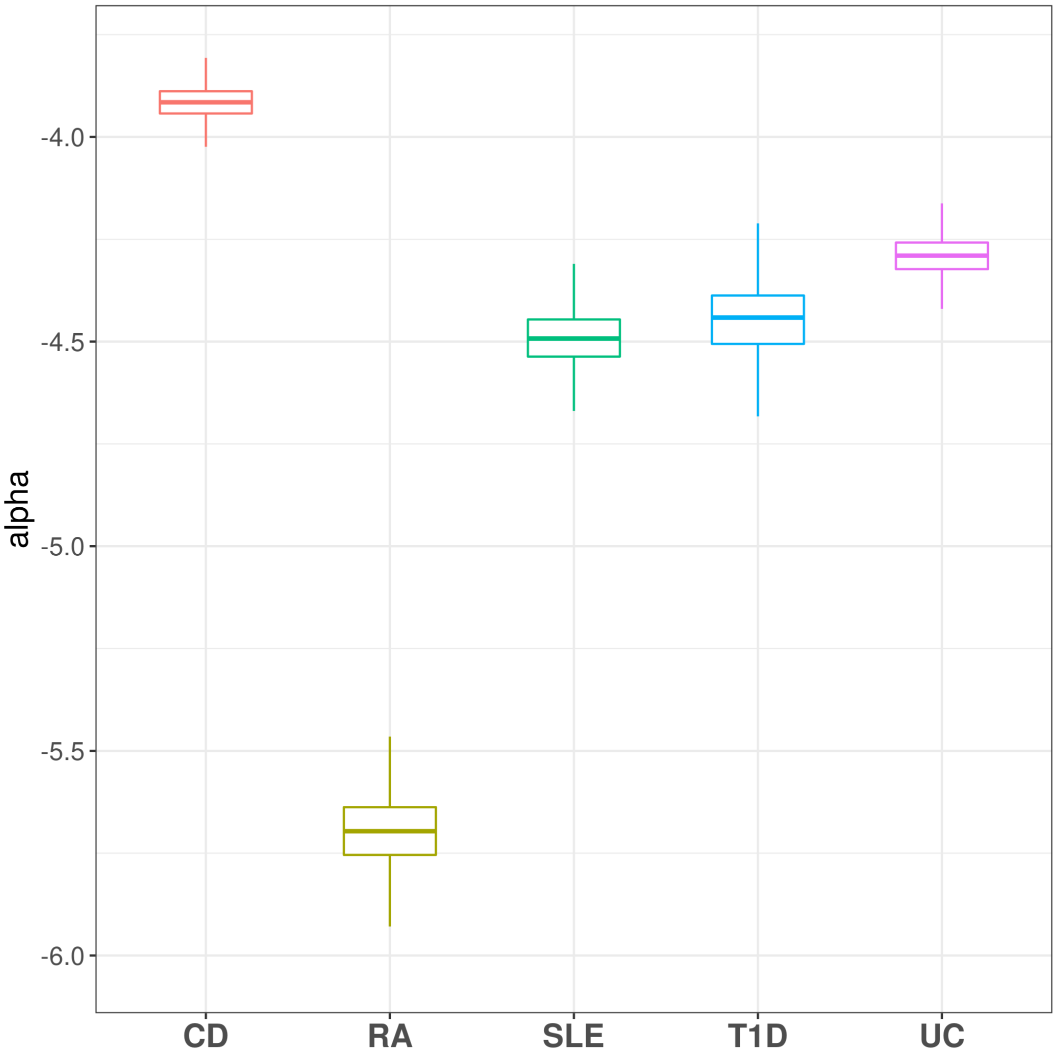

Figure S27: GGPA 2.0 analysis of autoimmune diseases using annotations of GenoSkyline. Coefficient estimates of α suggest a stronger genetic basis of CD compared with other autoimmune diseases.

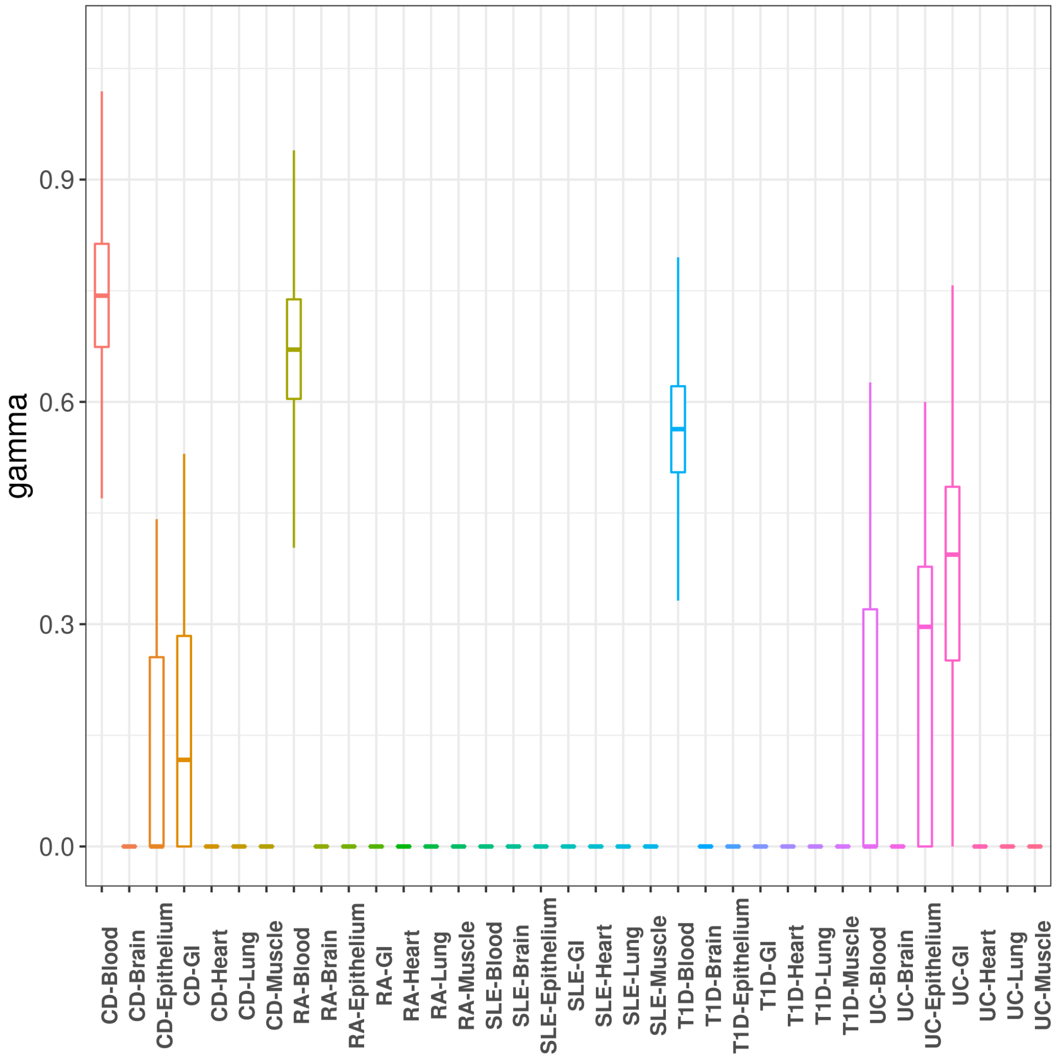

Figure S28: GGPA 2.0 analysis of autoimmune diseases using annotations of Genoskyline. The coefficient estimates of γ show that blood and brain are associated with autoimmune diseases.

	SLE	UC	CD	RA	T1D
SLE	1671	383	613	945	935
UC	383	1103	546	423	325
CD	613	546	1918	476	478
RA	945	423	476	1294	764
T1D	935	325	478	764	1358

Table S7. GGPA 2.0 analysis of autoimmune diseases using annotations of GenoSkyline: Numbers of SNPs identified to be associated with each pair of phenotypes with the global FDR at nominal level of 5%. Diagonal elements show the number of SNPs inferred to be associated with each phenotype when the global FDR is controlled at the same level.

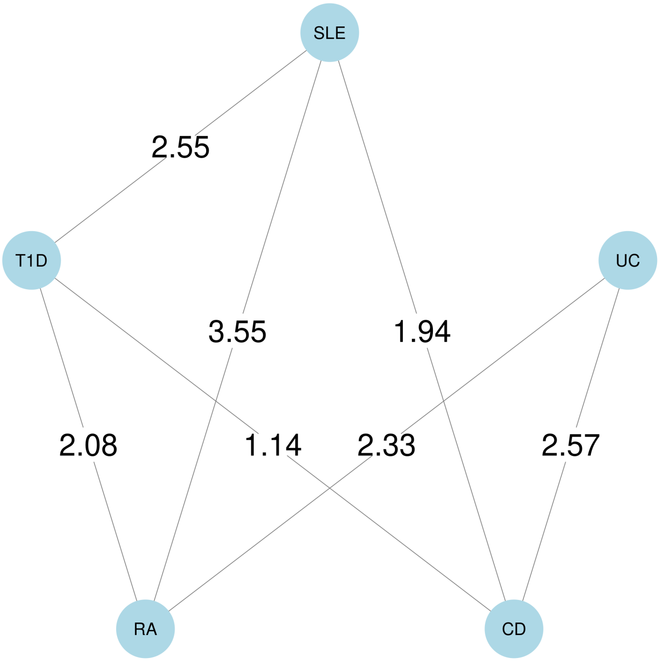

Figure S29: GGPA 2.0 analysis of autoimmune diseases using annotations of GenoSkyline. Estimated phenotype graph for autoimmune diseases. Values on the edges show β coefficient estimates.

3.1.2 Integration with Genoskyline-Plus

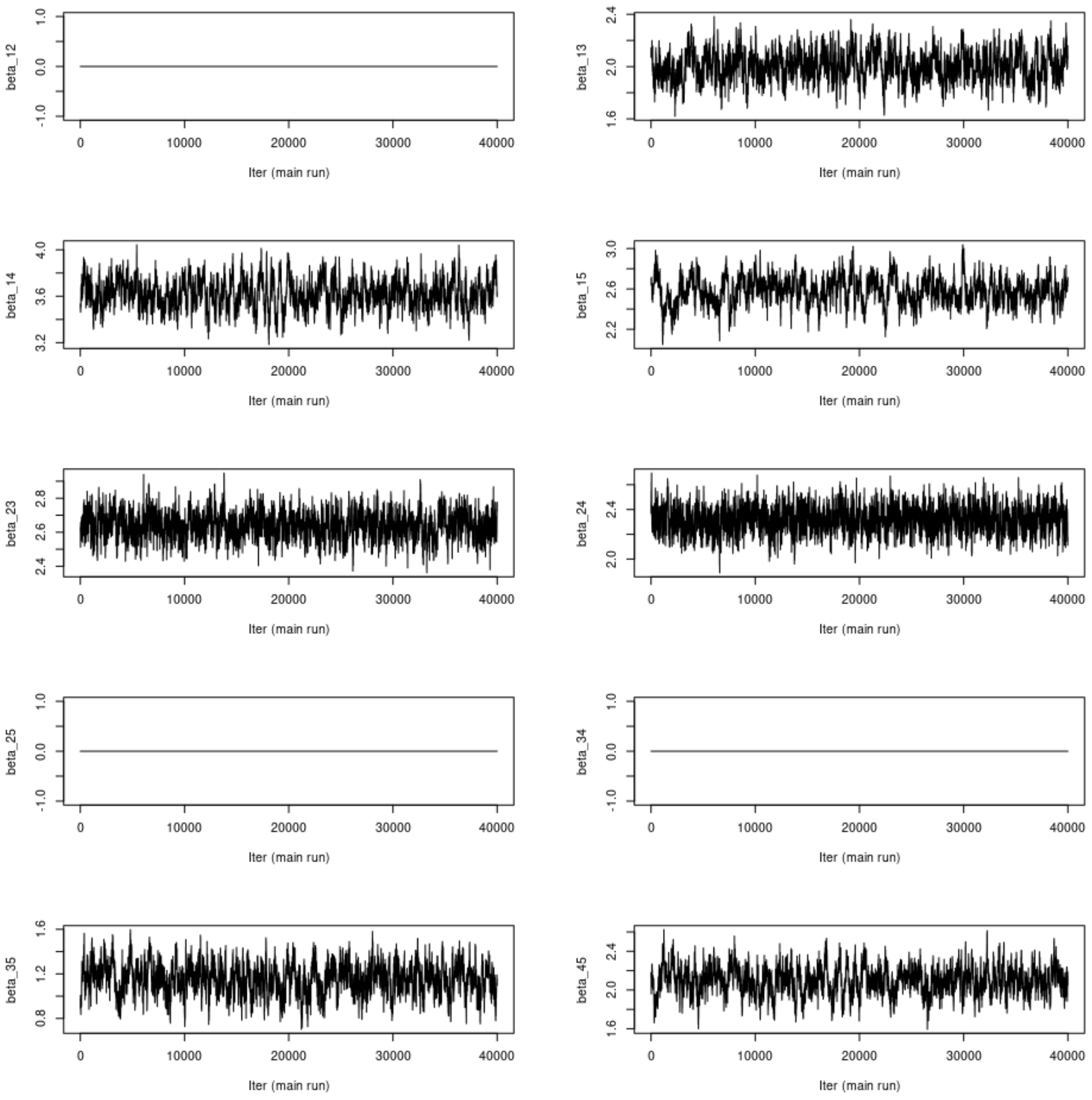

Figure S30: GGPA 2.0 analysis of autoimmune diseases using annotations of GenoSkyline-Plus. Trace plot of β.

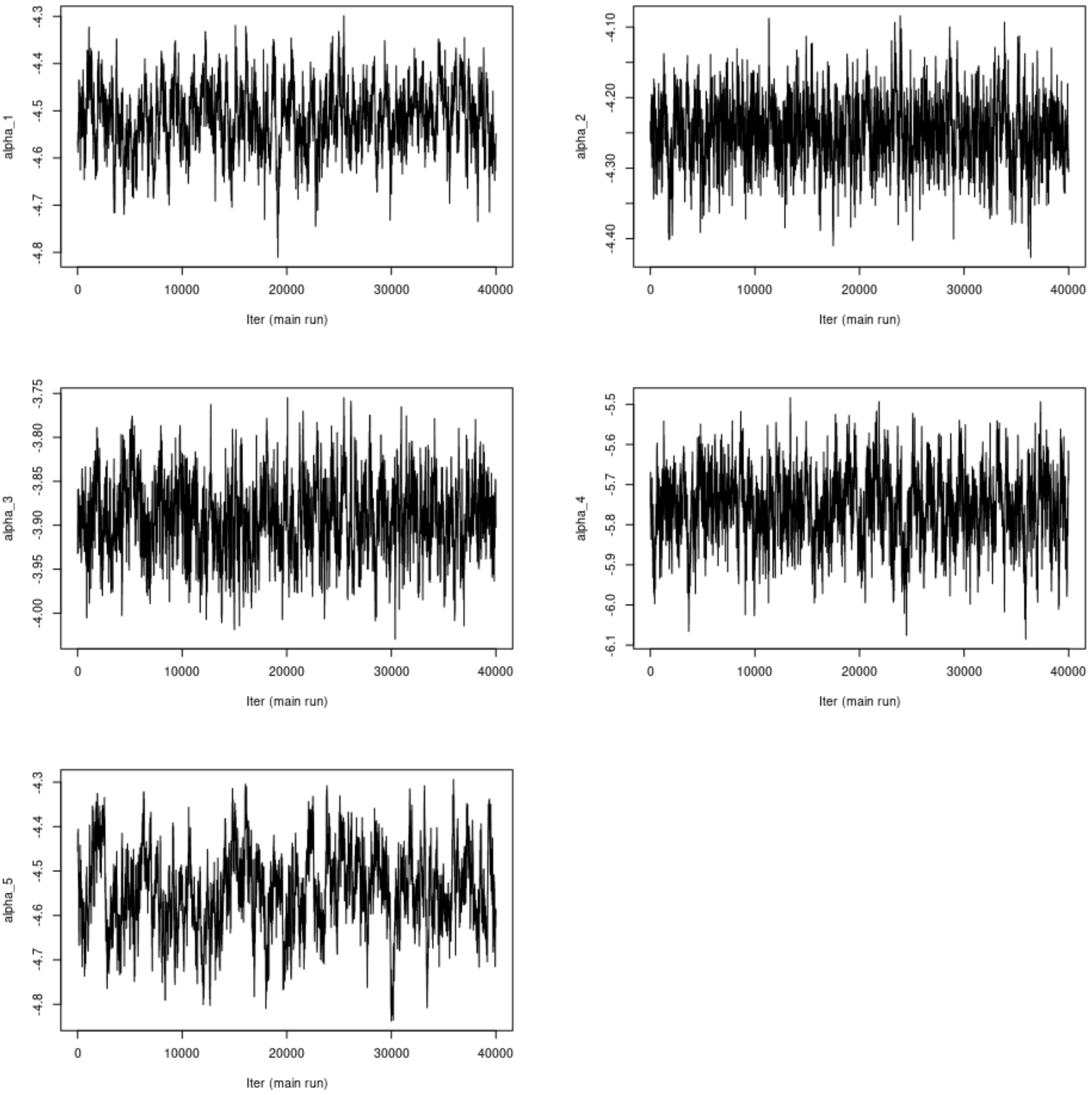

Figure S31: GGPA 2.0 analysis of autoimmune diseases using annotations of GenoSkyline-Plus. Trace plot of α.

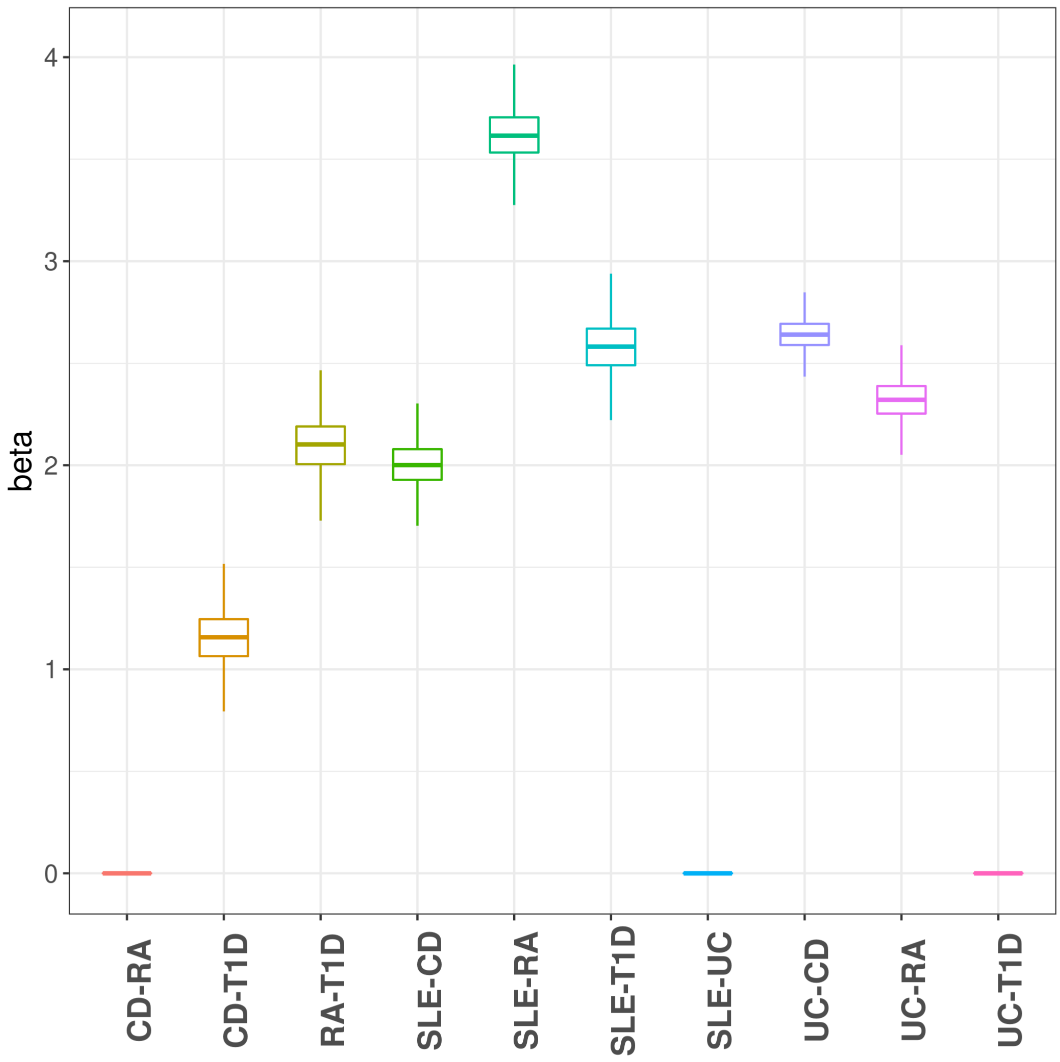

Figure S32: GGPA 2.0 analysis of autoimmune diseases using annotations of GenoSkyline-Plus. Coefficient estimates of β suggest a strong pleiotropy between SLE and RA.

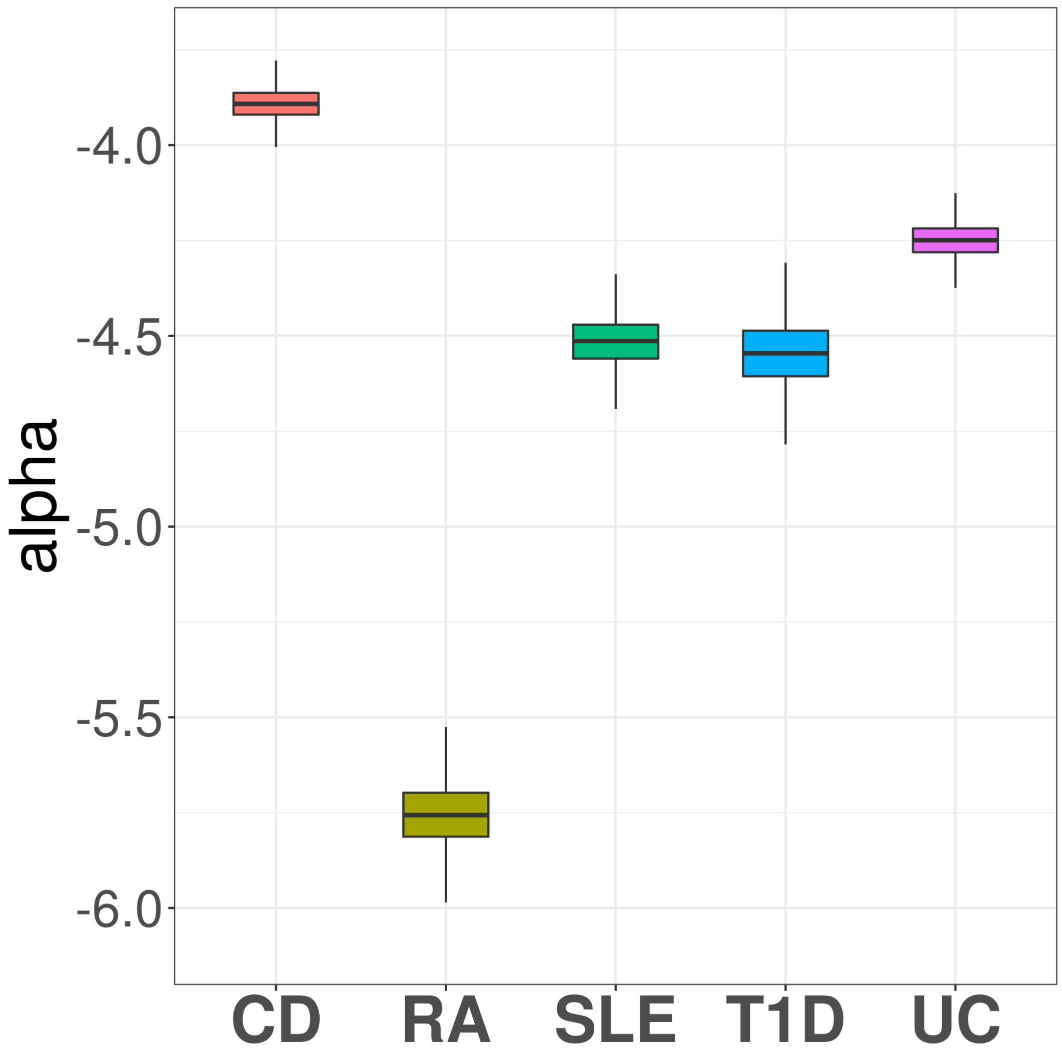

Figure S33: GGPA 2.0 analysis of autoimmune diseases using annotations of GenoSkyline-Plus. Coefficient estimates of α suggest a stronger genetic basis of CD compared with other autoimmune diseases.

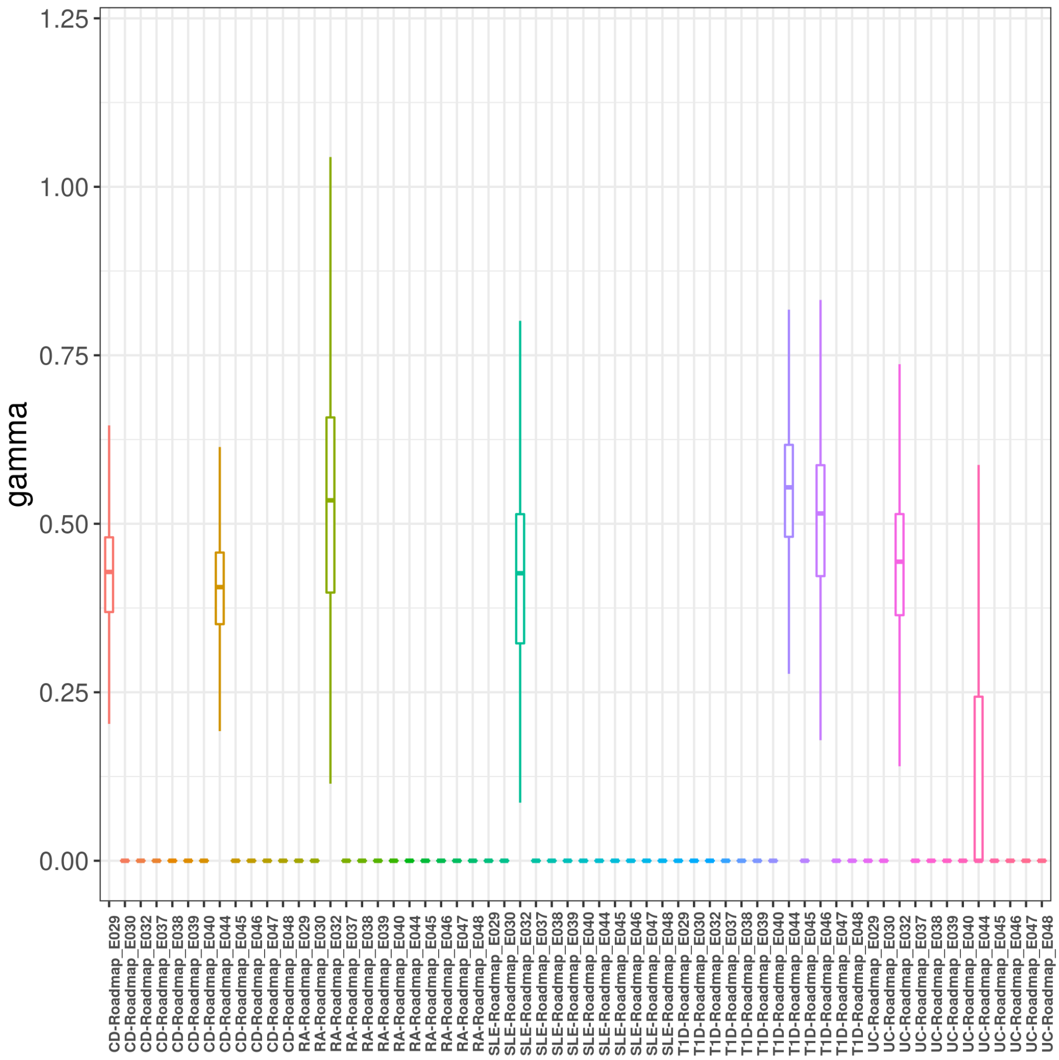

Figure S34: GGPA 2.0 analysis of autoimmune diseases using annotations of GenoSkyline-Plus. Coefficient estimates of γ show that B cells and regulatory T cells are associated with autoimmune diseases. Roadmap E029: Primary monocytes; Roadmap E030: Primary neutrophils; Roadmap E032: Primary B cells; Roadmap E037: Primary T helper memory cells 1; Roadmap E038: Primary T helper naive cells 1; Roadmap E039: Primary T helper naive cells 2; Roadmap E040: Primary T helper memory cells 2; Roadmap E044: Primary T regulatory cells; Roadmap E045: Primary T cells effect/memory; Roadmap E046: Primary natural killer cells; Roadmap E047: Primary T CD8+ naive cells; and Roadmap E048: Primary T CD8+ memory cells.

	SLE	UC	CD	RA	T1D
SLE	1680	392	628	945	941
UC	392	1100	549	421	331
CD	628	549	1898	474	486
RA	945	421	474	1268	755
T1D	941	331	486	755	1353

Table S8. GGPA 2.0 analysis of autoimmune diseases using annotations of GenoSkyline-Plus: Numbers of SNPs identified to be associated with each pair of phenotypes with the global FDR at nominal level of 5%. Diagonal elements show the number of SNPs inferred to be associated with each phenotype when the global FDR is controlled at the same level.

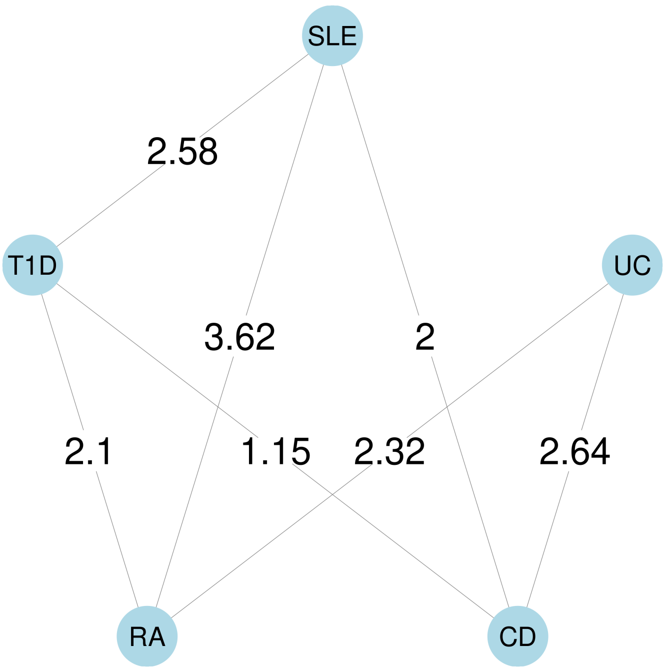

Figure S35: GGPA 2.0 analysis of autoimmune diseases using annotations of GenoSkyline-Plus. Estimated phenotype graph of autoimmune diseases. Values on the edges show β coefficient estimates.

3.1.3 Analysis without Using Functional Annotation

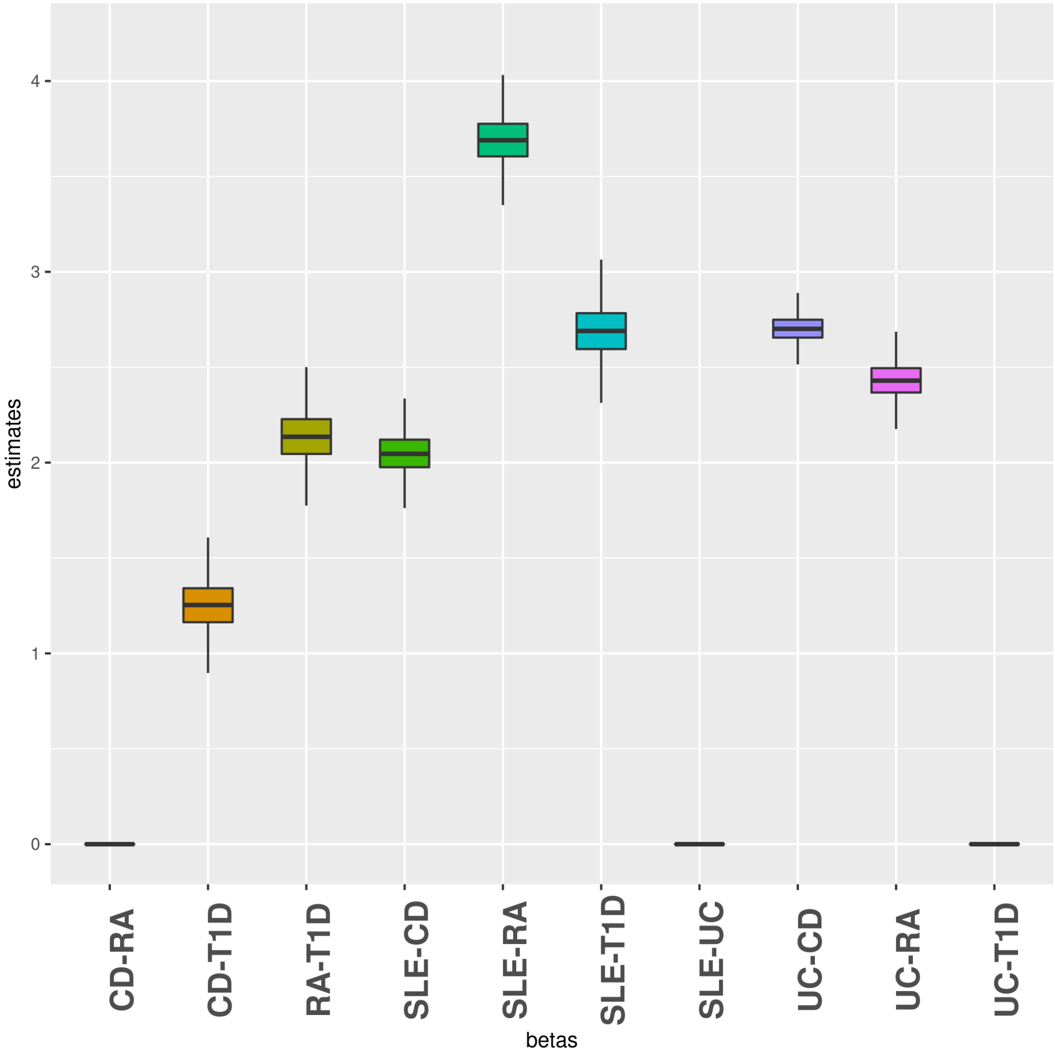

Figure S36: GGPA 2.0 analysis of autoimmune diseases without using functional annotation. Coefficient estimates of β suggest a strong pleiotropy between SLE and RA.

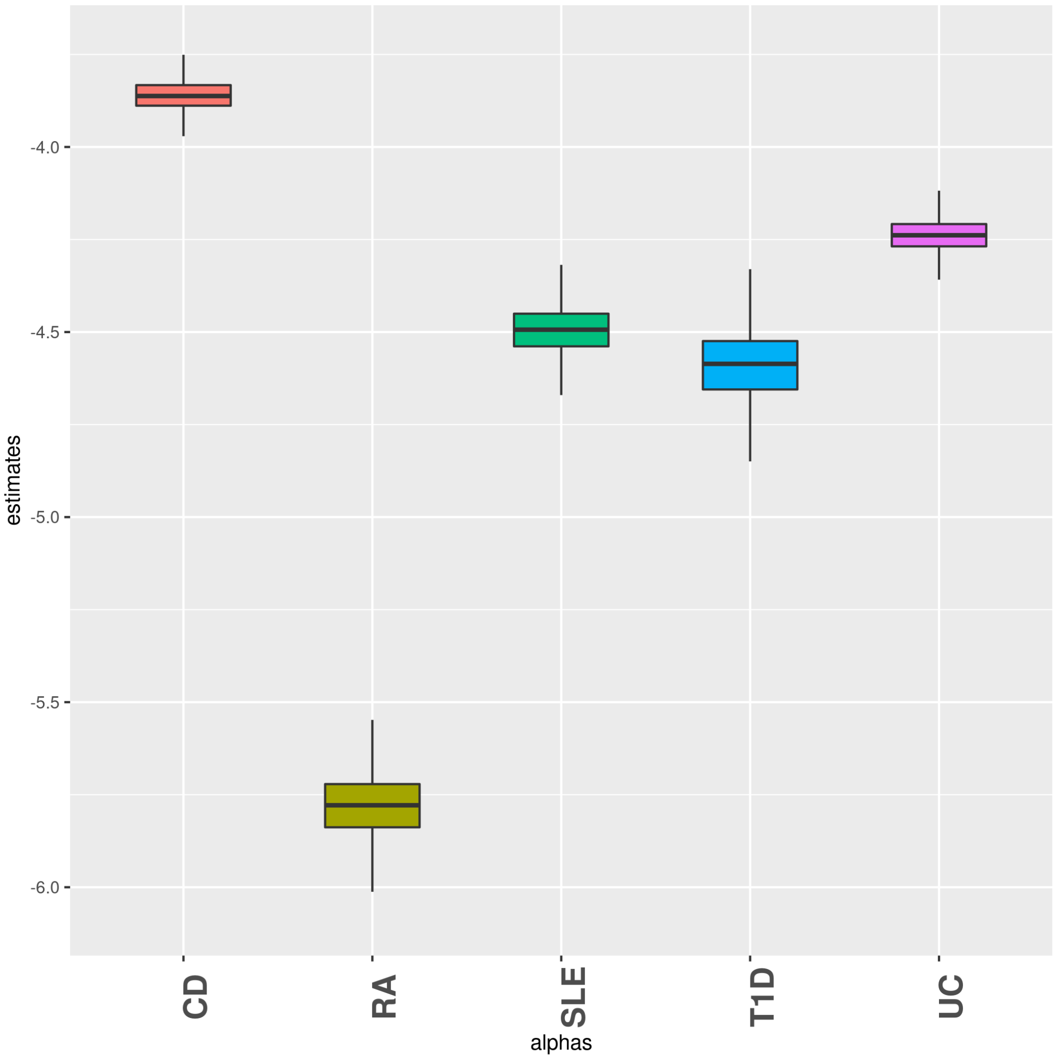

Figure S37: GGPA 2.0 analysis of autoimmune diseases without using functional annotation. Coefficient estimates of α suggest a stronger genetic basis of CD compared with other autoimmune diseases.

	SLE	UC	CD	RA	T1D
SLE	1670	391	623	937	926
UC	391	1092	546	422	327
CD	623	546	1872	471	476
RA	937	422	471	1254	736
T1D	926	327	476	736	1310

Table S9. GGPA 2.0 analysis of autoimmune diseases without using functional annotation: Numbers of SNPs identified to be associated with each pair of phenotypes with the global FDR at nominal level of 5%. Diagonal elements show the number of SNPs inferred to be associated with each phenotype when the global FDR is controlled at the same level.

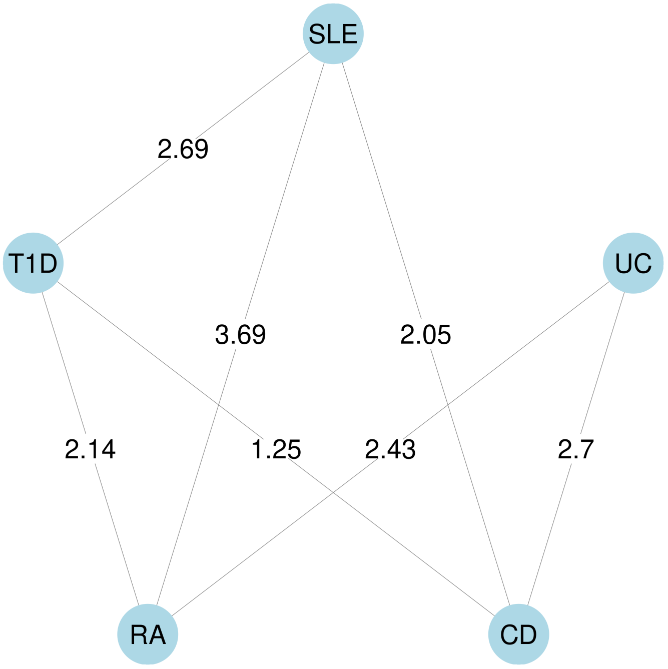

Figure S38: GGPA 2.0 analysis of autoimmune diseases without using functional annotation. Estimated phenotype graph of autoimmune diseases. Values on the edges show β coefficient estimates.

3.1.4 Analysis without Using Prior Disease Graph

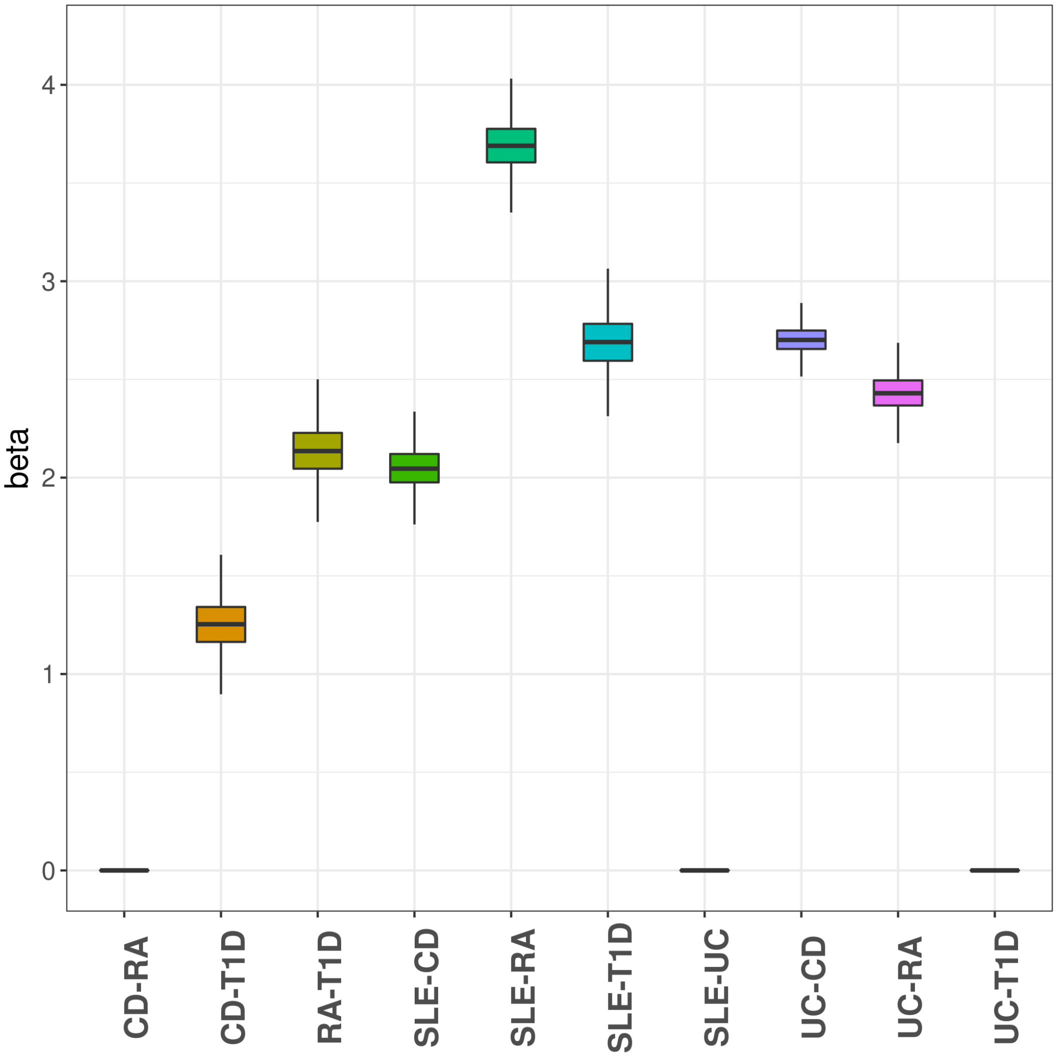

Figure S39: GGPA 2.0 analysis of autoimmune diseases using annotations of GenoSkyline-Plus, but without using the prior disease graph. Coefficient estimates of β suggest a strong pleiotropy between SLE and RA.

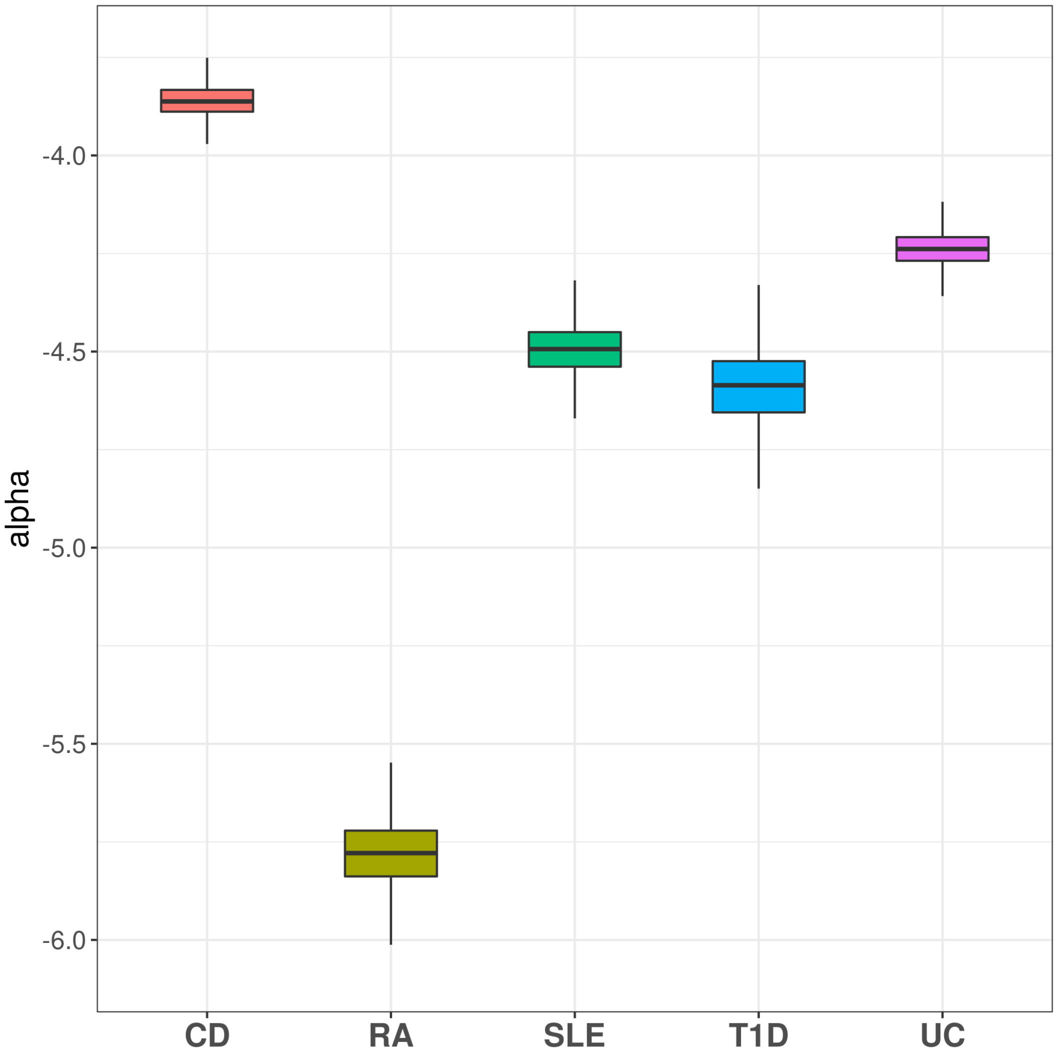

Figure S40: GGPA 2.0 analysis of autoimmune diseases using annotations of GenoSkyline-Plus, but without using the prior disease graph. Coefficient estimates of α suggest a stronger genetic basis of CD compared with other autoimmune diseases.

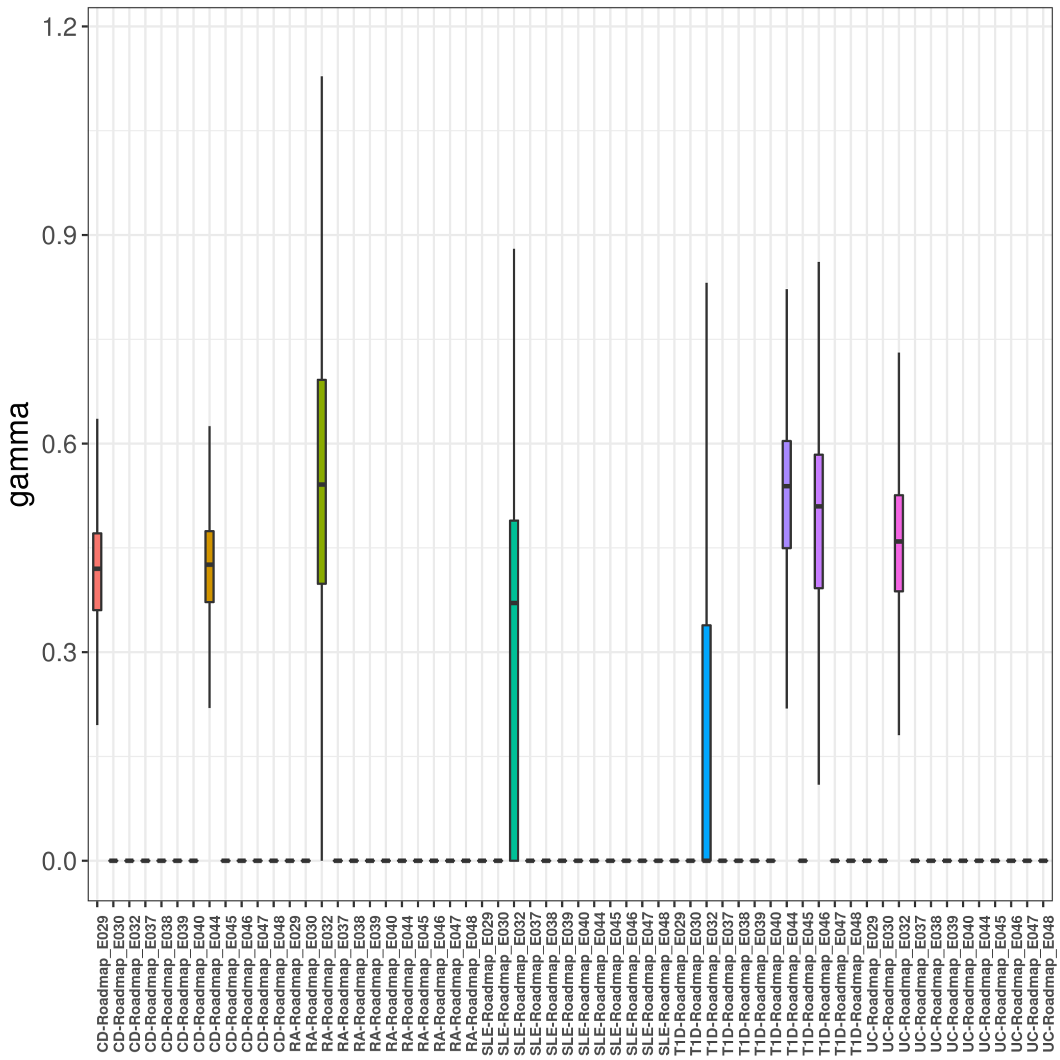

Figure S41: GGPA 2.0 analysis of autoimmune diseases using annotations of GenoSkyline-Plus, but without using the prior disease graph. Coefficient estimates of γ show that B cells and regulatory T cells are associated with autoimmune diseases. Roadmap E029: Primary monocytes; Roadmap E030: Primary neutrophils; Roadmap E032: Primary B cells; Roadmap E037: Primary T helper memory cells 1; Roadmap E038: Primary T helper naive cells 1; Roadmap E039: Primary T helper naive cells 2; Roadmap E040: Primary T helper memory cells 2; Roadmap E044: Primary T regulatory cells; Roadmap E045: Primary T cells effect/memory; Roadmap E046: Primary natural killer cells; Roadmap E047: Primary T CD8+ naive cells; and Roadmap E048: Primary T CD8+ memory cells.

	SLE	UC	CD	RA	T1D
SLE	1680	392	629	945	939
UC	392	1100	549	421	330
CD	629	549	1899	474	484
RA	945	421	474	1269	755
T1D	939	330	484	755	1351

Table S10. GGPA 2.0 analysis of autoimmune diseases using annotations of GenoSkyline-Plus, but without using the prior disease graph: Numbers of SNPs identified to be associated with each pair of phenotypes with the global FDR at nominal level of 5%. Diagonal elements show the number of SNPs inferred to be associated with each phenotype when the global FDR is controlled at the same level.

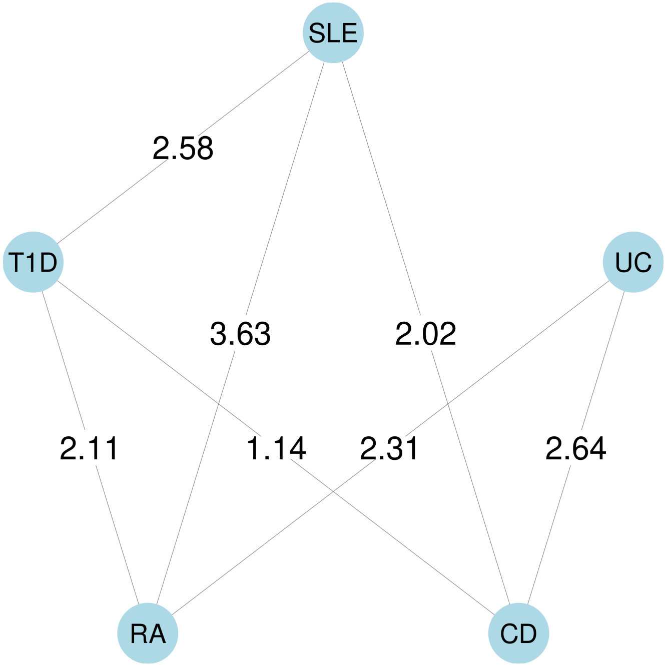

Figure S42: GGPA 2.0 analysis of autoimmune diseases using annotations of GenoSkyline-Plus, but without using the prior disease graph. Estimated phenotype graph of autoimmune diseases. Values on the edges show β coefficient estimates.

3.2 Psychiatric Disorder GWAS Data Analysis

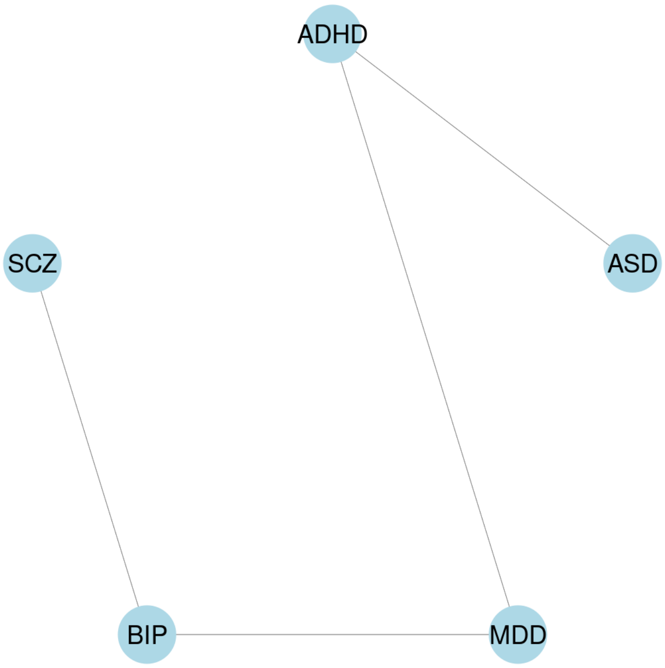

Figure S43: Prior disease graph obtained by biomedical literature mining for psychiatric disorders (Kim et al., 2018).

3.2.1 Integration with Genoskyline

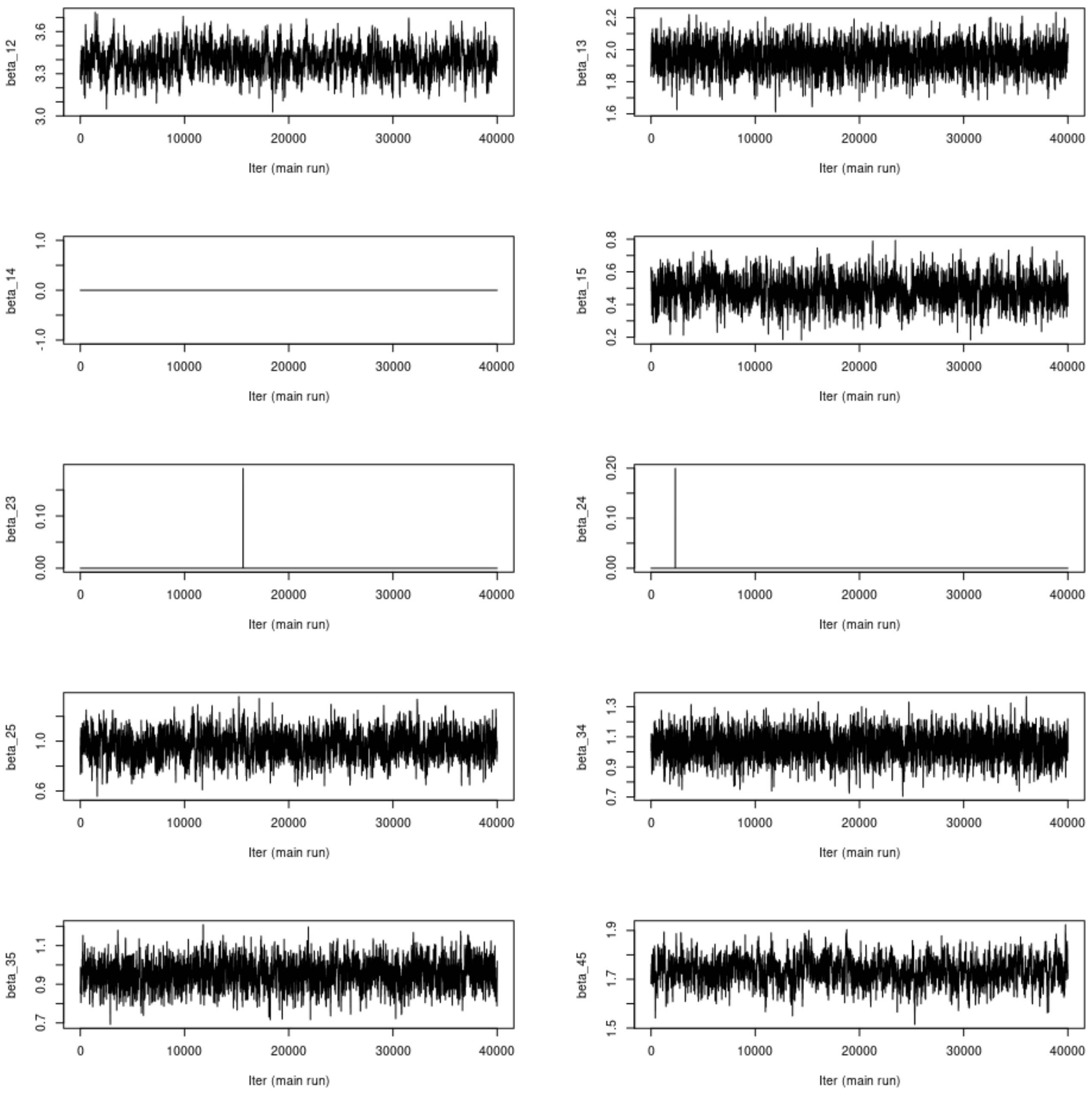

Figure S44: GGPA 2.0 analysis of psychiatric disorders using annotations of GenoSkyline. Trace plot of β.

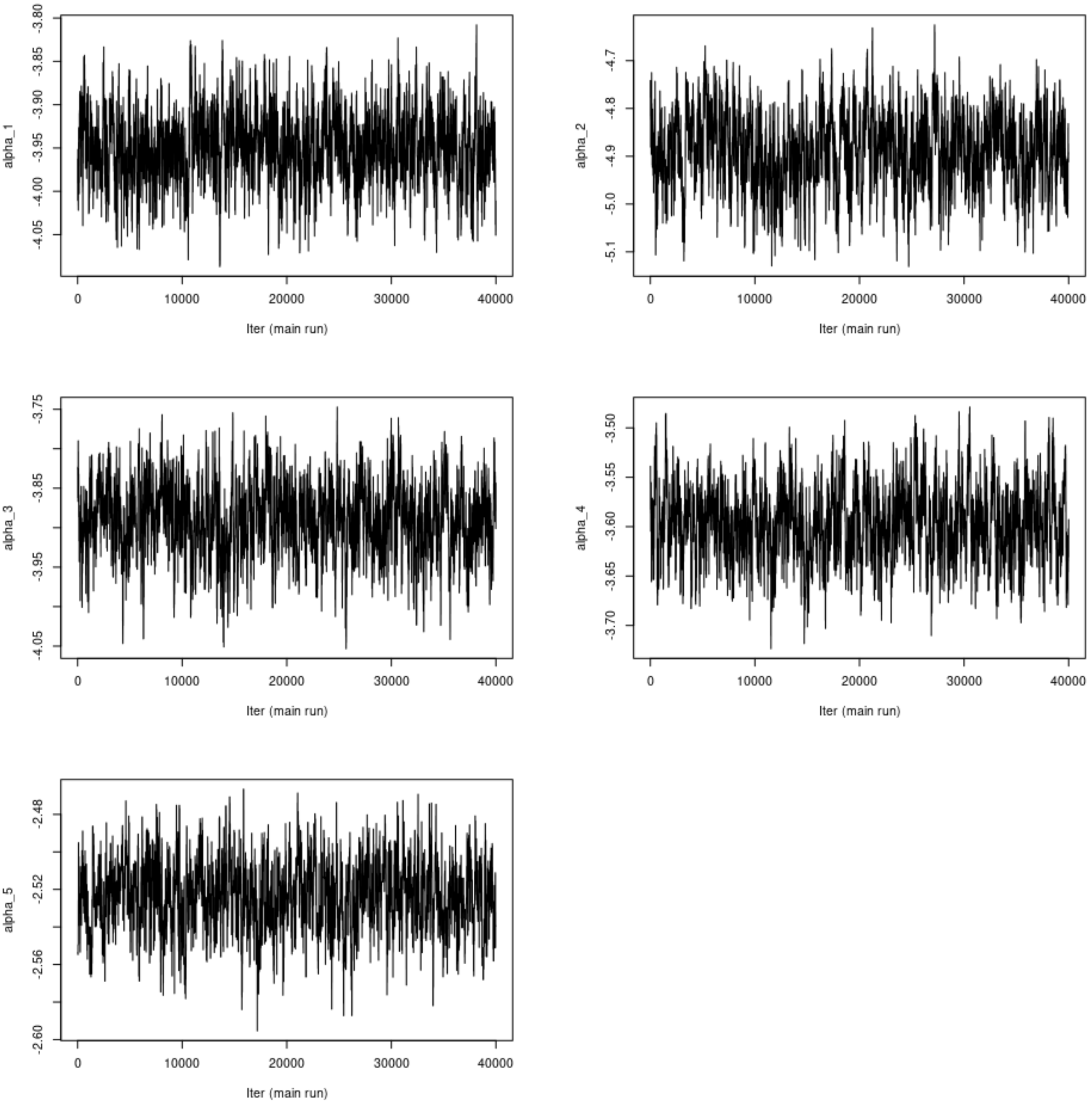

Figure S45: GGPA 2.0 analysis of psychiatric disorders using annotations of GenoSkyline. Trace plot of α.

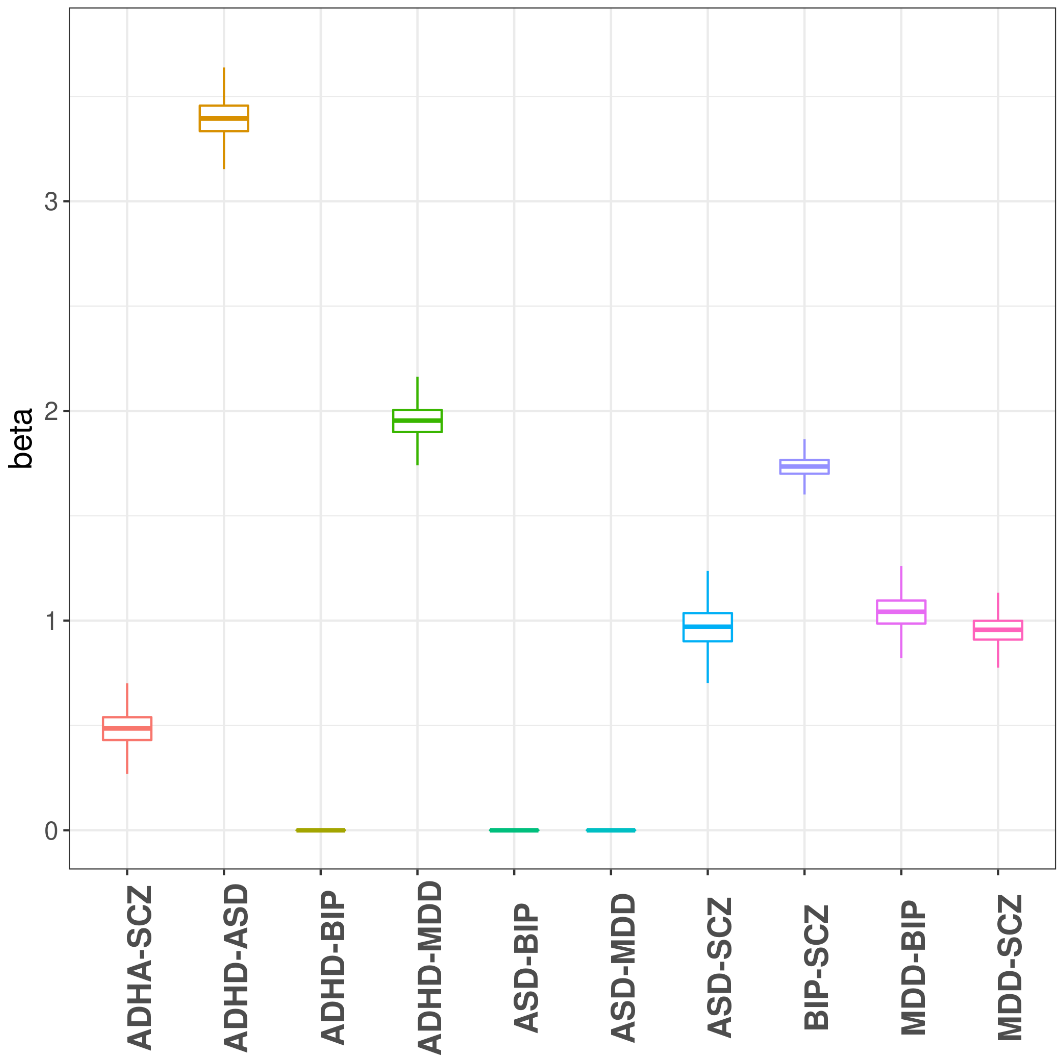

Figure S46: GGPA 2.0 analysis of psychiatric disorders using annotations of GenoSkyline. Coefficient estimates of β suggest a strong pleiotropy between ADHD and ASD.

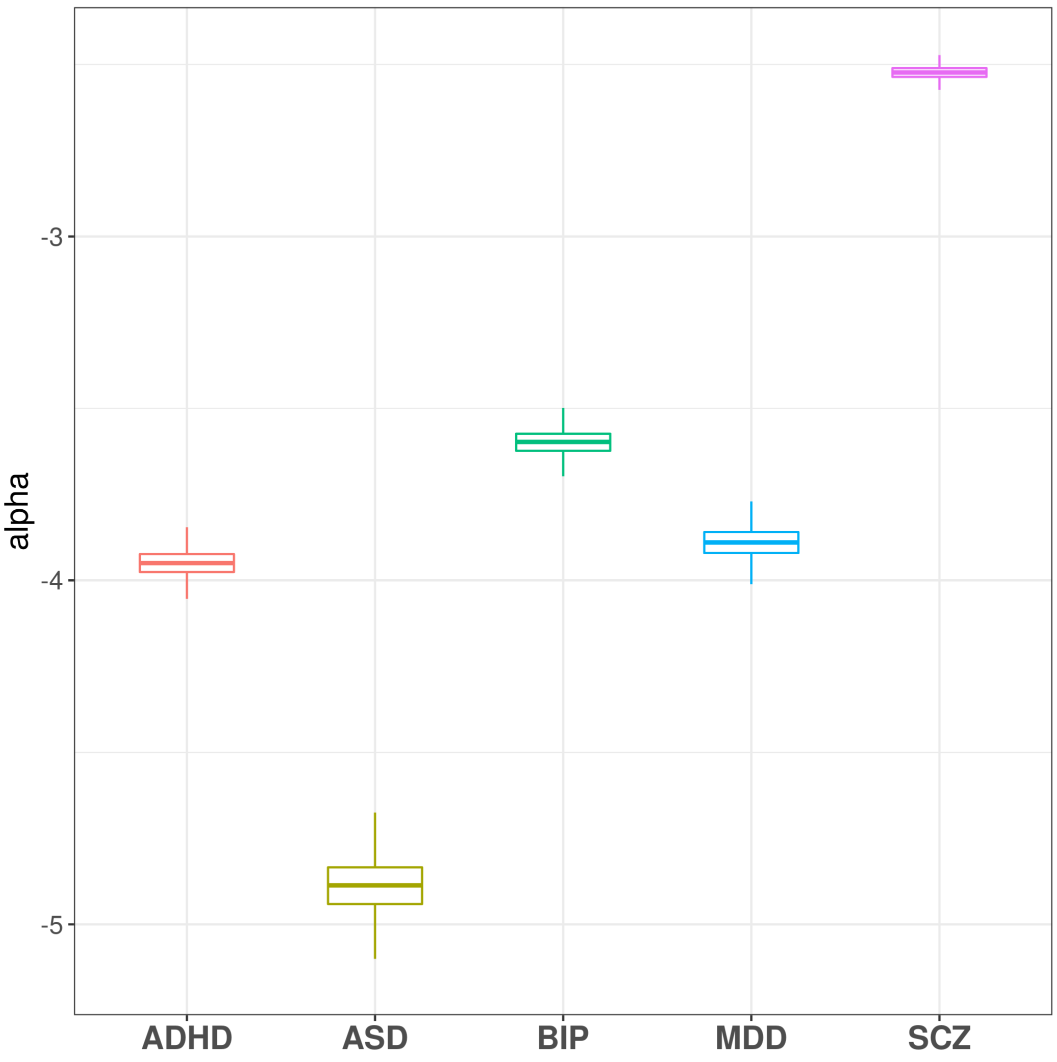

Figure S47: GGPA 2.0 analysis of psychiatric disorders using annotations of GenoSkyline. Coefficient estimates of α suggest a stronger genetic basis of SCZ compared with other psychiatric disorders.

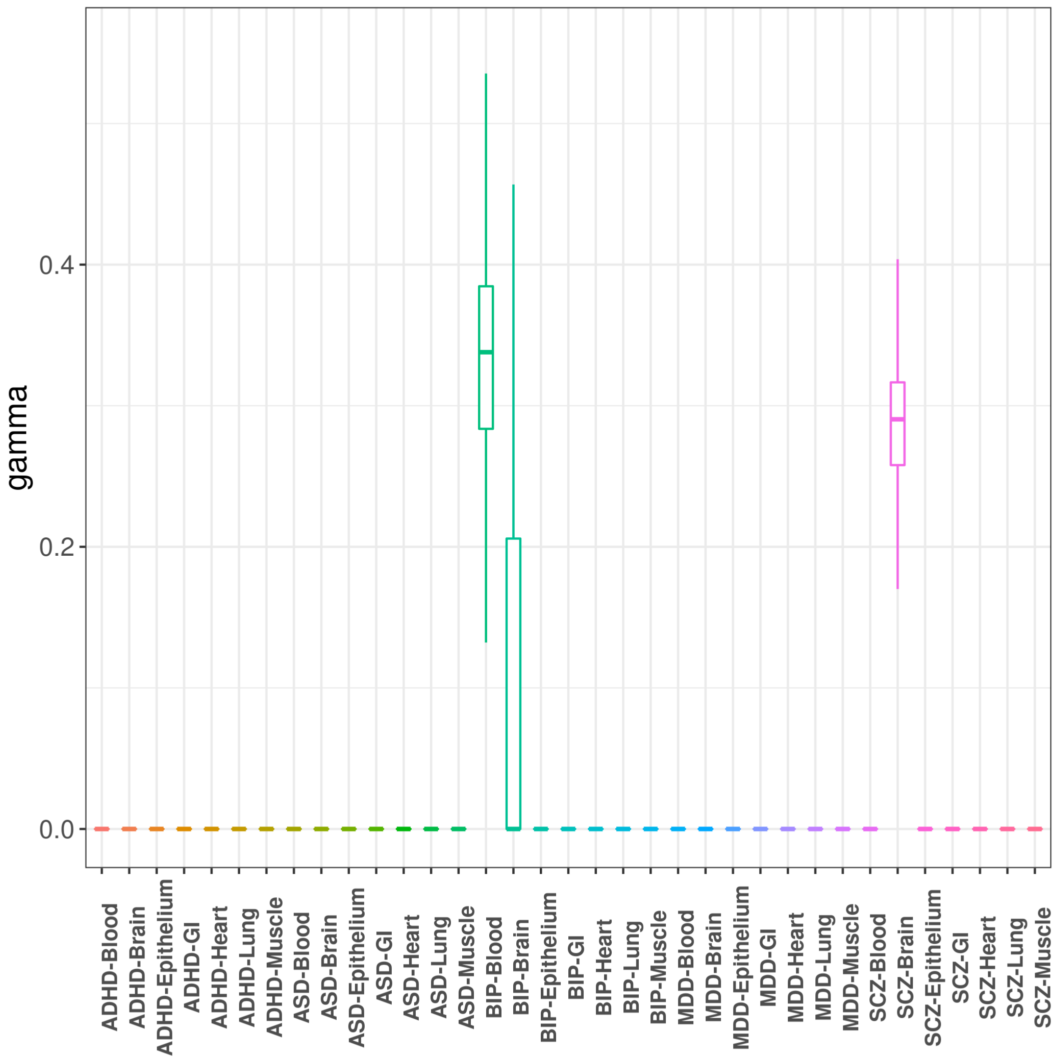

Figure S48: GGPA 2.0 analysis of psychiatric disorders using annotations of GenoSkyline. Coefficient estimates of γ show that blood is associated with BIP and brain is associated with SCZ.

	ADHD	ASD	MDD	BIP	SCZ
ADHD	356	65	50	6	88
ASD	65	210	18	0	74
MDD	50	18	342	5	194
BIP	6	0	5	561	262
SCZ	88	74	194	262	3961

Table S11. GGPA 2.0 analysis of psychiatric disorders using annotations of GenoSkyline: Numbers of SNPs identified to be associated with each pair of phenotypes with the global FDR at nominal level of 5%. Diagonal elements show the number of SNPs inferred to be associated with each phenotype when the global FDR is controlled at the same level.

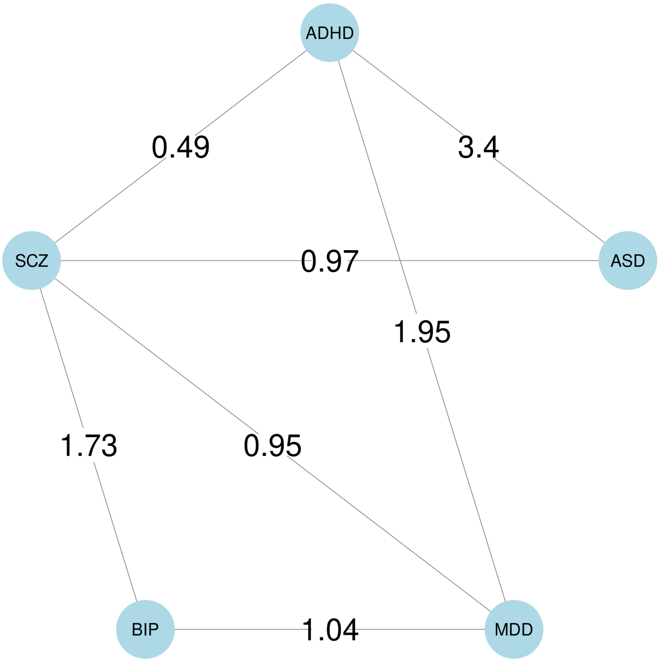

Figure S49: GGPA 2.0 analysis of psychiatric disorders using annotations of GenoSkyline. Estimated phenotype graph of psychiatric disorders. Values on the edges show β coefficient estimates.

3.2.2 Integration with Genoskyline-Plus

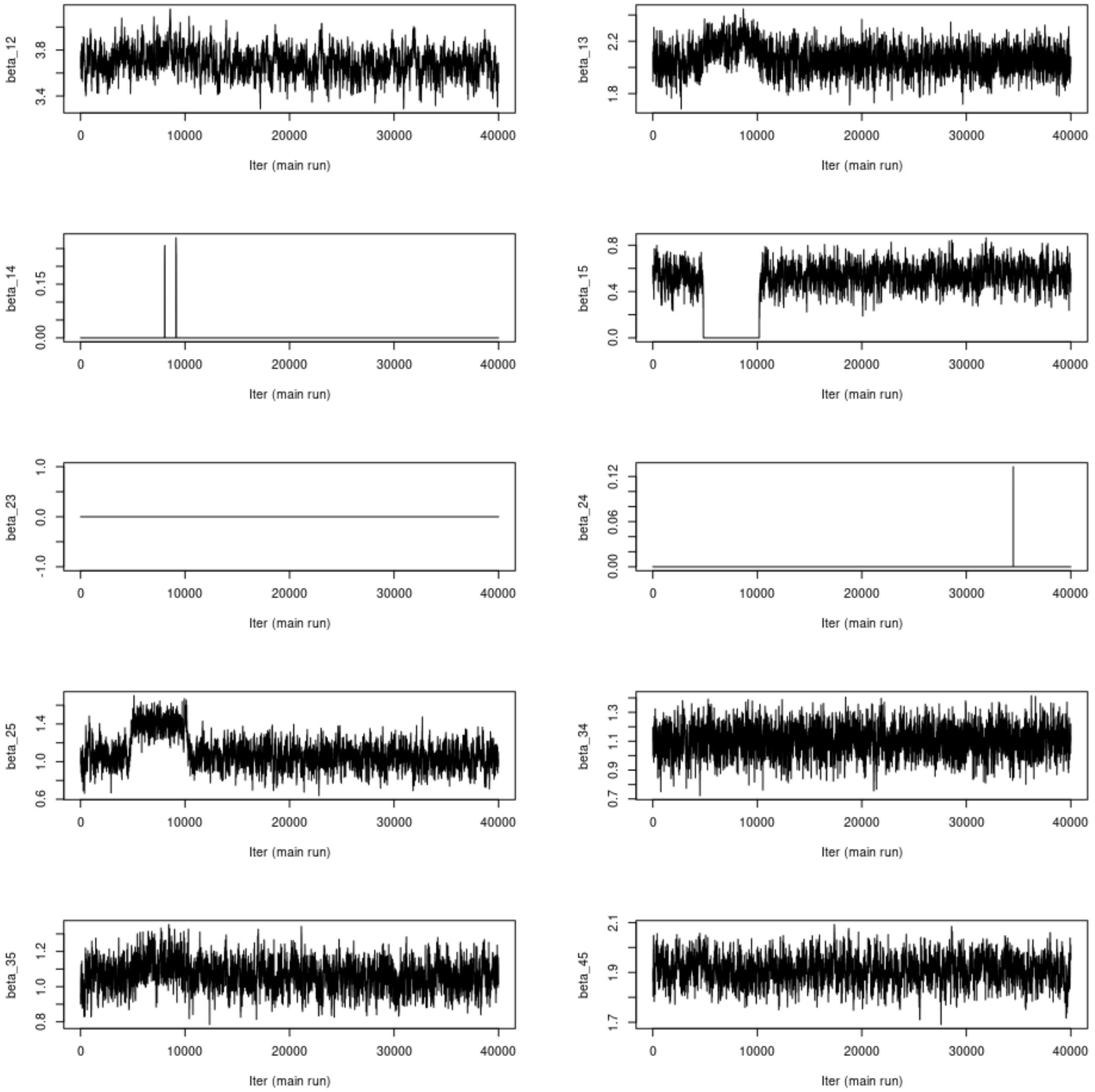

Figure S50: GGPA 2.0 analysis of psychiatric disorders using annotations of GenoSkyline-Plus. Trace plot of β.

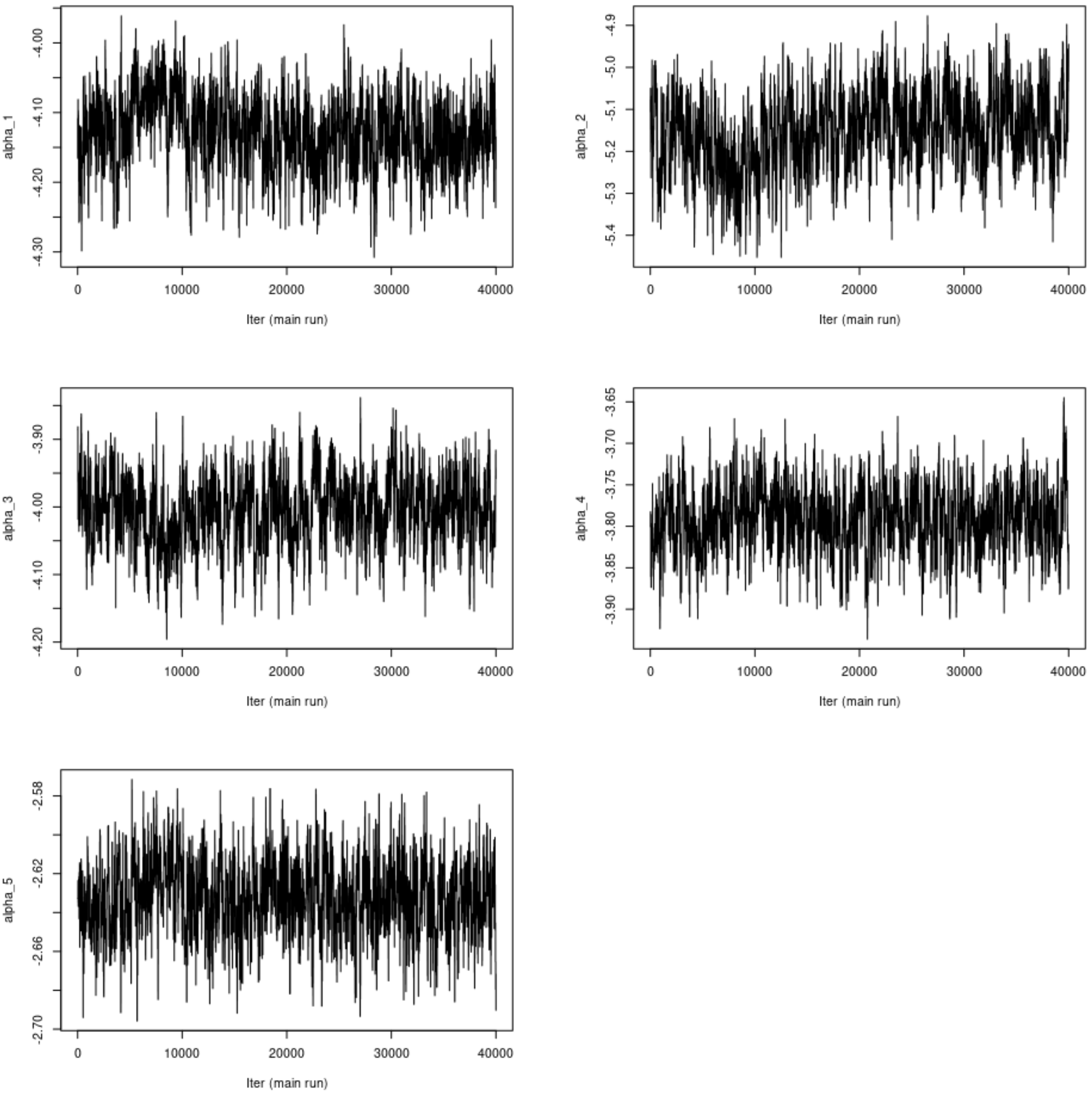

Figure S51: GGPA 2.0 analysis of psychiatric disorders using annotations of GenoSkyline-Plus. Trace plot of α.

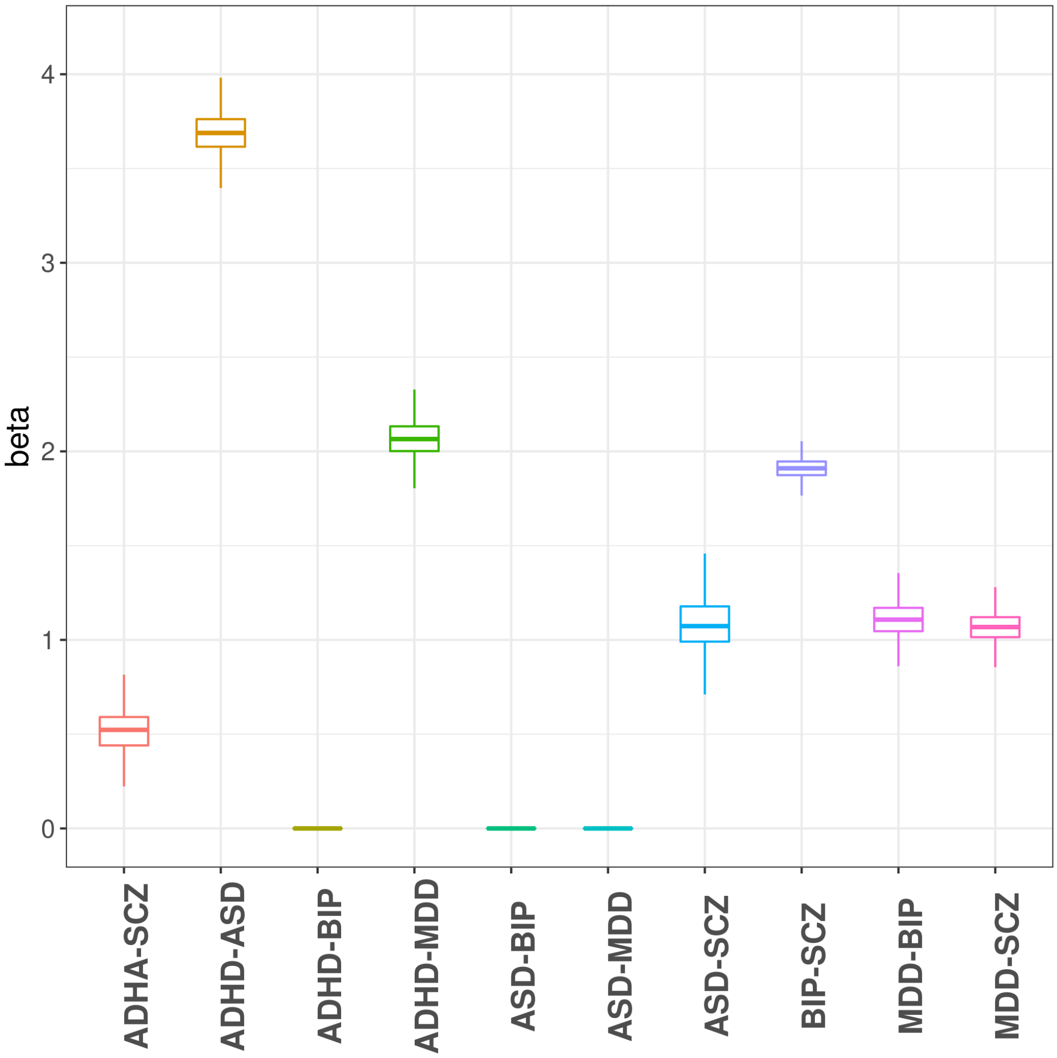

Figure S52: GGPA 2.0 analysis of psychiatric disorders using annotations of GenoSkyline-Plus. Coefficient estimates of β suggest a strong pleiotropy between ADHD and ASD.

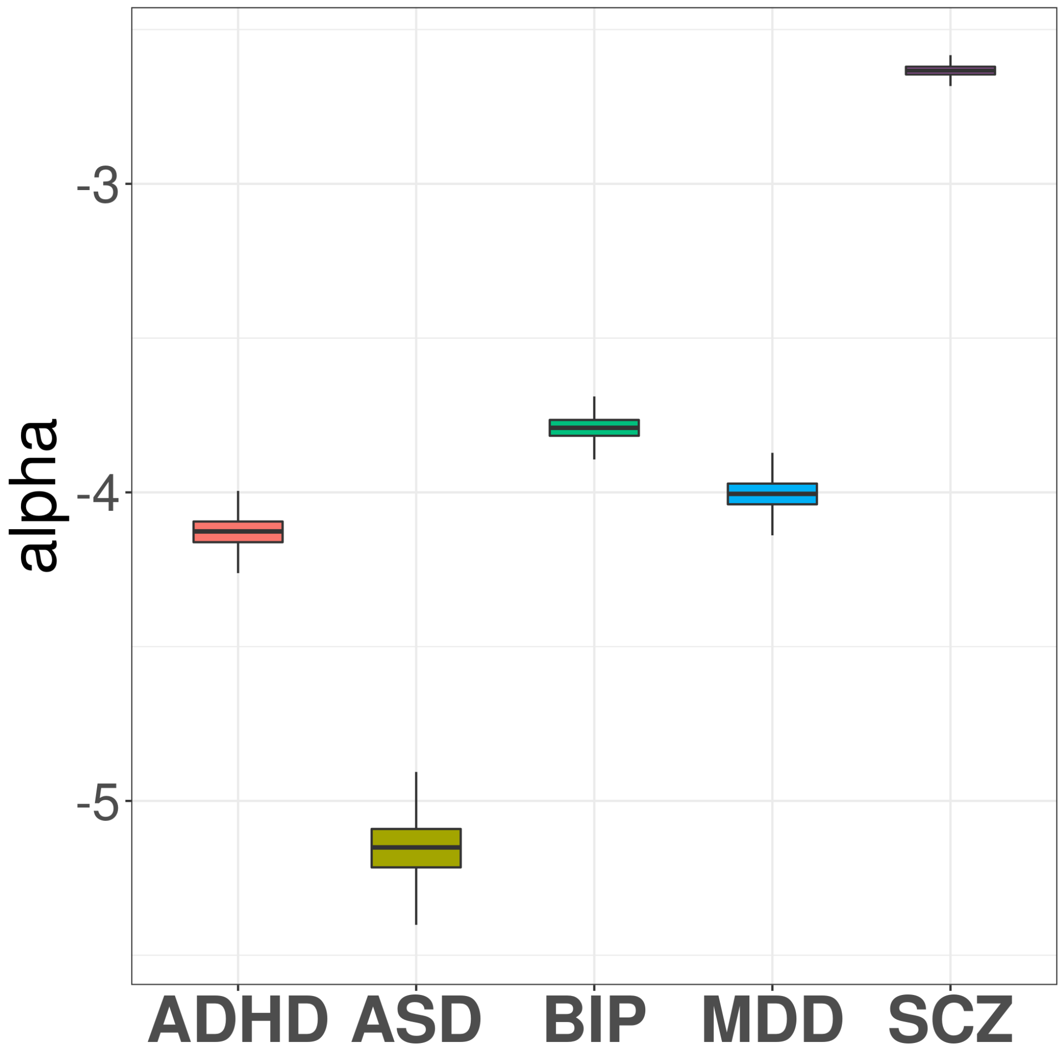

Figure S53: GGPA 2.0 analysis of psychiatric disorders using annotations of GenoSkyline-Plus. Coefficient estimates of α suggest a stronger genetic basis of SCZ compared with other psychiatric disorders.

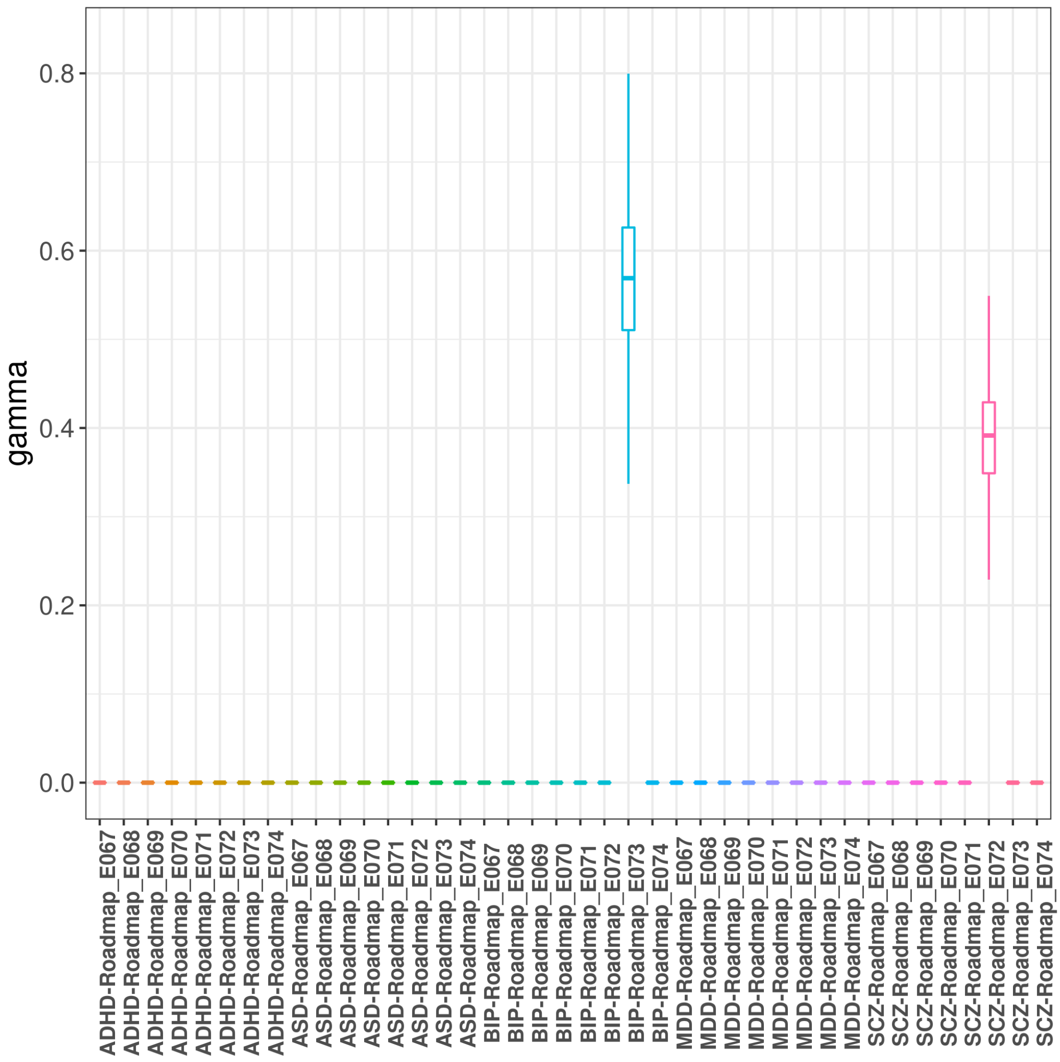

Figure S54: GGPA 2.0 analysis of psychiatric disorders using annotations of GenoSkyline-Plus. Coefficient estimates of γ show that dorsolateral prefrontal cortex is associated with BIP and inferior temporal lobe is associated with SCZ. Roadmap E067: Angular gyrus; Roadmap E068: Anterior caudate; Roadmap E069: Cingulate gyrus; Roadmap E070: Germinal matrix; Roadmap E071: Hippocampus middle; Roadmap E072: Inferior temporal lobe; Roadmap E073: Dorsolateral prefrontal cortex; Roadmap E074: Substantia nigra.

	ADHD	ASD	MDD	BIP	SCZ
ADHD	320	68	48	6	80
ASD	68	196	19	0	55
MDD	48	19	327	7	192
BIP	6	0	7	481	242
SCZ	80	55	192	242	3471

Table S12. GGPA 2.0 analysis of psychiatric disorders using annotations of GenoSkyline-Plus: Numbers of SNPs identified to be associated with each pair of phenotypes with the global FDR at nominal level of 5%. Diagonal elements show the number of SNPs inferred to be associated with each phenotype when the global FDR is controlled at the same level.

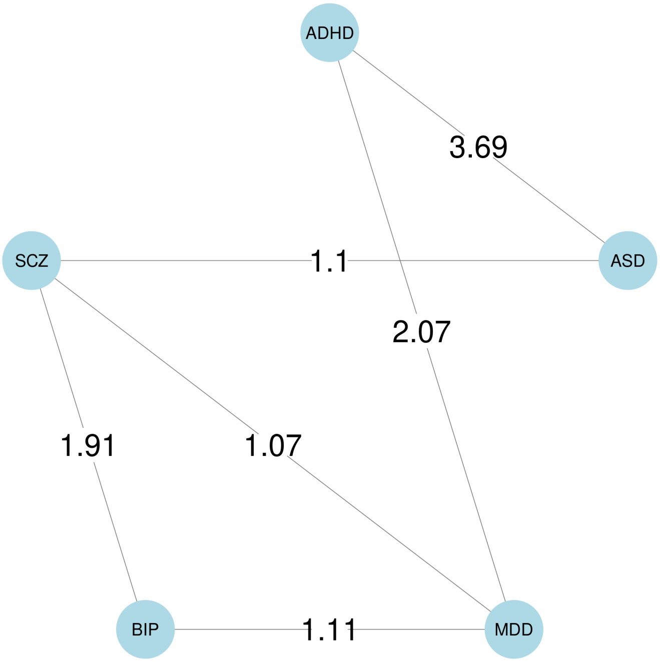

Figure S55: GGPA 2.0 analysis of psychiatric disorders using annotations of GenoSkyline-Plus. Estimated phenotype graph of psychiatric disorders. Values on the edges show β coefficient estimates.

3.2.3 Analysis without Using Functional Annotation

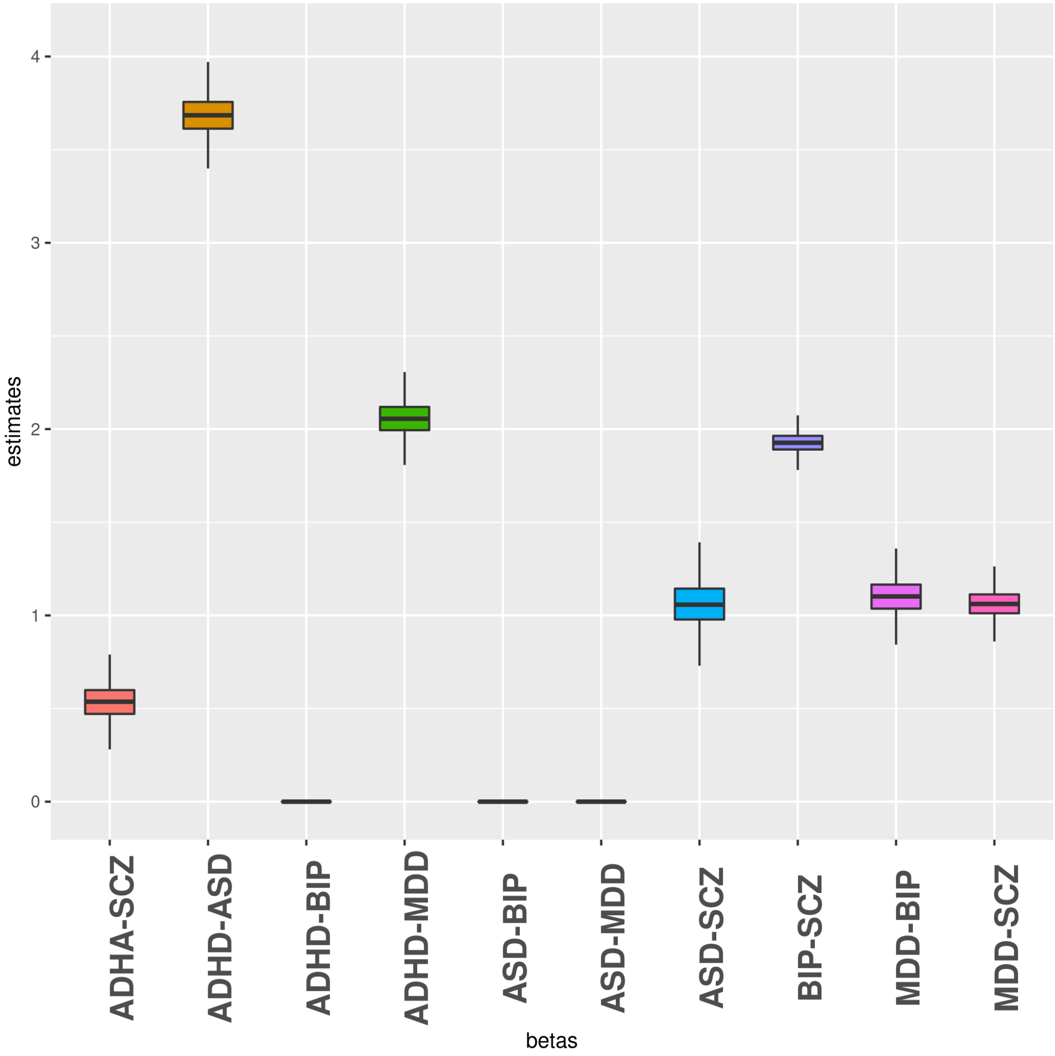

Figure S56: GGPA 2.0 analysis of psychiatric disorders without using functional annotation. Coefficient estimates of β suggest a strong pleiotropy between ADHD and ASD.

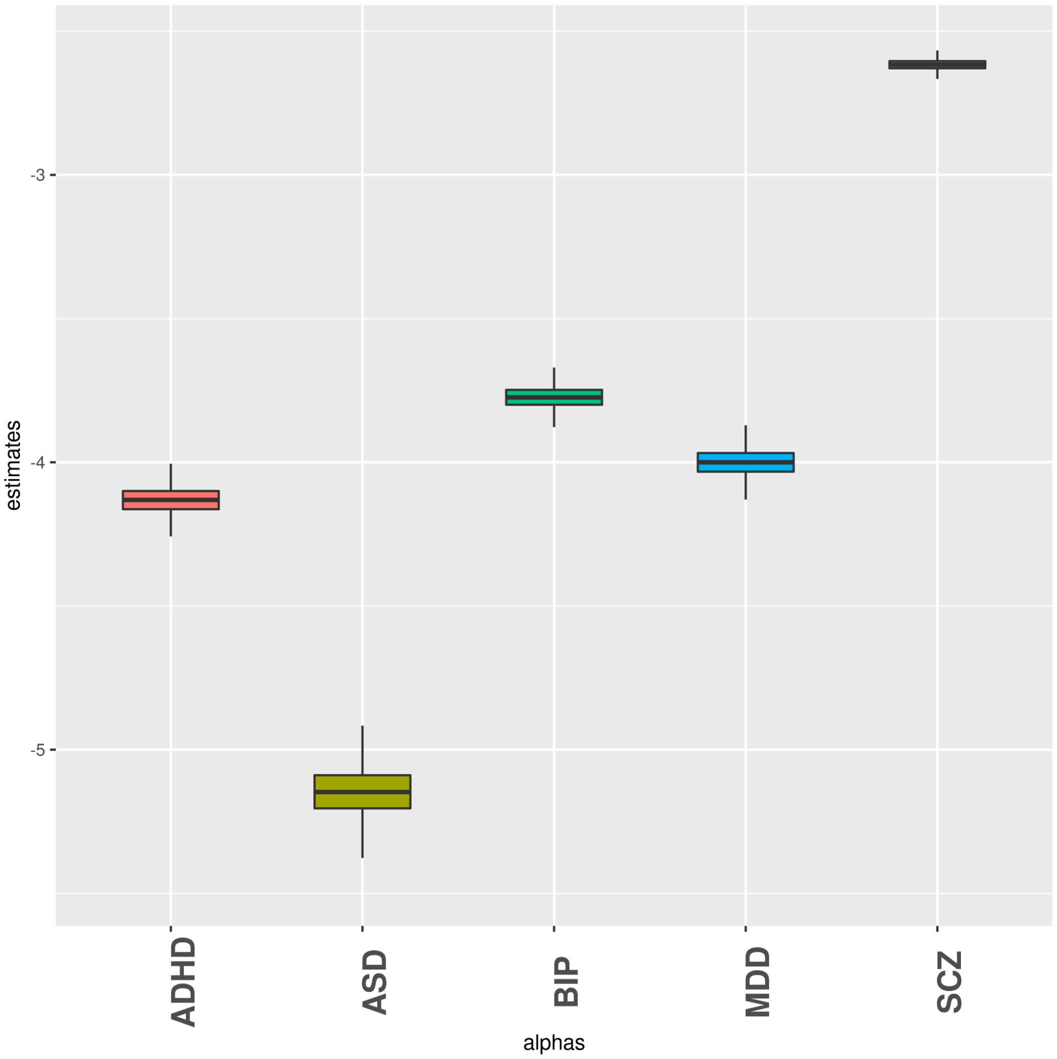

Figure S57: GGPA 2.0 analysis of psychiatric disorders without using functional annotation. Coefficient estimates of α suggest a stronger genetic basis of SCZ compared with other psychiatric disorders.

	ADHD	ASD	MDD	BIP	SCZ
ADHD	322	68	48	6	82
ASD	68	195	19	0	51
MDD	48	19	327	6	192
BIP	6	0	6	476	240
SCZ	82	51	192	240	3461

Table S13. GGPA 2.0 analysis of psychiatric disorders without using functional annotation: Numbers of SNPs identified to be associated with each pair of phenotypes with the global FDR at nominal level of 5%. Diagonal elements show the number of SNPs inferred to be associated with each phenotype when the global FDR is controlled at the same level.

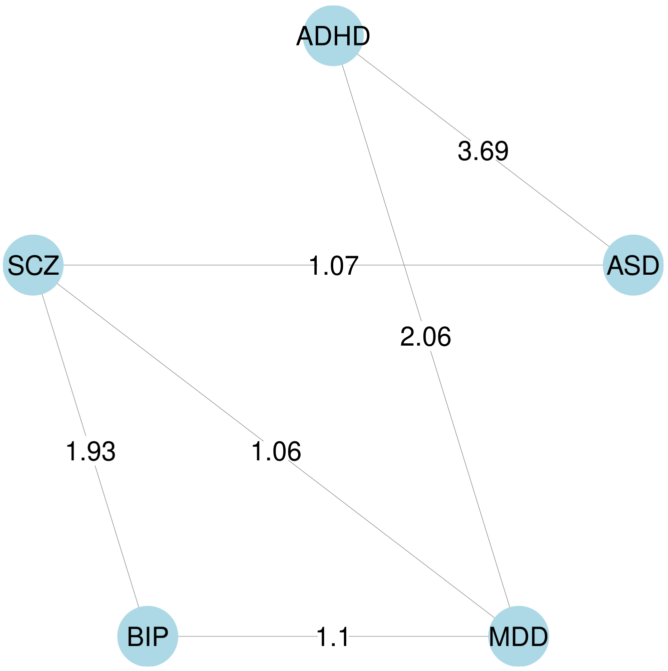

Figure S58: GGPA 2.0 analysis of psychiatric disorders without using functional annotation. Estimated phenotype graph of psychiatric disorders. Values on the edges show β coefficient estimates.

3.2.4 Analysis without Using Prior Disease Graph

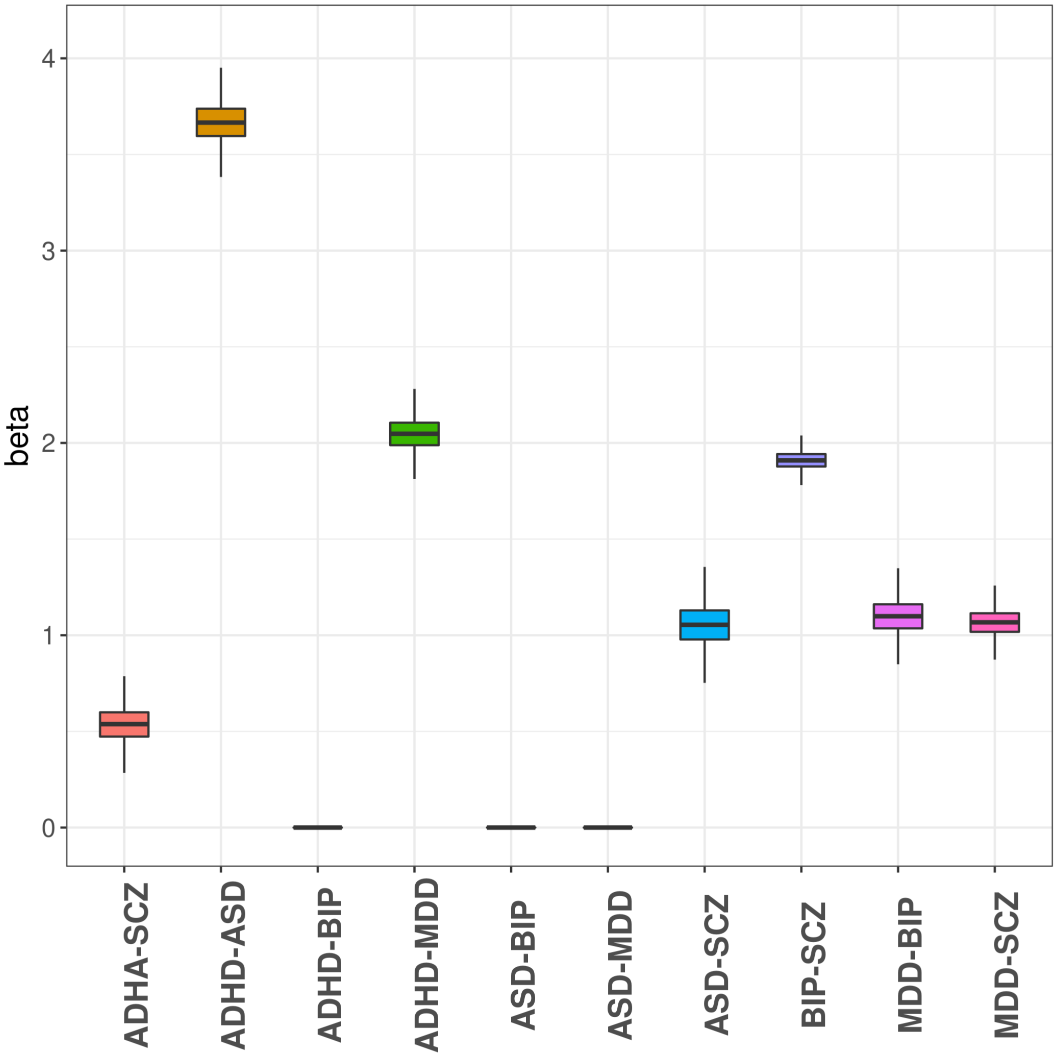

Figure S59: GGPA 2.0 analysis of psychiatric disorders using annotations of GenoSkyline-Plus, but without using the prior disease graph. Coefficient estimates of β suggest a strong pleiotropy between ADHD and ASD.

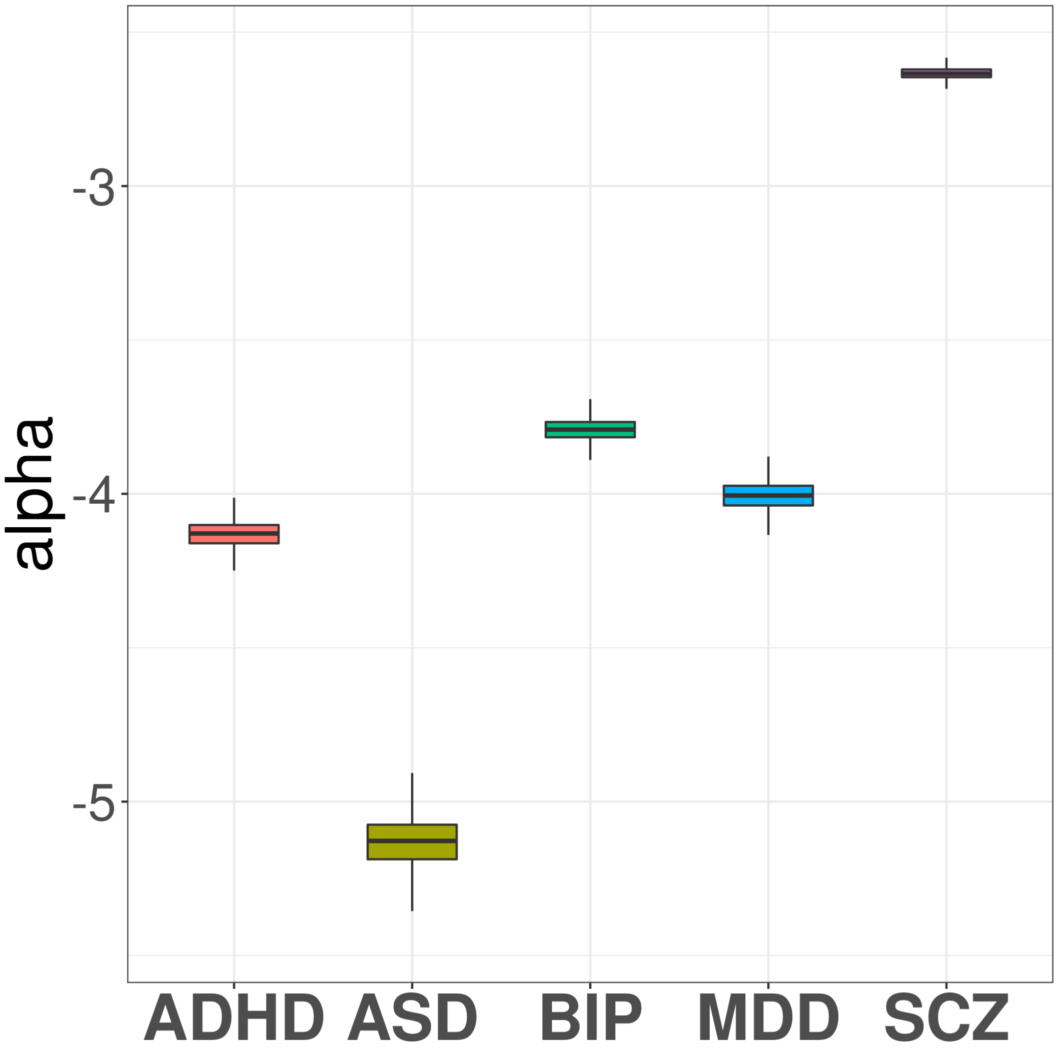

Figure S60: GGPA 2.0 analysis of psychiatric disorders using annotations of GenoSkyline-Plus, but without using the prior disease graph. Coefficient estimates of α suggest a stronger genetic basis of SCZ compared with other psychiatric disorders.

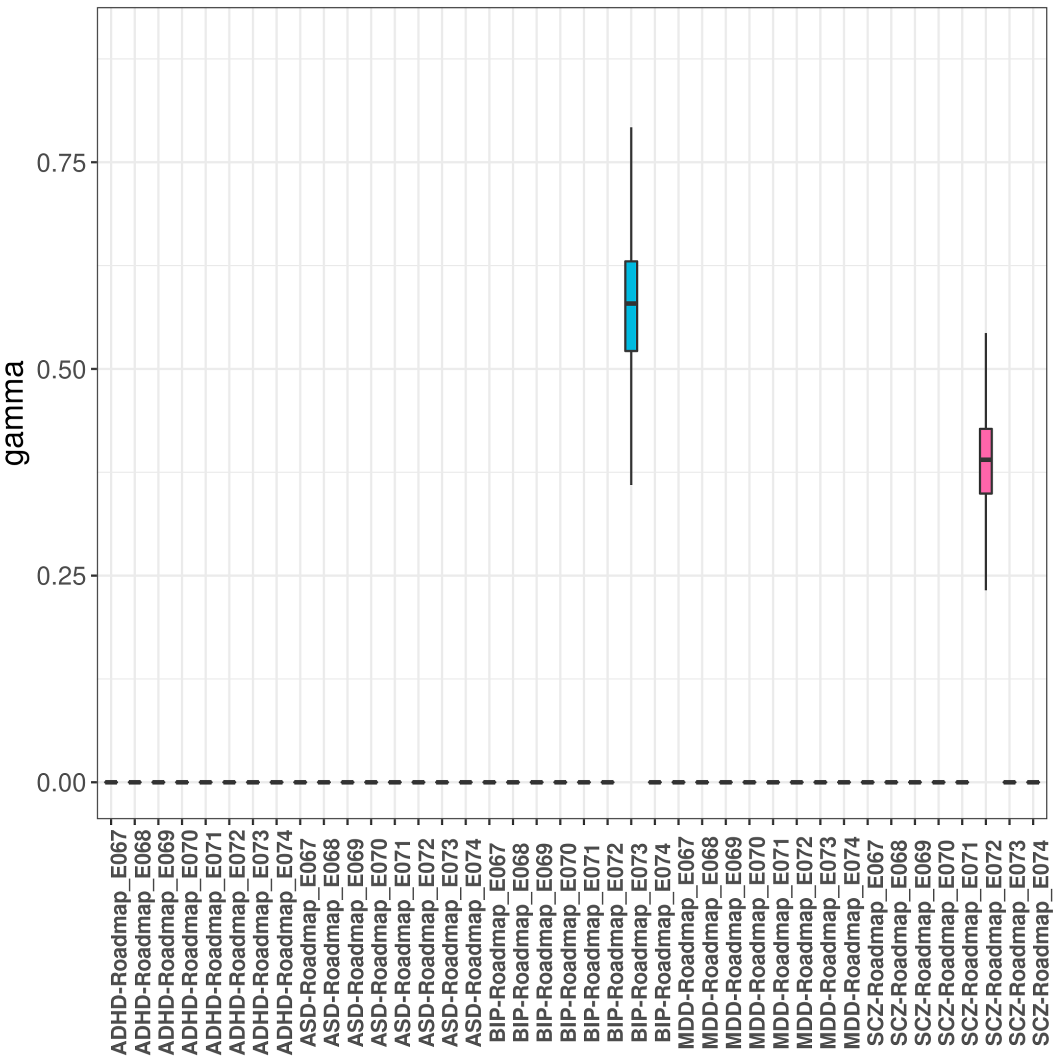

Figure S61: GGPA 2.0 analysis of psychiatric disorders using annotations of GenoSkyline-Plus, but without using the prior disease graph. Coefficient estimates of γ show that dorsolateral prefrontal cortex is associated with BIP and inferior temporal lobe is associated with SCZ. Roadmap E067: Angular gyrus; Roadmap E068: Anterior caudate; Roadmap E069: Cingulate gyrus; Roadmap E070: Germinal matrix; Roadmap E071: Hippocampus middle; Roadmap E072: Inferior temporal lobe; Roadmap E073: Dorsolateral prefrontal cortex; Roadmap E074: Substantia nigra.

	ADHD	ASD	MDD	BIP	SCZ
ADHD	323	66	49	6	83
ASD	66	195	19	0	55
MDD	49	19	328	7	193
BIP	6	0	7	481	243
SCZ	83	55	193	243	3475

Table S14. GGPA 2.0 analysis of psychiatric disorders using annotations of GenoSkyline-Plus, but without using the prior disease graph: Numbers of SNPs identified to be associated with each pair of phenotypes with the global FDR at nominal level of 5%. Diagonal elements show the number of SNPs inferred to be associated with each phenotype when the global FDR is controlled at the same level.

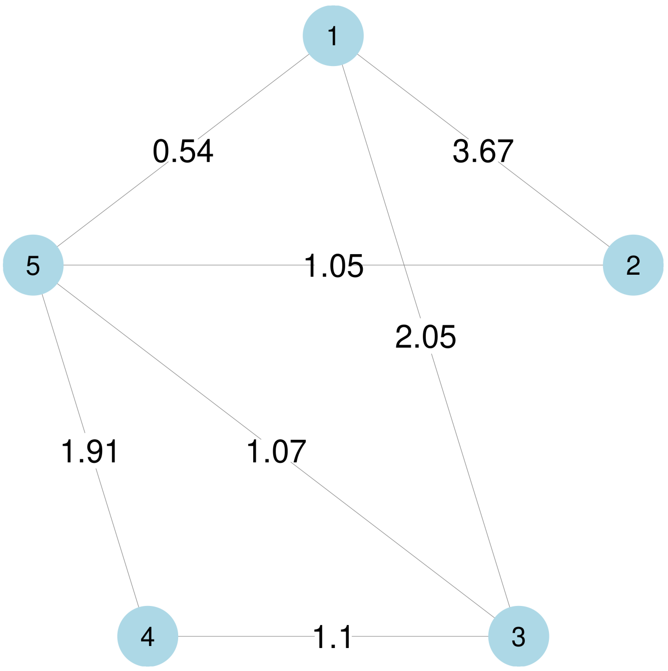

Figure S62: GGPA 2.0 analysis of psychiatric disorders using annotations of GenoSkyline-Plus, but without using the prior disease graph. Estimated phenotype graph of psychiatric disorders. Values on the edges show β coefficient estimates.

3.3 Computation Time of GGPA 2.0

(in minutes)	No annotations	Genoskyline	Genoskyline-Plus
Autoimmune diseases	1,373	2,633	7,033
Psychiatric diseases	1,334	2,816	2,857

Table S15. Computation time (in minutes) of running GGPA 2.0.

4 INVESTIGATION OF THE IMPACTS OF THE USE OF IRRELEVANT/INCORRECT FUNCTIONAL ANNOTATIONS AND MINOR ALLELE FREQUENCY ON THE PERFORMANCE OF GGPA 2.0

4.1 Autoimmune Disease GWAS Data Analysis with Shuffled Functional Annotations

4.1.1 Integration with Genoskyline

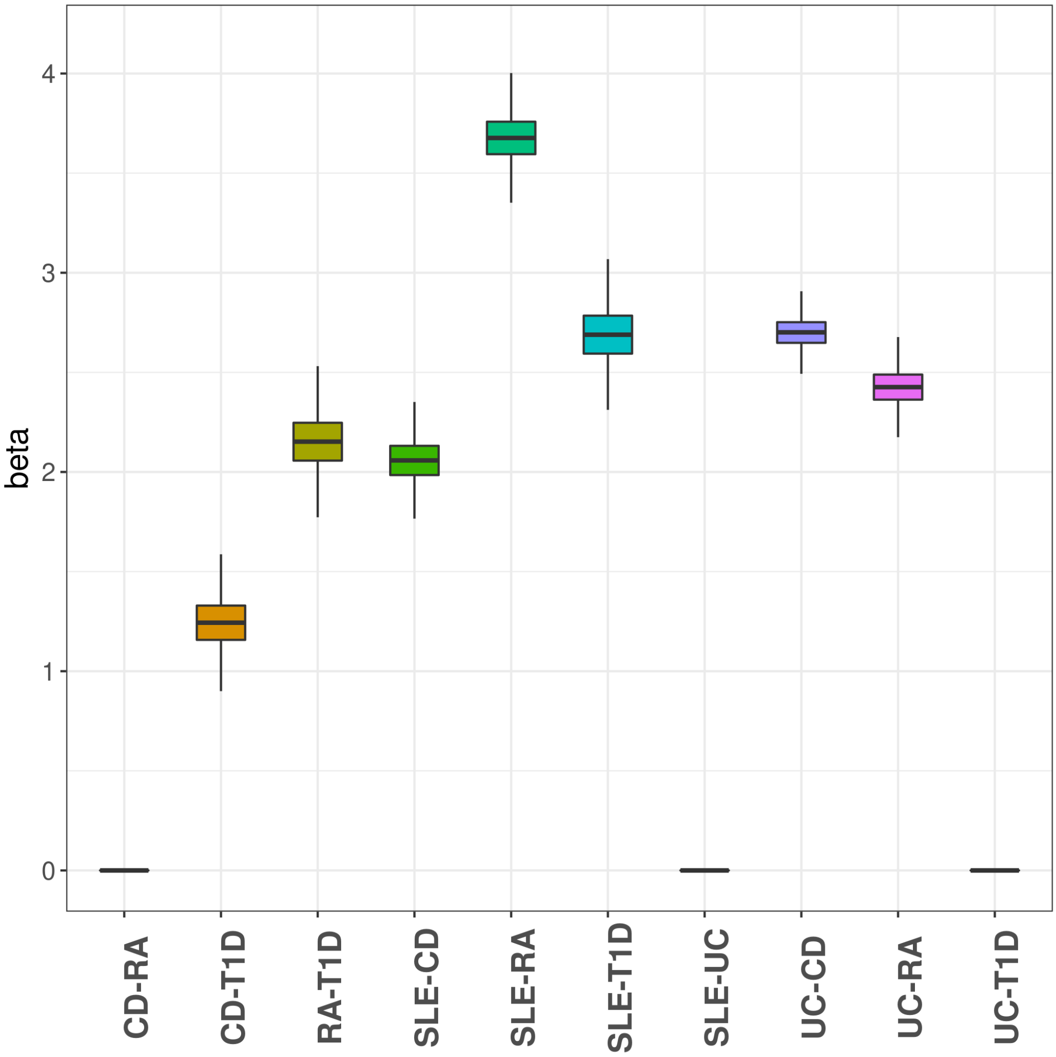

Figure S63: GGPA 2.0 analysis of autoimmune diseases using shuffled annotations of GenoSkyline: β estimates.

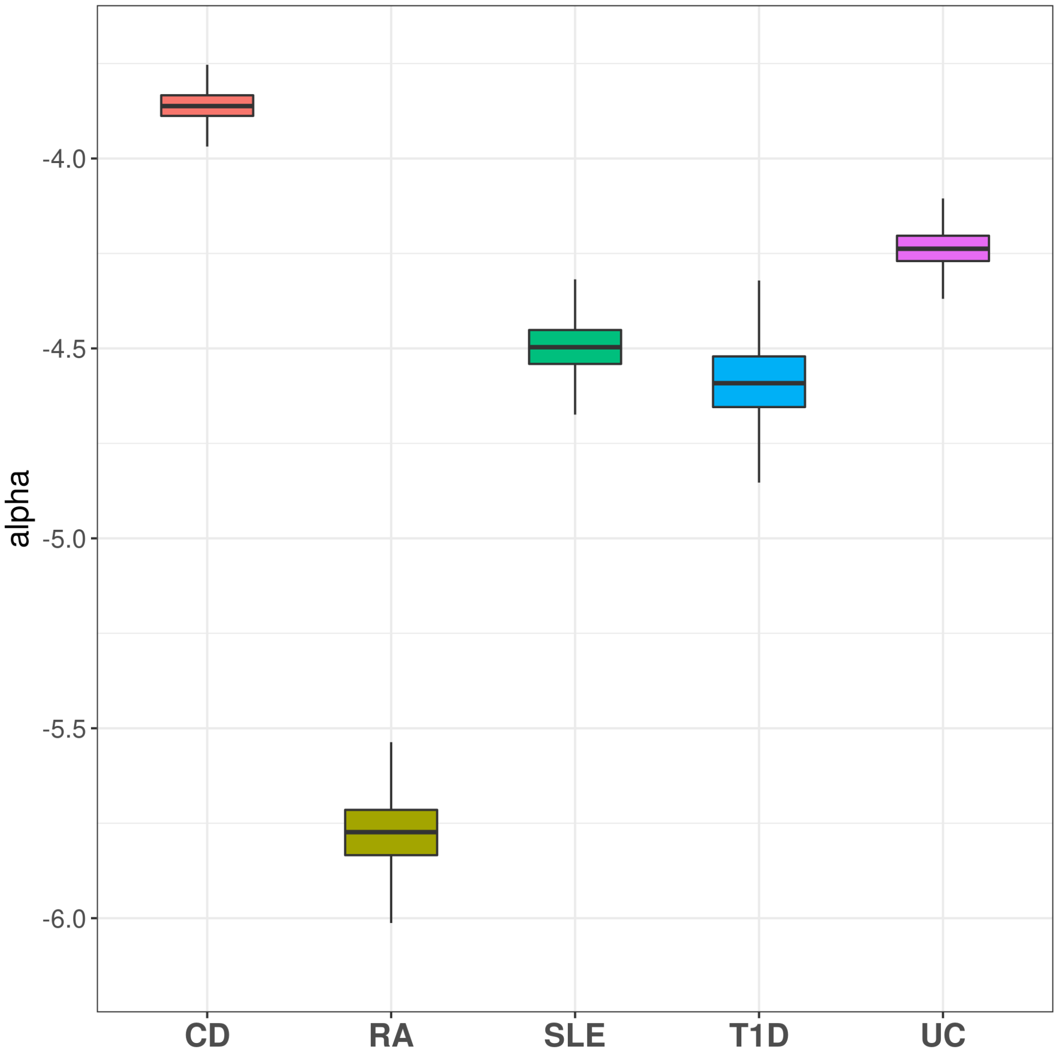

Figure S64: GGPA 2.0 analysis of autoimmune diseases using shuffled annotations of GenoSkyline: α estimates.

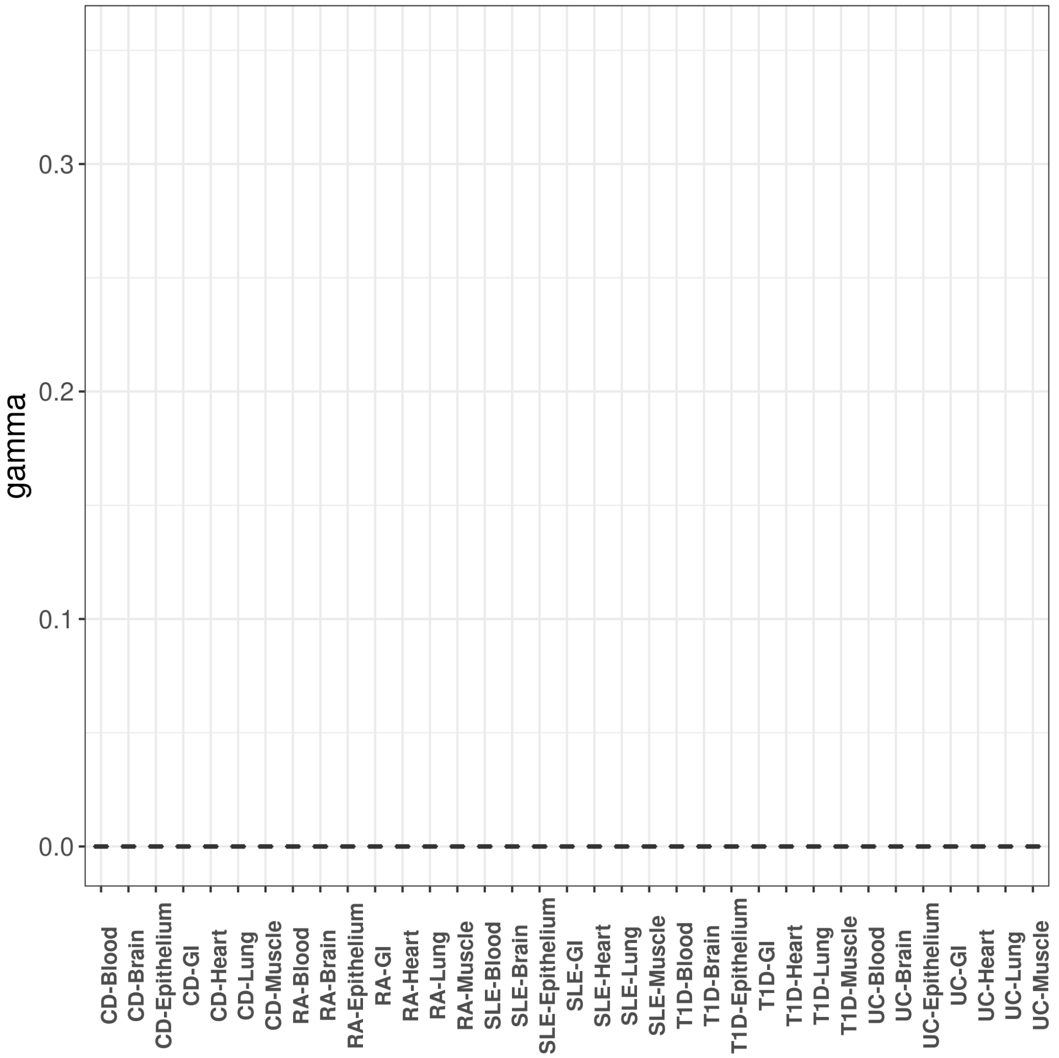

Figure S65: GGPA 2.0 analysis of autoimmune diseases using shuffled annotations of GenoSkyline: γ estimates. No significant annotations exist.

	SLE	UC	CD	RA	T1D
SLE	0.999	1	1.000	0.999	0.999
UC	1.000	1	1.000	1.000	1.000
CD	1.000	1	1.001	1.000	1.000
RA	0.999	1	1.000	1.000	1.000
T1D	0.999	1	1.000	1.000	1.000

Table S16. GGPA 2.0 analysis of autoimmune diseases using shuffled annotations of GenoSkyline. The values are the number of SNPs identified with the shuffled annotations, divided by the number of SNPs identified without the annotations.

4.1.2 Integration with Genoskyline-Plus

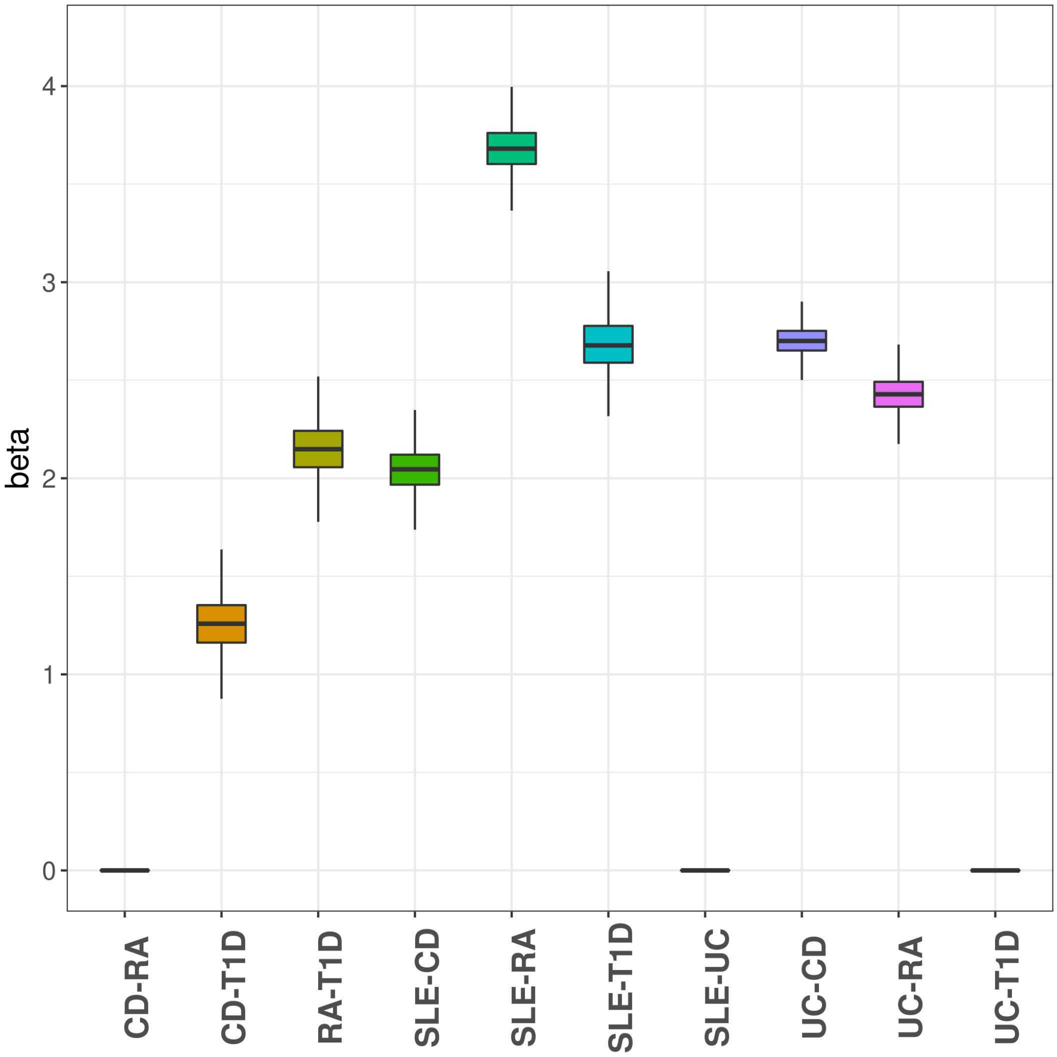

Figure S66: GGPA 2.0 analysis of autoimmune diseases using shuffled annotations of GenoSkyline-Plus: β estimates.

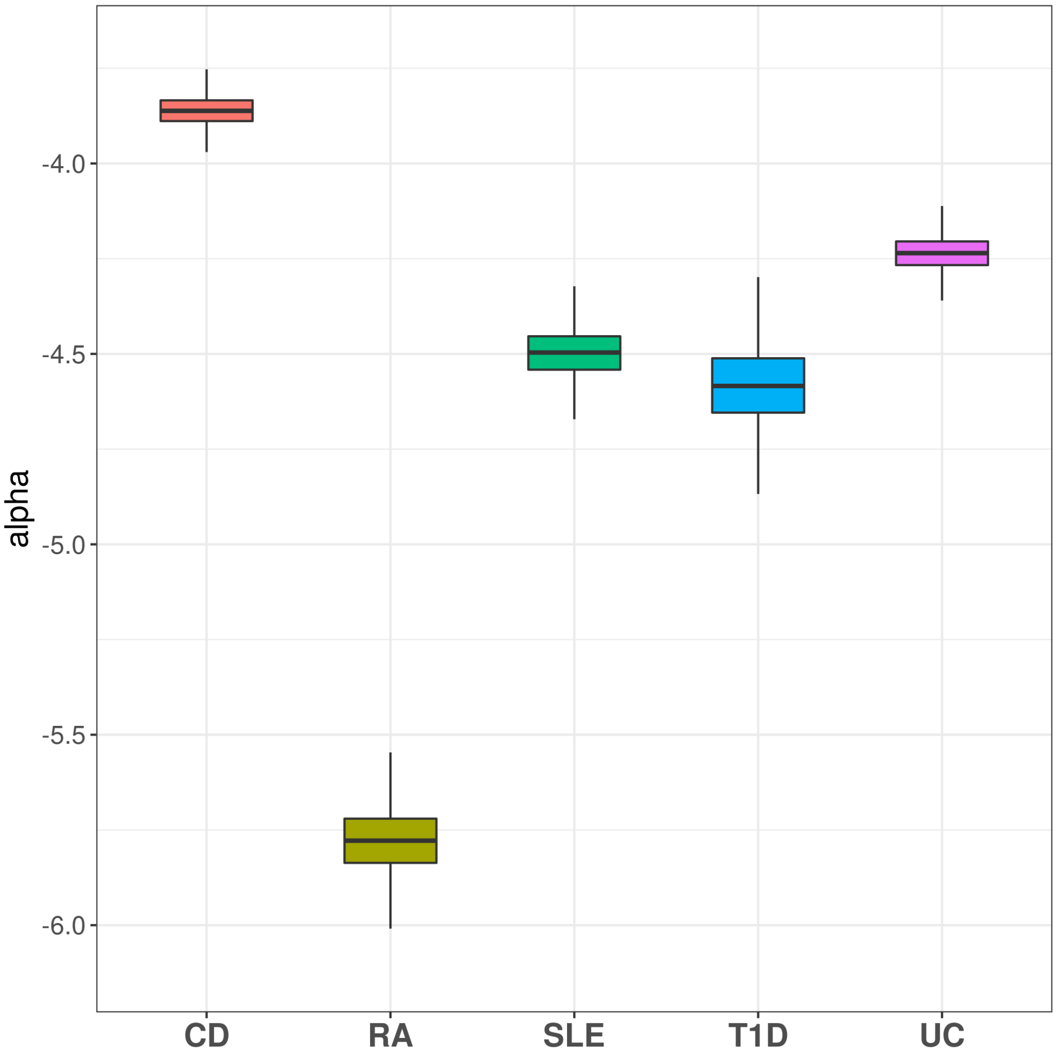

Figure S67: GGPA 2.0 analysis of autoimmune diseases using shuffled annotations of GenoSkyline-Plus: α estimates.

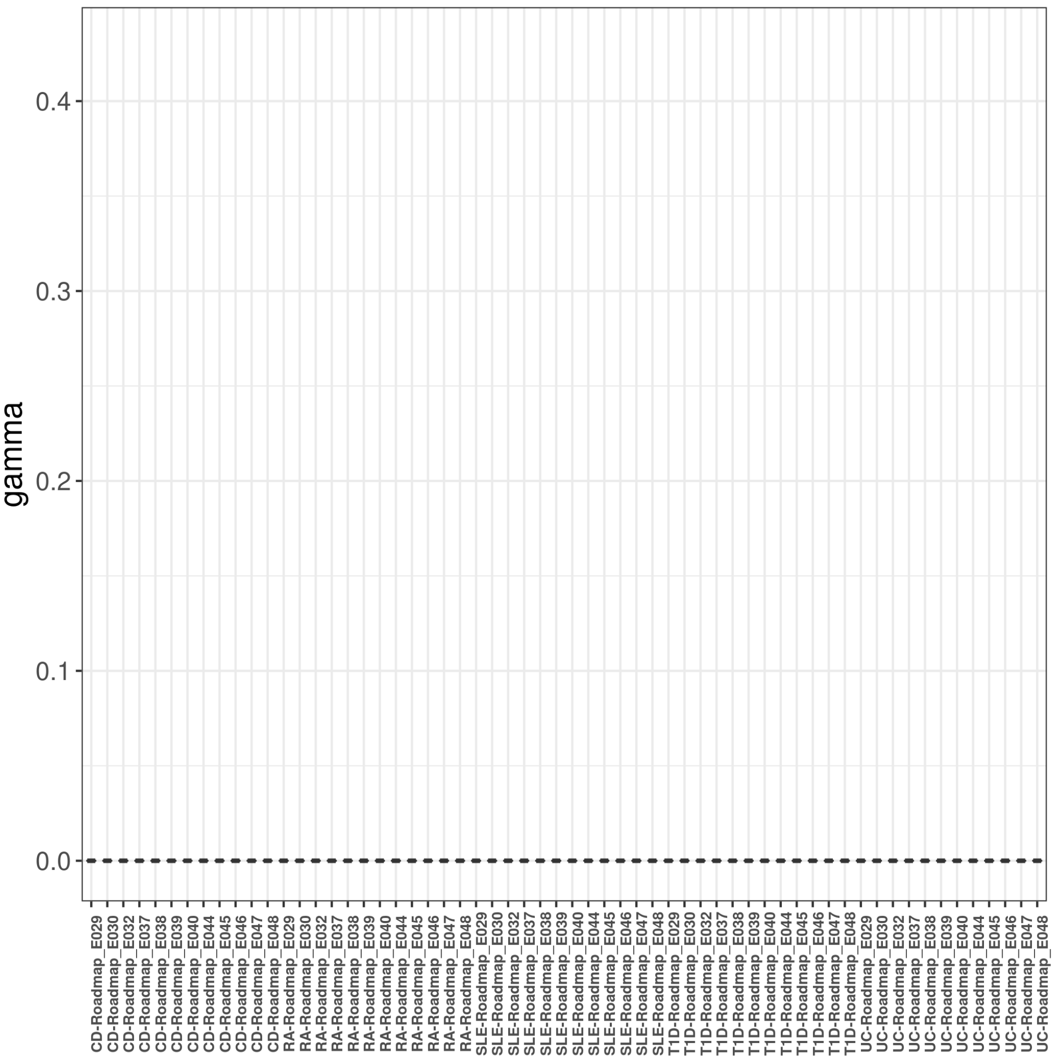

Figure S68: GGPA 2.0 analysis of autoimmune diseases using shuffled annotations of GenoSkyline-Plus: γ estimates. No significant annotations exist.

	SLE	UC	CD	RA	T1D
SLE	0.999	1	0.998	0.999	0.999
UC	1.000	1	1.000	1.000	1.000
CD	0.998	1	1.000	0.998	1.002
RA	0.999	1	0.998	1.000	1.001
T1D	0.999	1	1.002	1.001	1.000

Table S17. GGPA 2.0 analysis of autoimmune diseases using shuffled annotations of GenoSkyline-Plus. The values are the number of SNPs identified with the shuffled annotations, divided by the number of SNPs identified without the annotations.

4.2 Minor Allele Frequency as An Annotation

4.2.1 Autoimmune Disease GWAS Data Analysis with GenoSkyline

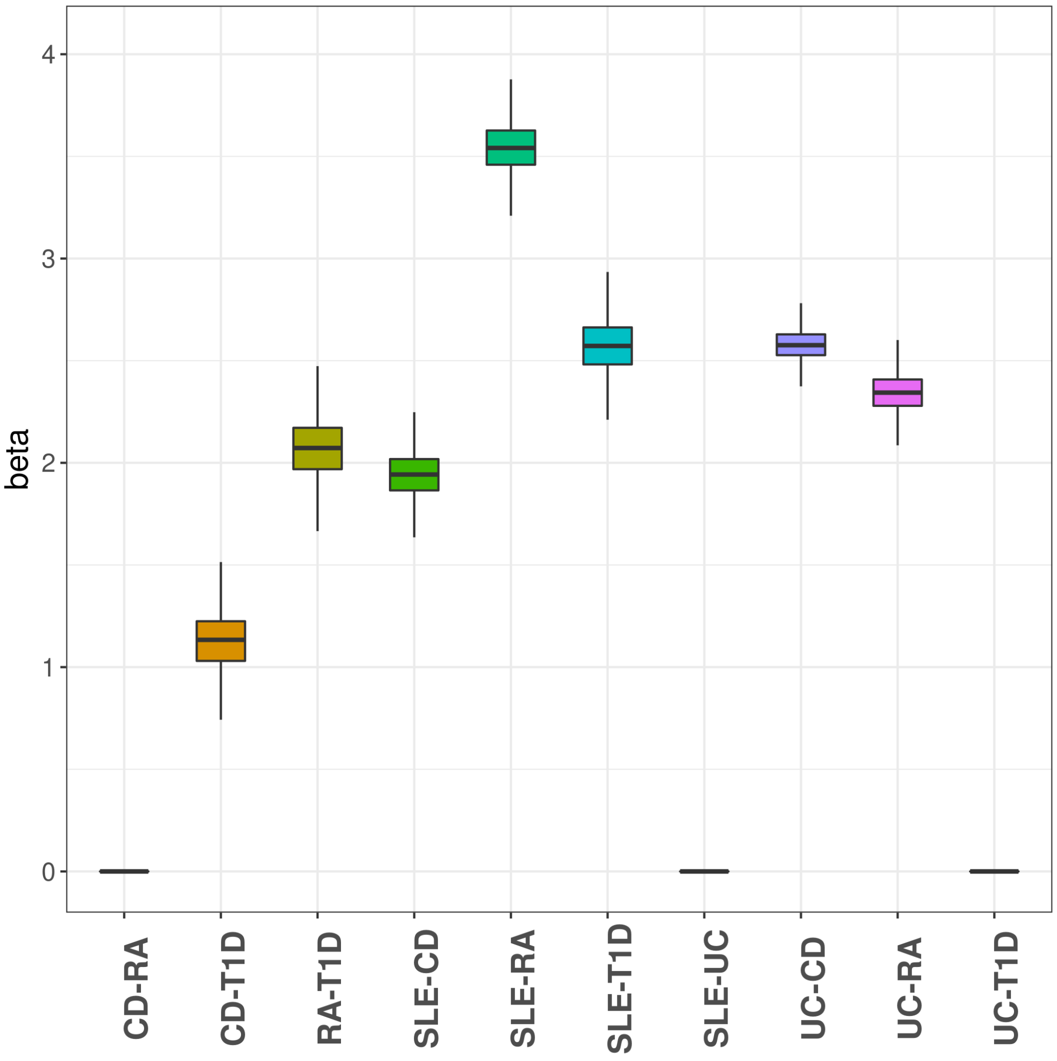

Figure S69: GGPA 2.0 analysis of autoimmune diseases using annotations of GenoSkyline and minor allele frequency (MAF): β estimates. The MAF annotation assigns 1 to SNPs with MAF less than 0.05 and 0 otherwise.

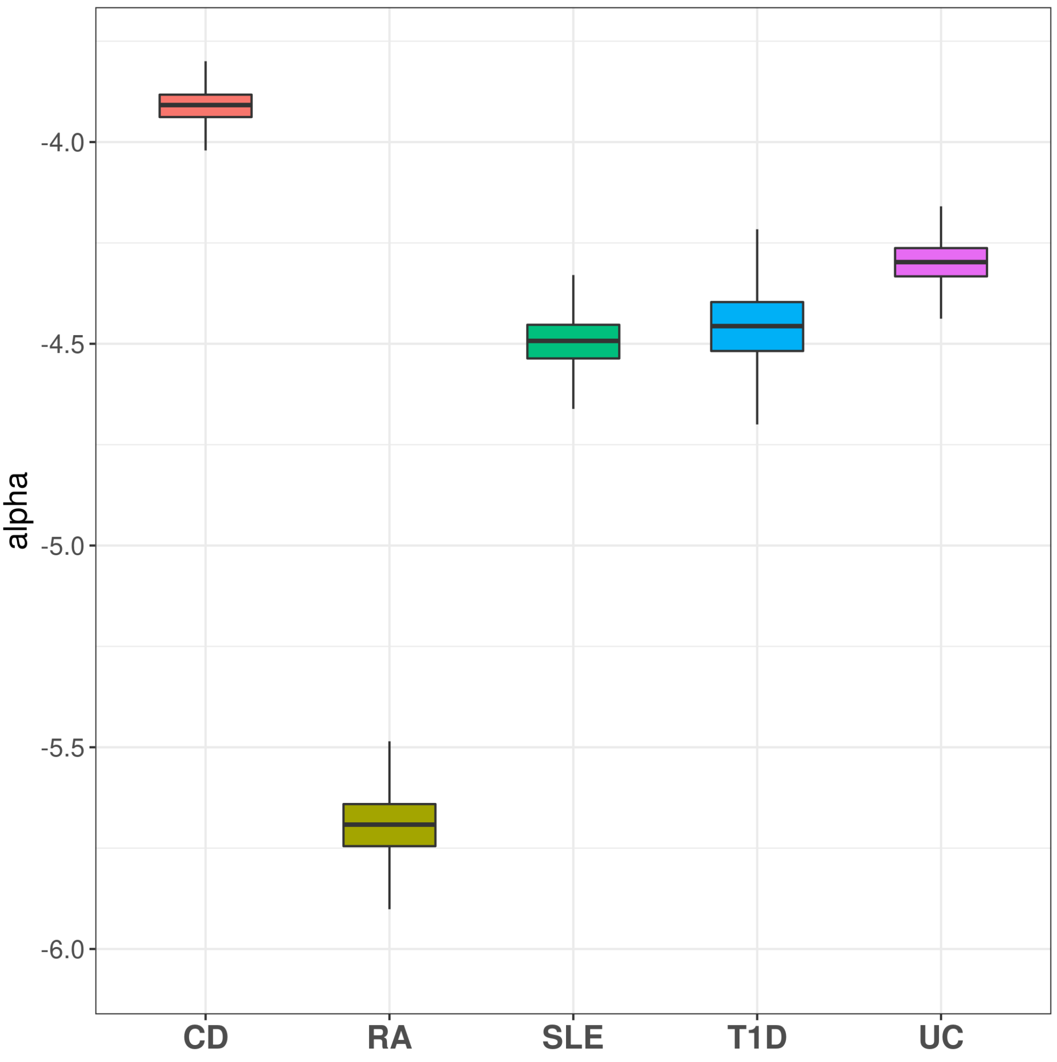

Figure S70: GGPA 2.0 analysis of autoimmune diseases using annotations of GenoSkyline and minor allele frequency (MAF): α estimates. The MAF annotation assigns 1 to SNPs with MAF less than 0.05 and 0 otherwise.

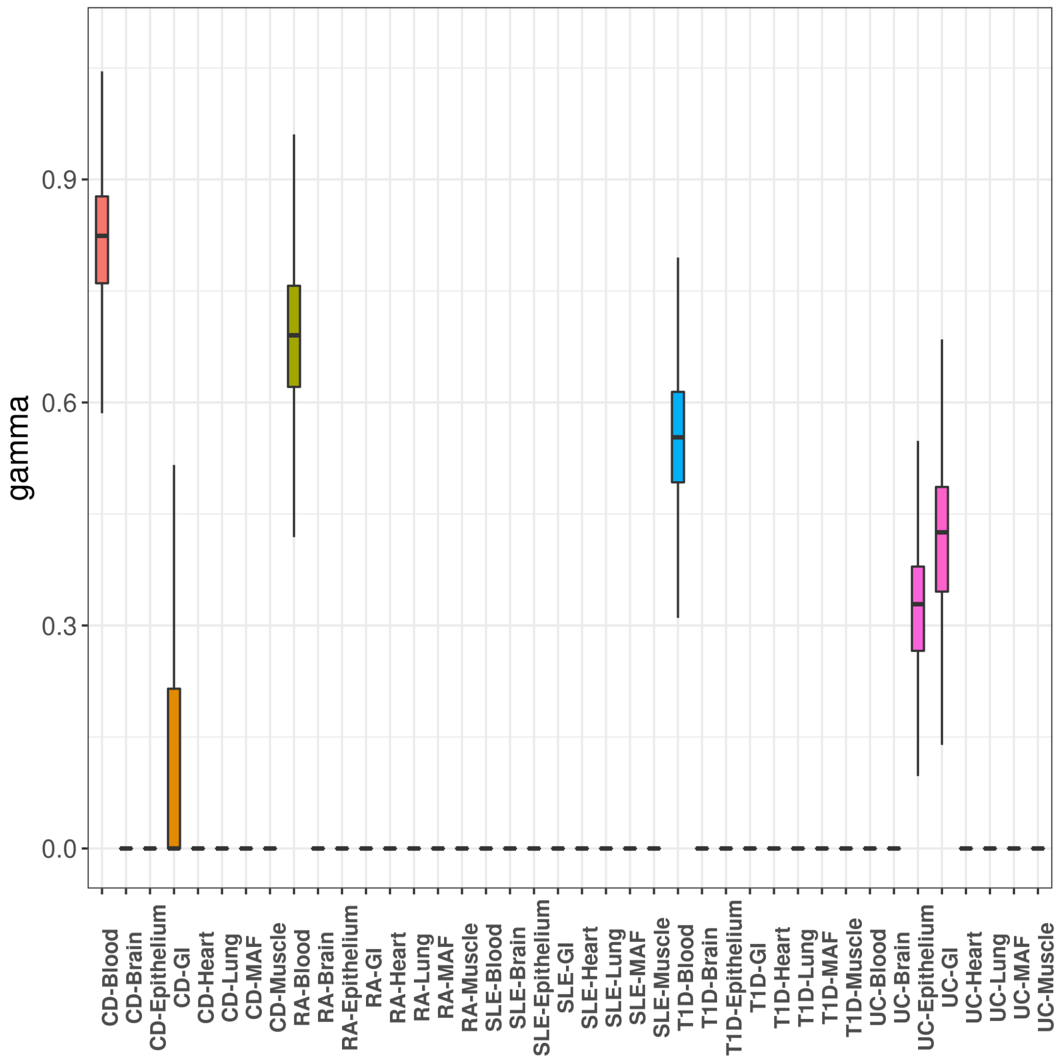

Figure S71: GGPA 2.0 analysis of autoimmune diseases using annotations of GenoSkyline and minor allele frequency (MAF): γ estimates. The MAF annotation assigns 1 to SNPs with MAF less than 0.05 and 0 otherwise.

4.2.2 Psychiatric Disorder GWAS Data Analysis with GenoSkyline

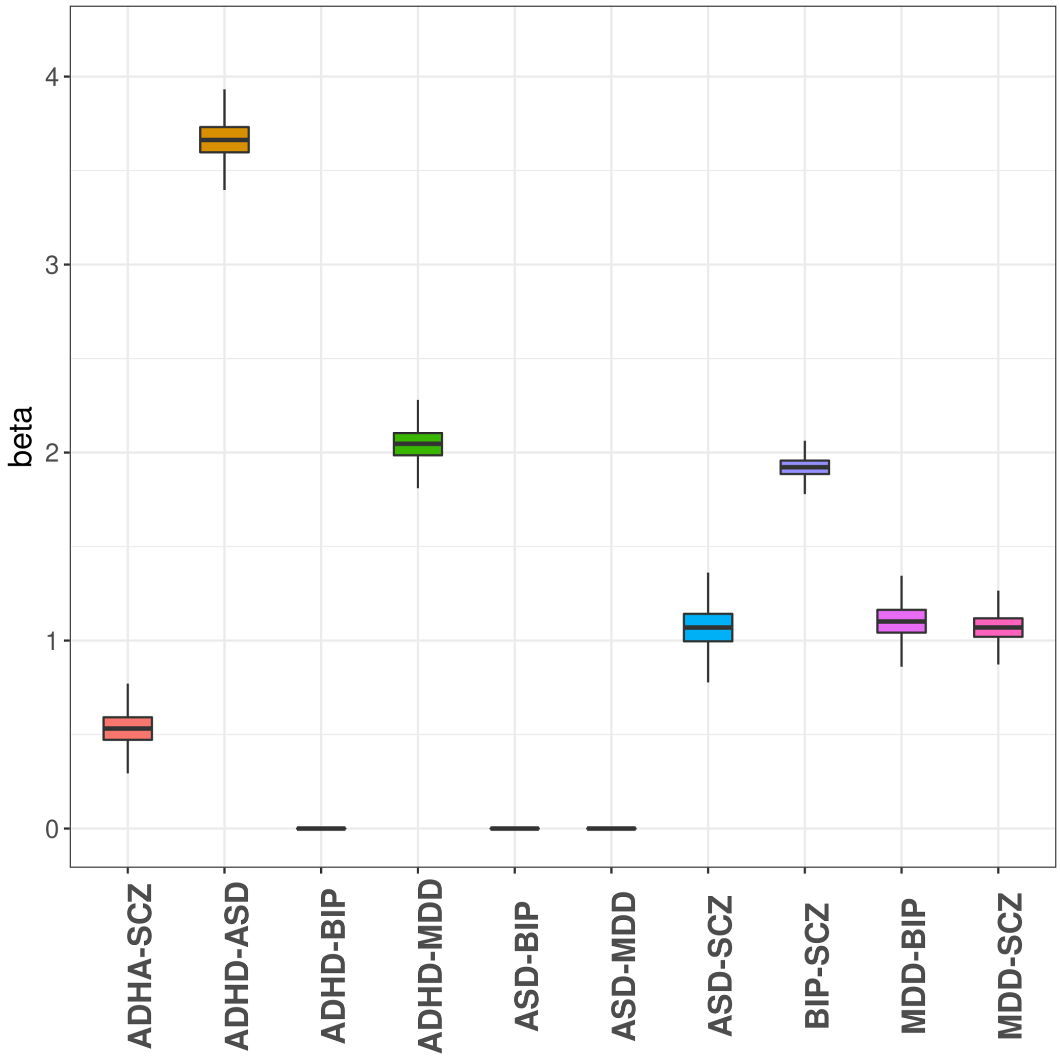

Figure S72: GGPA 2.0 analysis of psychiatric disorders using annotations of GenoSkyline and minor allele frequency (MAF): β estimates. The MAF annotation assigns 1 to SNPs with MAF less than 0.05 and 0 otherwise.

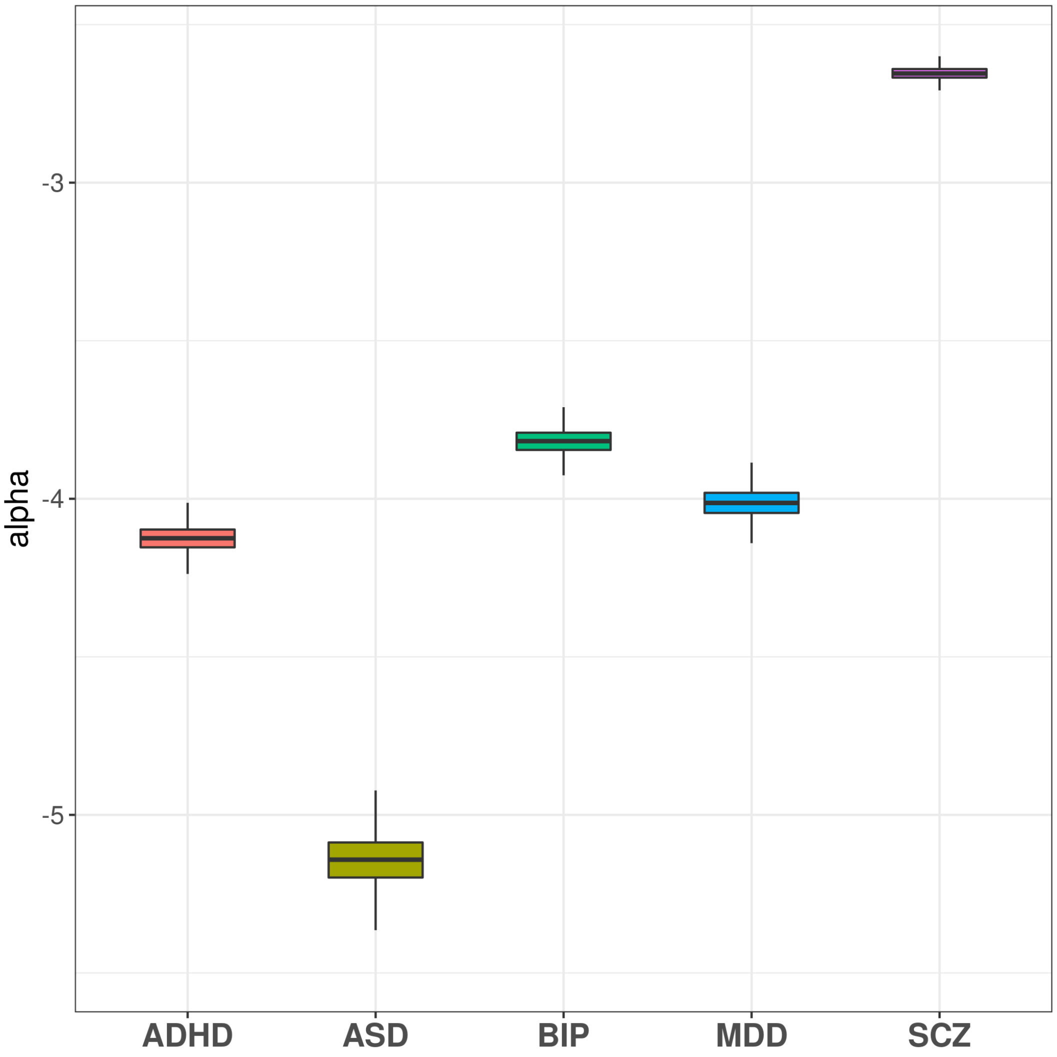

Figure S73: GGPA 2.0 analysis of psychiatric disorders using annotations of GenoSkyline and minor allele frequency (MAF): α estimates. The MAF annotation assigns 1 to SNPs with MAF less than 0.05 and 0 otherwise.

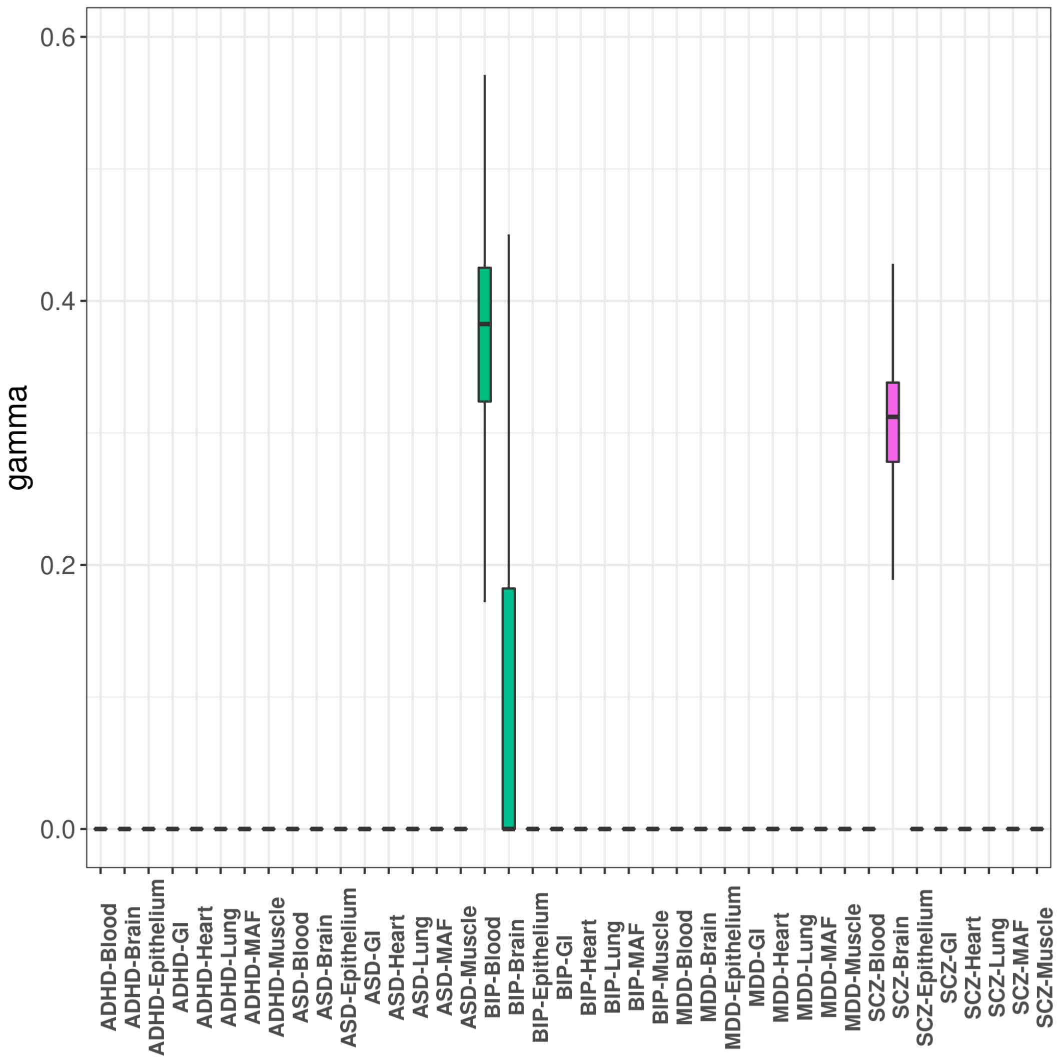

Figure S74: GGPA 2.0 analysis of psychiatric disorders using annotations of GenoSkyline and minor allele frequency (MAF): γ estimates. The MAF annotation assigns 1 to SNPs with MAF less than 0.05 and 0 otherwise.

5 BENCHMARKING

5.1 Comparison of Genetic Correlation Estimation

	SLE	UC	CD	RA	T1D
SLE	1.00	0.69	0.80	0.96	0.94
UC	0.69	1.00	0.83	0.83	0.74
CD	0.80	0.83	1.00	0.79	0.78
RA	0.96	0.83	0.79	1.00	0.94
T1D	0.94	0.74	0.78	0.94	1.00

Table S18. Genetic correlation estimated using LPM for the autoimmune diseases, where GenoSkyline is used as functional annotation data.

	SLE	UC	CD	RA	T1D
SLE	1.00	0.69	0.80	0.96	0.95
UC	0.69	1.00	0.84	0.84	0.75
CD	0.80	0.84	1.00	0.80	0.79
RA	0.96	0.84	0.80	1.00	0.94
T1D	0.95	0.75	0.79	0.94	1.00

Table S19. Genetic correlation estimated using LPM for the autoimmune diseases, where GenoSkylinePlus is used as functional annotation data.

	SLE	UC	CD	RA	T1D
SLE	1.00	0.68	0.67	0.63	0.66
UC	0.68	1.00	0.73	0.66	0.70
CD	0.67	0.73	1.00	0.65	0.69
RA	0.63	0.66	0.65	1.00	0.64
T1D	0.66	0.70	0.69	0.64	1.00

Table S20. Genetic correlation estimated using LDSC for the autoimmune diseases.

	ADHD	ASD	MDD	BIP	SCZ
ADHD	1.00	0.94	0.80	0.35	0.44
ASD	0.94	1.00	0.67	0.36	0.63
MDD	0.80	0.67	1.00	0.48	0.48
BIP	0.35	0.36	0.48	1.00	0.73
SCZ	0.44	0.63	0.48	0.73	1.00

Table S21. Genetic correlation estimated using LPM for the psychiatric diseases, where GenoSkyline is used as functional annotation data.

	ADHD	ASD	MDD	BIP	SCZ
ADHD	1.00	0.94	0.80	0.34	0.43
ASD	0.94	1.00	0.67	0.37	0.63
MDD	0.80	0.67	1.00	0.48	0.47
BIP	0.34	0.37	0.48	1.00	0.73
SCZ	0.43	0.63	0.47	0.73	1.00

Table S22. Genetic correlation estimated using LPM for the psychiatric diseases, where GenoSkylinePlus is used as functional annotation data.

	ADHD	ASD	MDD	BIP	SCZ
ADHD	1.00	0.69	0.66	0.66	0.68
ASD	0.69	1.00	0.65	0.66	0.68
MDD	0.66	0.65	1.00	0.66	0.68
BIP	0.66	0.66	0.66	1.00	0.70
SCZ	0.68	0.68	0.68	0.70	1.00

Table S23. Genetic correlation estimated using LDSC for the psychiatric diseases.

5.2 Comparison of Association Mapping

	RA	UC	CD	T1D	SLE
GGPA2 + GenoSkyline	1294	1103	1918	1358	1671
GGPA2 + GenoSkylinePlus	1268	1100	1898	1353	1680
LPM + GenoSkyline	903	722	1462	940	1103
LPM + GenoSkylinePlus	890	706	1432	931	1100
MTAG	895	680	1527	572	698

Table S24. Association mapping results for the autoimmune diseases using GGPA 2.0, LPM, and MTAG. FDR is controlled at the nominal level of 0.05 for all the methods, where we used the embedded methods for GGPA 2.0 and LPM and the Benjamini-Hochberg procedure for MTAG.

	ADHD	ASD	MDD	BIP	SCZ
GGPA2 + GenoSkyline	356	210	342	561	3961
GGPA2 + GenoSkylinePlus	320	196	327	481	3471
LPM + GenoSkyline	173	40	232	330	3314
LPM + GenoSkylinePlus	168	35	233	330	3319
MTAG	289	247	419	1742	4841

Table S25. Association mapping results for the psychiatric diseases using GGPA 2.0, LPM, and MTAG. FDR is controlled at the nominal level of 0.05 for all the methods, where we used the embedded methods for GGPA 2.0 and LPM and the Benjamini-Hochberg procedure for MTAG.

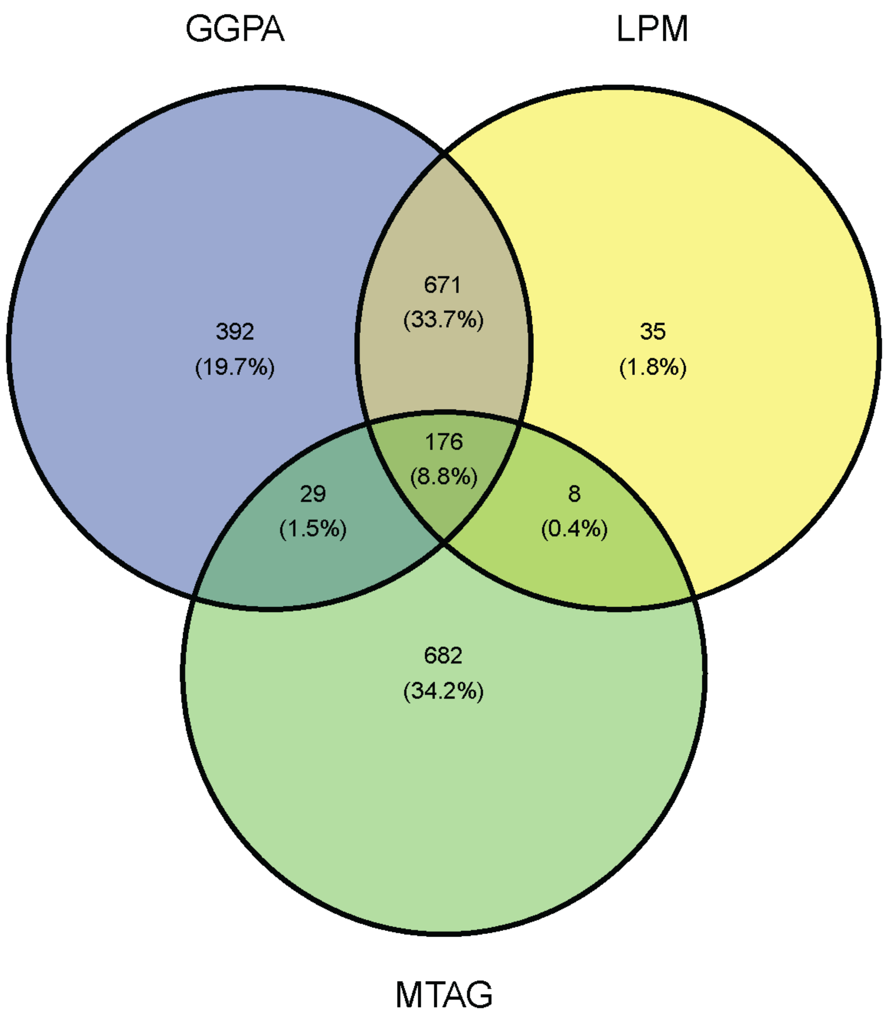

Figure S75: Venn diagram showing the overlap of the risk SNPs identified by GGPA 2.0, LPM, and MTAG at the nominal level of FDR 0.05 for RA.

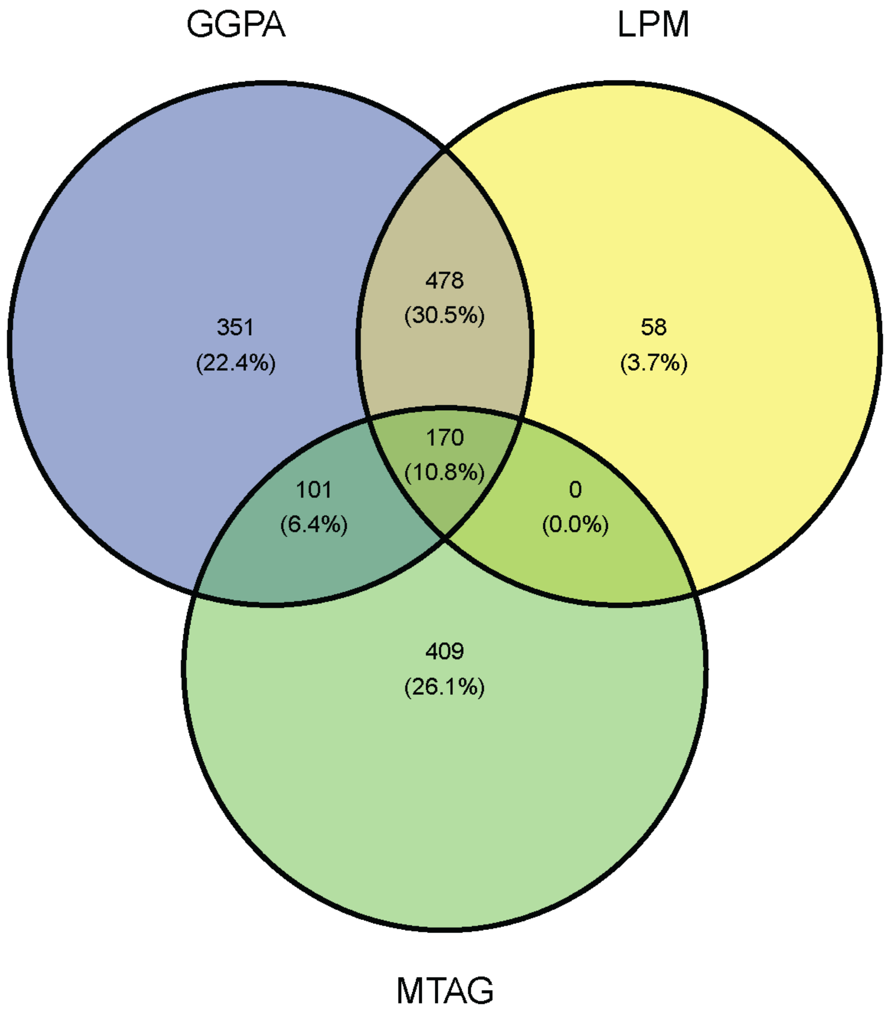

Figure S76: Venn diagram showing the overlap of the risk SNPs identified by GGPA 2.0, LPM, and MTAG at the nominal level of FDR 0.05 for UC.

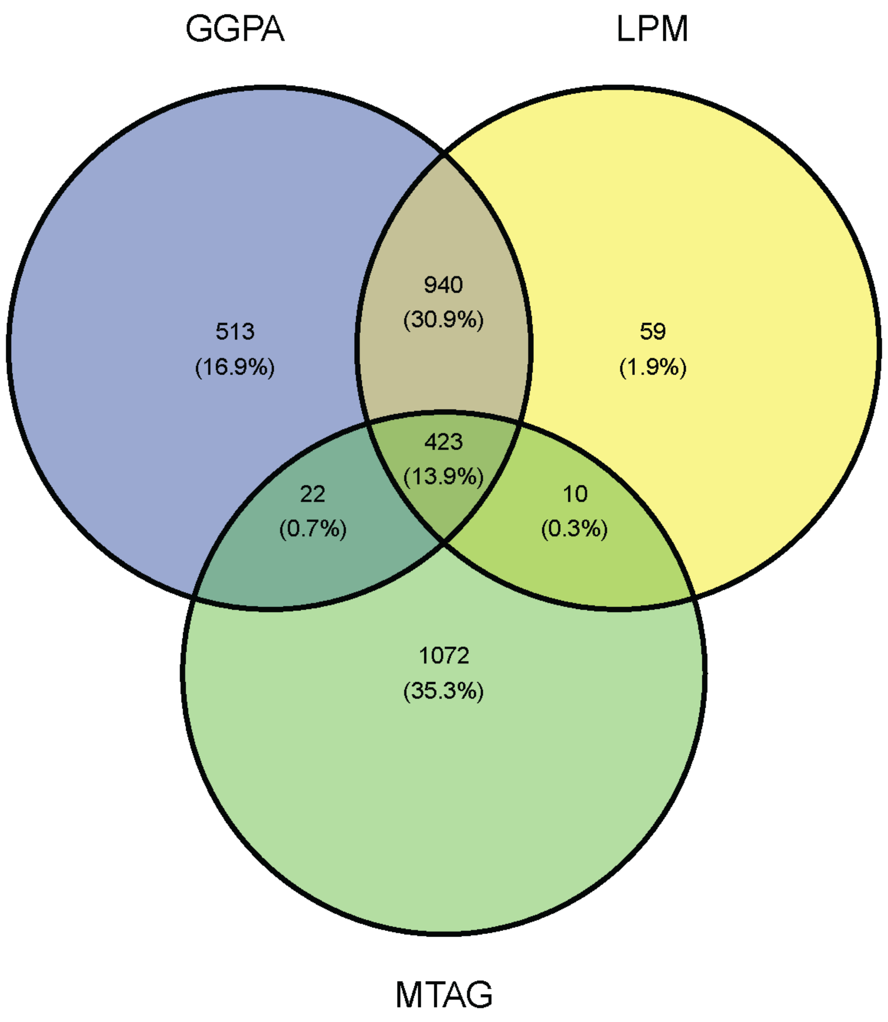

Figure S77: Venn diagram showing the overlap of the risk SNPs identified by GGPA 2.0, LPM, and MTAG at the nominal level of FDR 0.05 for CD.

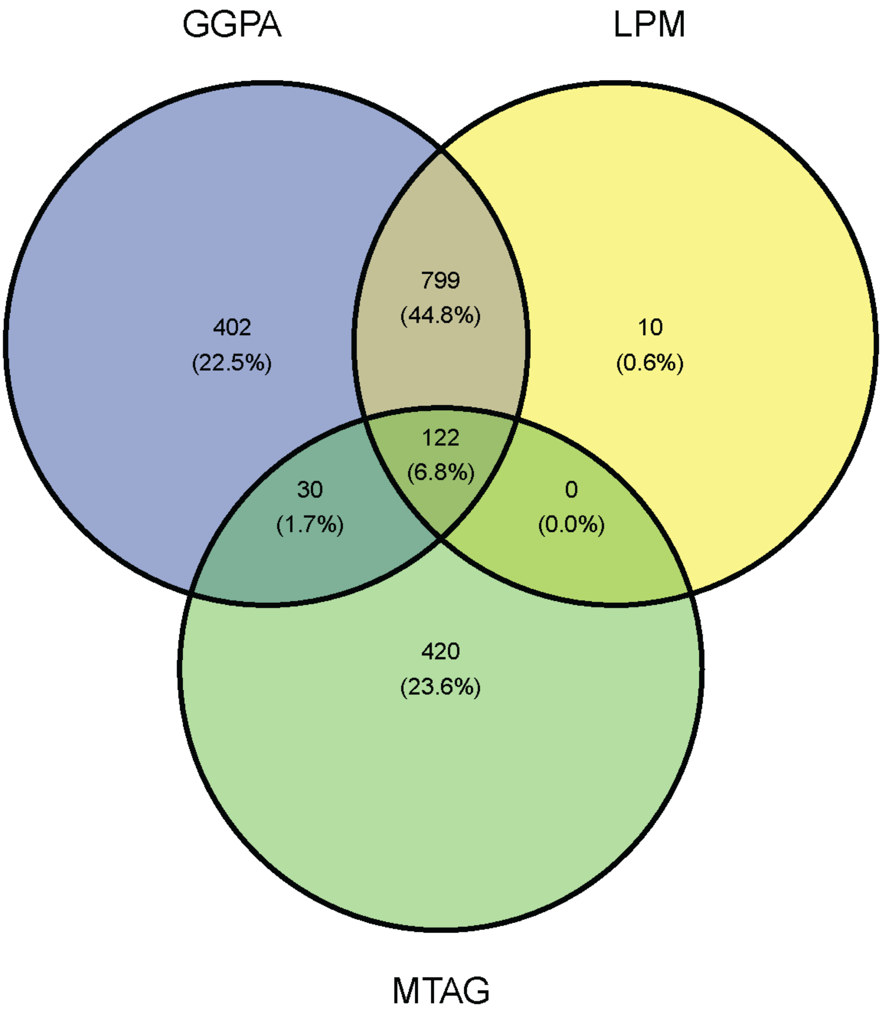

Figure S78: Venn diagram showing the overlap of the risk SNPs identified by GGPA 2.0, LPM, and MTAG at the nominal level of FDR 0.05 for T1D.

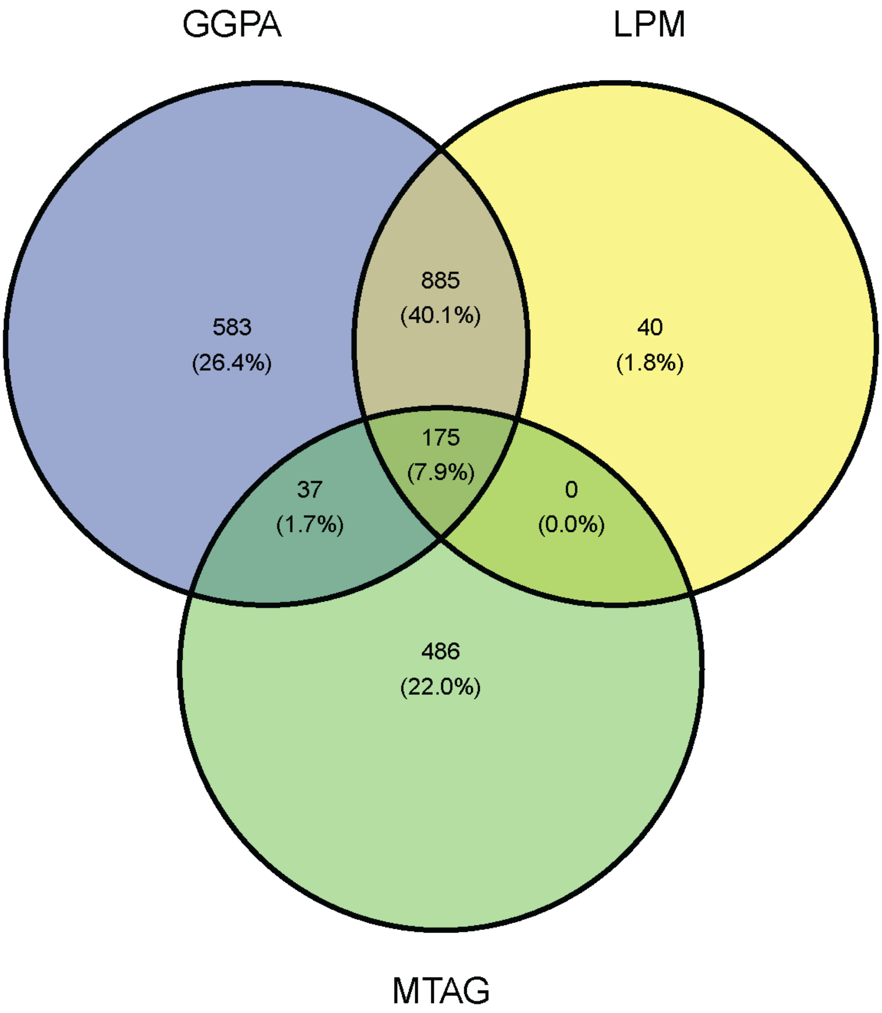

Figure S79: Venn diagram showing the overlap of the risk SNPs identified by GGPA 2.0, LPM, and MTAG at the nominal level of FDR 0.05 for SLE.

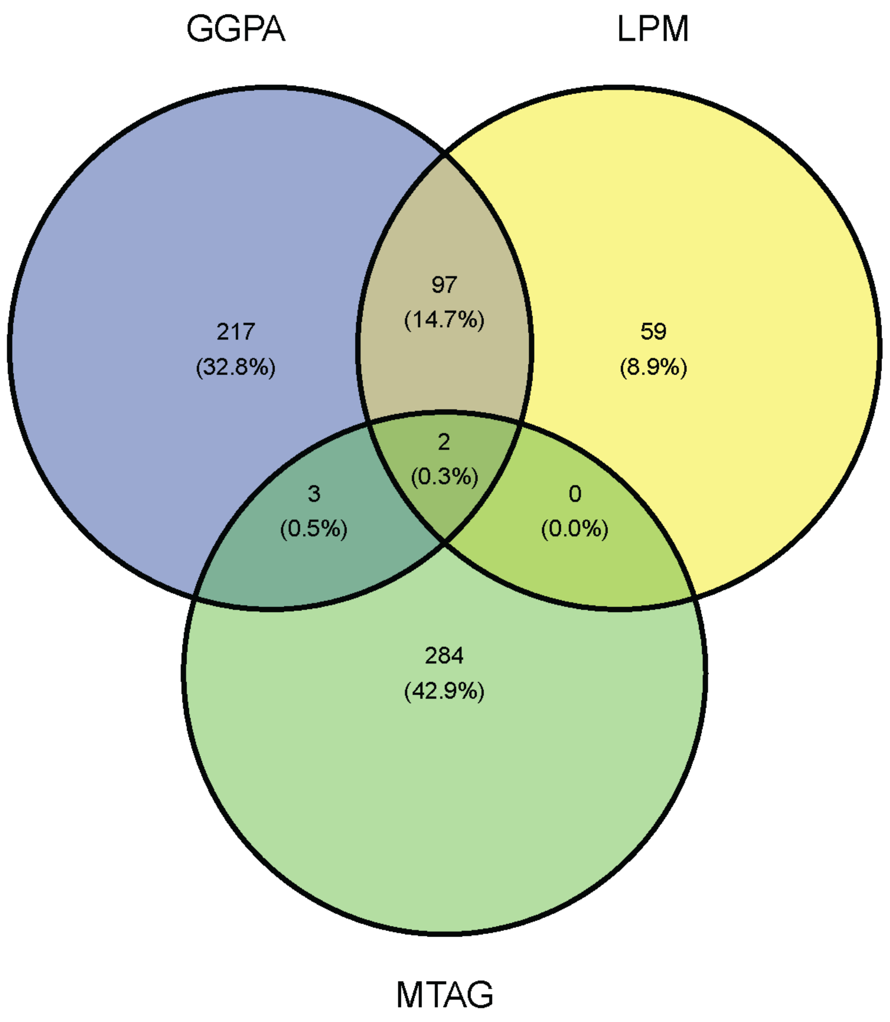

Figure S80: Venn diagram showing the overlap of the risk SNPs identified by GGPA 2.0, LPM, and MTAG at the nominal level of FDR 0.05 for ADHD.

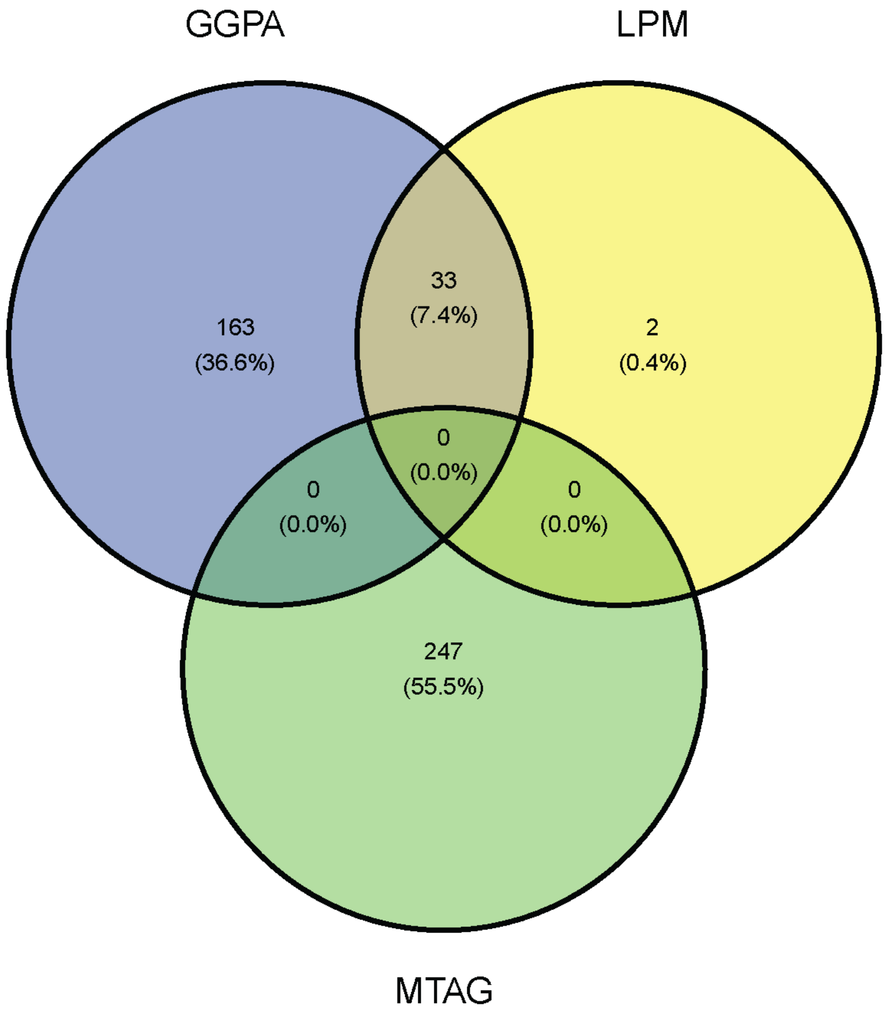

Figure S81: Venn diagram showing the overlap of the risk SNPs identified by GGPA 2.0, LPM, and MTAG at the nominal level of FDR 0.05 for ASD.

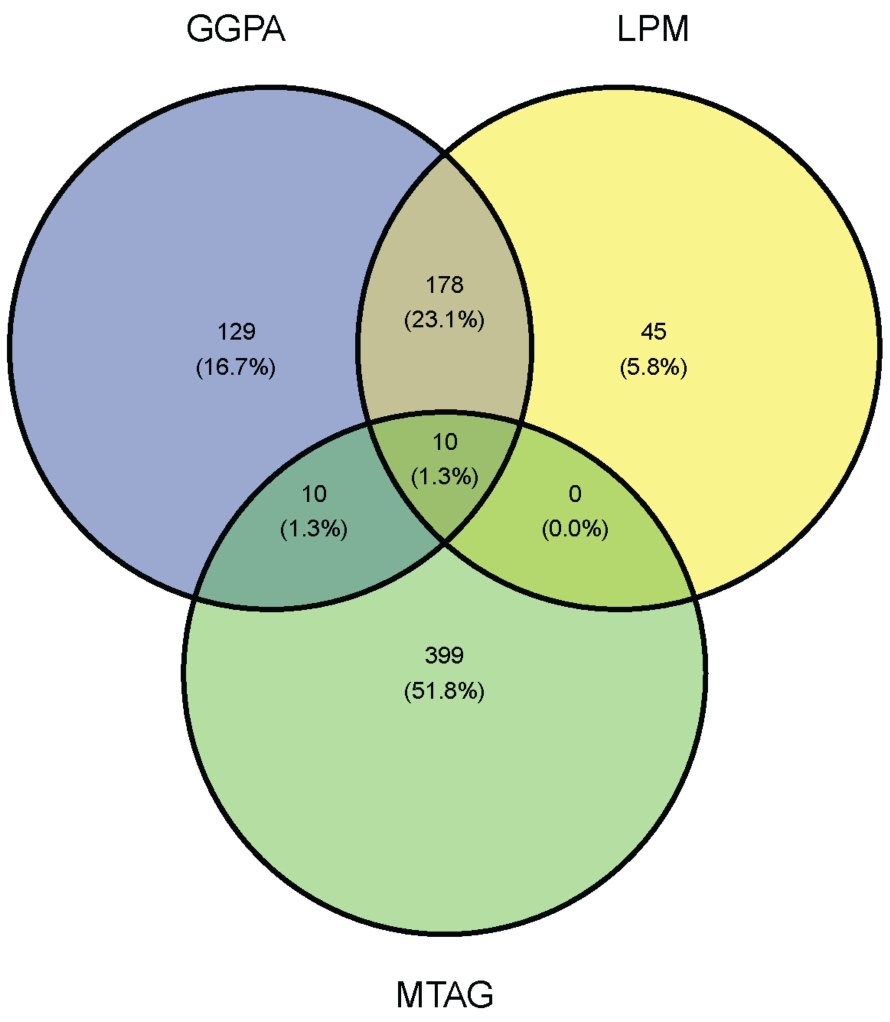

Figure S82: Venn diagram showing the overlap of the risk SNPs identified by GGPA 2.0, LPM, and MTAG at the nominal level of FDR 0.05 for MDD.

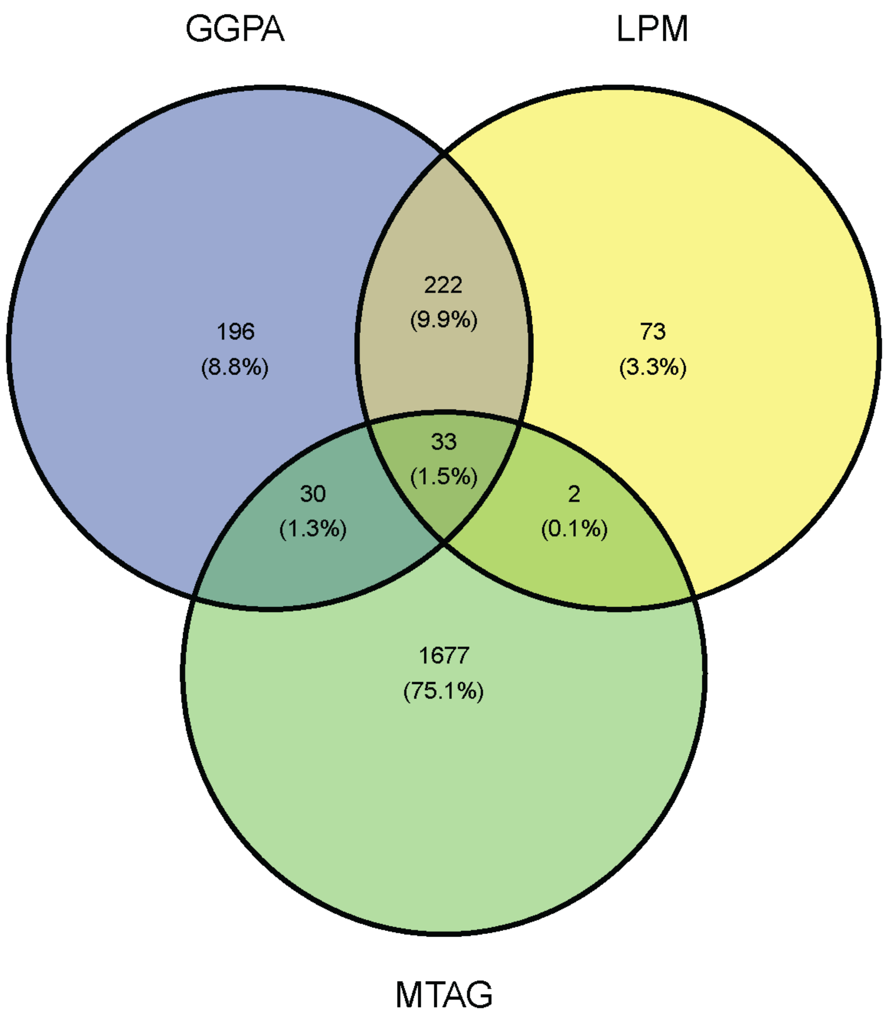

Figure S83: Venn diagram showing the overlap of the risk SNPs identified by GGPA 2.0, LPM, and MTAG at the nominal level of FDR 0.05 for BIP.

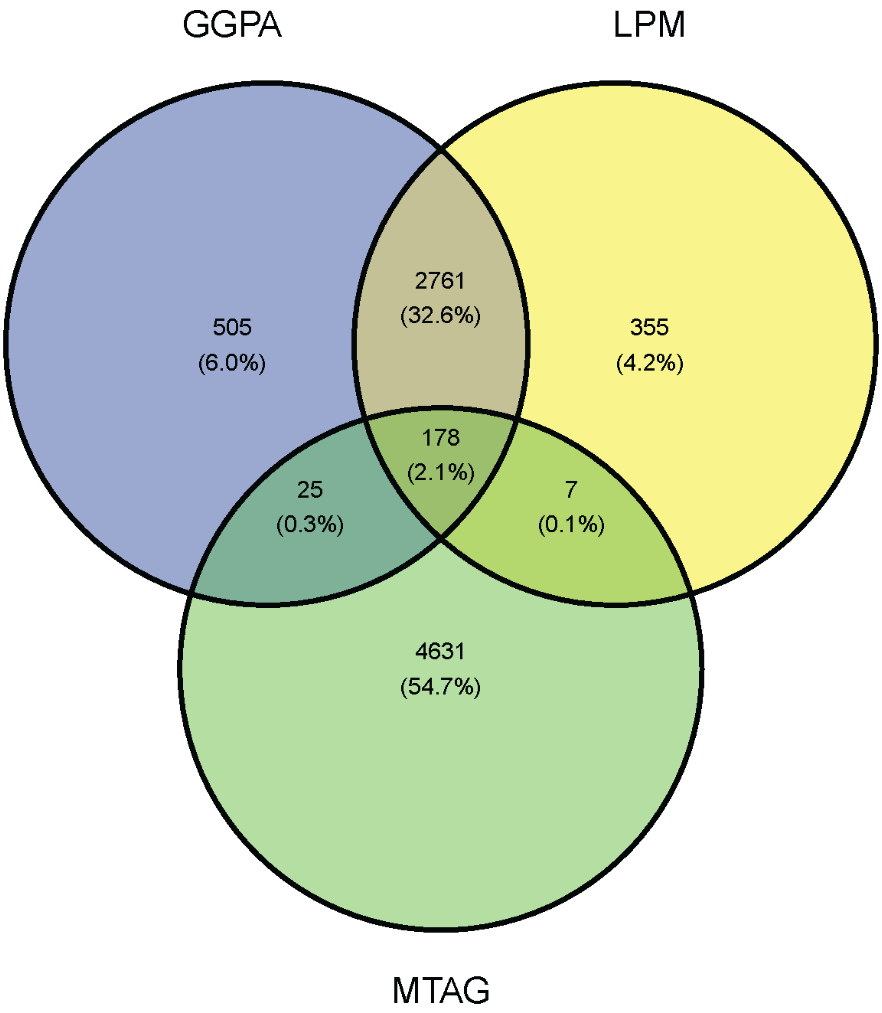

Figure S84: Venn diagram showing the overlap of the risk SNPs identified by GGPA 2.0, LPM, and MTAG at the nominal level of FDR 0.05 for SCZ.

REFERENCES

Bradfield, J. P., Qu, H.-Q., Wang, K., Zhang, H., Sleiman, P. M., Kim, C. E., et al. (2011). A genome-wide meta-analysis of six type 1 diabetes cohorts identifies multiple associated loci. *PLoS Genetics* 7, e1002293

De Lange, K. M., Moutsianas, L., Lee, J. C., Lamb, C. A., Luo, Y., Kennedy, N. A., et al. (2017). Genome-wide association study implicates immune activation of multiple integrin genes in inflammatory bowel disease. *Nature Genetics* 49, 256–261

Kim, H. J., Yu, Z., Lawson, A., Zhao, H., and Chung, D. (2018). Improving SNP prioritization and pleiotropic architecture estimation by incorporating prior knowledge using graph-GPA. *Bioinformatics* 34, 2139–2141

Langefeld, C. D., Ainsworth, H. C., Graham, D. S. C., Kelly, J. A., Comeau, M. E., Marion, M. C., et al. (2017). Transancestral mapping and genetic load in systemic lupus erythematosus. *Nature communications* 8, 1–18

Lee, P. H., Anttila, V., Won, H., Feng, Y.-C. A., Rosenthal, J., Zhu, Z., et al. (2019). Genomic relationships, novel loci, and pleiotropic mechanisms across eight psychiatric disorders. *Cell* 179, 1469–1482

Lu, Q., Powles, R. L., Abdallah, S., Ou, D., Wang, Q., Hu, Y., et al. (2017). Systematic tissue-specific functional annotation of the human genome highlights immune-related dna elements for late-onset alzheimer's disease. *PLoS Genetics* 13, e1006933

Lu, Q., Powles, R. L., Wang, Q., He, B. J., and Zhao, H. (2016). Integrative tissue-specific functional annotations in the human genome provide novel insights on many complex traits and improve signal prioritization in genome wide association studies. *PLoS Genetics* 12, e1005947

Okada, Y., Wu, D., Trynka, G., Raj, T., Terao, C., Ikari, K., et al. (2014). Genetics of rheumatoid arthritis contributes to biology and drug discovery. *Nature* 506, 376–381
